# Design, synthesis, and insecticidal activity of a novel series of flupyrimin analogs bearing 1-aryl-1H-pyrazol-4-yl subunits

**DOI:** 10.3389/fchem.2022.1019573

**Published:** 2022-10-03

**Authors:** Fenghai Zhao, Xianjun Tang, Jiaxing Huang, Jiaqi Li, Yumei Xiao, Zhaohai Qin

**Affiliations:** College of Science, China Agricultural University, Beijing, China

**Keywords:** synthesis, design, insecticidal activity, DFT calculation, molecular docking

## Abstract

To discover new potential insecticides to protect agricultural crops from damage, a series of novel flupyrimin derivatives containing an arylpyrazole core were designed and synthesized. Their structures were confirmed by ^1^H NMR, ^13^C NMR, and HRMS. Bioassays indicated that the 31 compounds synthesized possessed excellent insecticidal activity against *Plutella xylostella*. Among these target compounds, the lethality of **A3**, **B1-B6**, **D4**, and **D6** reached 100% at 400 μg/ml. Moreover, when the concentration dropped to 25 μg/ml, the insecticidal activities against the *Plutella xylostella* for compounds **B2**, **B3**, and **B4** still reached more than 70%. The structure–activity relationship of the *Plutella xylostella* was discussed. The density functional theory analysis of flupyrimin and **B4** was carried out to support the abovementioned structure–activity relationship. The possible binding modes between receptor and active groups in title compounds were also verified by docking simulation. These results provided new ideas for the development of these novel candidate insecticides in the future.

## Introduction

**FIGURE 1:**
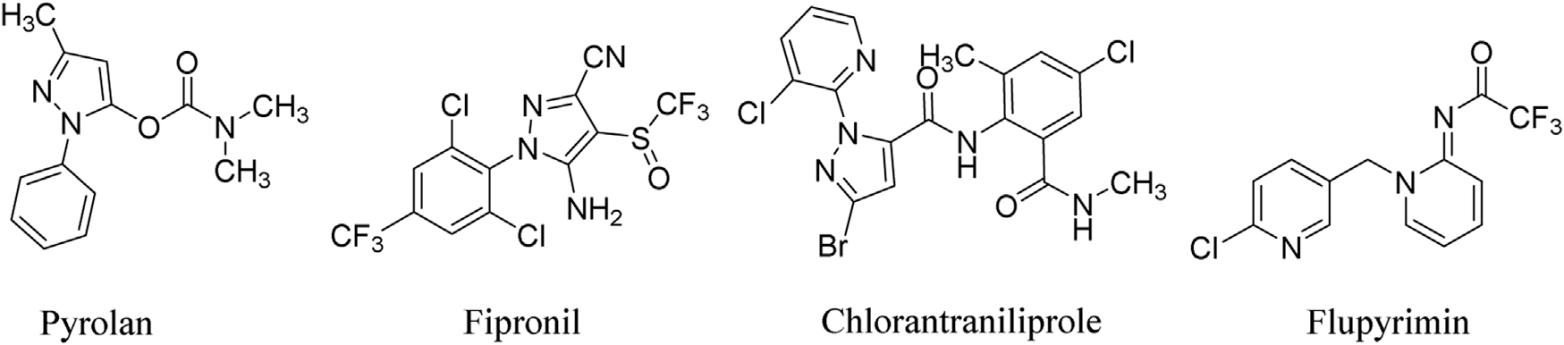
Structures of representative pesticides containing pyrazole moiety.

Pyrazole derivatives play an important role in agro-bioactive substances and receive extensive attention from scientists. As insecticides, it can be traced back to pyrolan, isolan, and dimetilan developed by Ciba-Geigy company in the 1940s (Ferguson and Alexander, 1953; Weiden and Moorefield, 1965) (Figure 1). Nowadays, insecticides bearing the pyrazole subunit have become a very important structural type. As a representative one, 1-phenylpyrazoles, such as fipronil, ethiprole, and butene fipronil, target the chloride channel of the GABA receptor in insects and exhibit excellent insecticidal activity against both sensitive and resistant pests (Caboni et al., 2003; Teicher et al., 2003; Ikeda et al., 2004; Arain et al., 2014; Lin et al., 2016). In 1998, the first ryanodine receptor modulator, flubendiamide, was developed by Nihon Nohyaku Co., Ltd., as a highly effective insecticide against almost all Lepidopteran pests, which quickly set off an upsurge of research on bisamide insecticides (Tohnishi et al., 2005). In order to avoid the patents protection scope of Nohyaku and Bayer company by adopting functional group interconversion (Jones, 2020) and scaffold hopping strategy (Schneider et al., 1999) and introducing 1-(pyrid-2-yl)pyrazole subunit, the more effective insecticide, chlorantraniliprole, is developed by DuPont company in 2007 (Figure 1). Interestingly, 1-(pyrid-2-yl)pyrazole almost become the standard configuration of this series of new pesticides, such as cyantraniliprole, cyclaniliprole, and so on (Lahm et al., 2005; Lahm et al., 2007; Feng et al., 2010).

Neonicotinoids are another excellent insecticide developed after organophosphorus and pyrethroids, which have played a decisive role in agricultural pest control for over 30 years. Although the application of traditional neonicotinoid insecticides is more and more restricted because of their safety for honeybees (Manjon et al., 2018), the development of novel neonicotinoid insecticides with better environmental compatibility has never stopped (Kamel, 2010; Zhang et al., 2021). Flupyrimin developed by Meiji Seika Kaisha Ltd., is a new outstanding representative of this kind of product, which shows high insecticidal activity against imidacloprid-resistant pests and low toxicity to non-target organisms such as honeybees (Onozaki et al., 2017; Terajima et al., 2021) (Figure 1).

**FIGURE 2:**
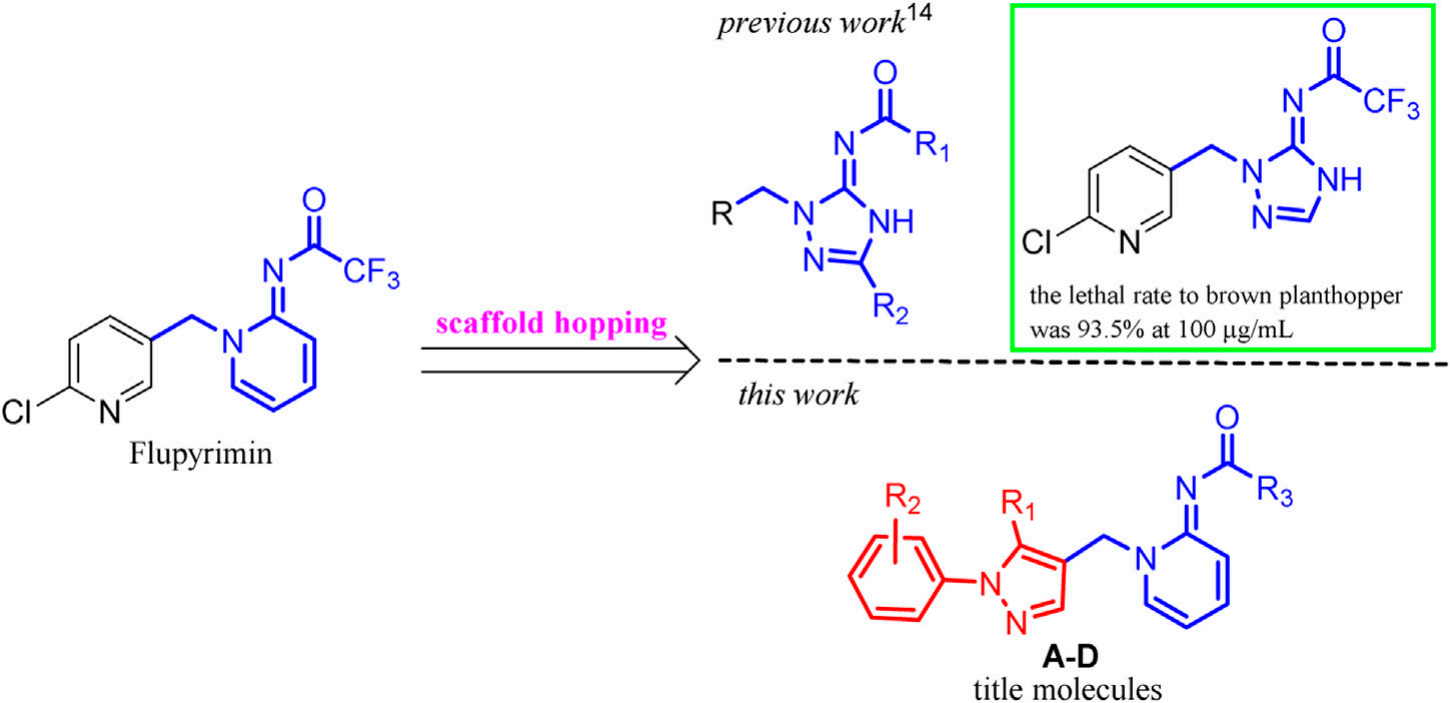
Design strategy of the title compounds.

For the continuous interest in developing new neonicotinoid insecticides with high efficiency and good environmental safety, we previously designed and synthesized triazole analogs of flupyrimin based on the scaffold hopping strategy (Zhao et al., 2022). However, they showed high activity against the *Nilaparvata lugens* at a concentration of 100 μg/ml, their efficiency was far lower than that of flupyrimin and imidacloprid at lower concentrations. In this article, a novel series of flupyrimin analogs **A–D** bearing 1-aryl-pyzazol-4-yl subunits was designed, synthesized, and screened based on the scaffold hopping strategy (Figure 2). It was expected to provide new insights into the development of novel insecticides with better activity and a unique mode of action.

## Materials and methods

### Chemicals, reagents, and instrumentation

All reagents were of analytical grade for all reactions and were purchased commercially without further purification. The melting points were measured on an X-4 micro melting point instrument and were uncorrected. NMR spectra were recorded on a Bruker Avance DPX (500 MHz) instrument in DMSO-*d*_*6*_ or CDCl_3_ using tetramethylsilane as an internal standard. HRMS data were obtained with a Thermo Scientific Q Exactive. The single crystal structure analysis was performed on an Agilent Gemini E dual-light source X-ray single crystal diffractometer.

### General synthesis

General procedures for preparing intermediates **(3)** *.* Ethyl 1-aryl-*1H*-pyrazole-4-carboxylates (**2**) were obtained according to previously reported procedures (Sturbaut et al., 2021); 13.0 mmol of **2** was dissolved in 30 ml of anhydrous THF. At 0°C, 0.58 g (15.6 mmol) of lithium aluminum hydride was slowly added, and the mixture was stirred for 1 h in an ice bath. After the reduction was completed, it was quenched with ice and extracted with ethyl acetate (3 × 30 ml). The combined organic layer was dried over anhydrous sodium sulfate for 3 h and then filtered. The filtrate was condensed to dryness in vacuo, and the residue was purified by column chromatography (hexane: EtOAc = 1:1) to give compound **3**.

General procedures for preparing intermediates **(4)** *.* First, 10.0 mmol of **3** was dissolved in 20 ml CH_2_Cl_2_, and 3 drops of DMF and 12.0 mmol of thionyl chloride were added. The mixture was refluxed for 2 h and the solvent was removed under reduced pressure, and the residue was purified by column chromatography (hexane: EtOAc = 5:1) to give intermediate **4**.

General procedures for preparing intermediates **(6)**. To 46.0 mmol of acid **5** in CH_2_Cl_2_ (5 ml), 50.0 mmol of thionyl chloride was added followed by 3 drops of DMF at 0°C. The mixture was stirred at room temperature for 12 h and then was concentrated in vacuo to give the corresponding acyl chloride without further purification; proceed directly to the next step.

To a solution of 2-aminopyridine (30.0 mmol) and 2 drops of triethylamine in 30 ml CH_2_Cl_2_, acyl chloride was added (30.0 mmol) dropwise under an ice bath and then allowed to react for 20 min at room temperature. The mixture was washed with saturated Na_2_CO_3_ solution and water, and the organic phase was dried over Na_2_SO_4_ and then concentrated. The residue was subjected to column chromatography to afford intermediate **6**.

General procedures for preparing target compounds (**A–D**). A mixture of 20.0 mmol intermediate **4**, 20.0 mmol **6**, 40.0 mmol of K_2_CO_3,_ and 30 ml acetonitrile was refluxed at 90 °C for 5 h. The solvent was removed in vacuo, and the residue was extracted with EtOAc (3 × 30 ml). The combined organic layer was dried over Na_2_SO_4_, filtered, and concentrated. The residue was purified by column chromatography (hexane: EtOAc = 4:1) to afford **A–D**.

Data for (***A1***) (*E*)-2,2,2-trifluoro-*N*-(1-((1-phenyl-5-(trifluoromethyl)-*1H*-pyrazol-4-yl)methyl)pyridin-2(*1H*)-ylidene)acetamide*.* White powder, m. p. 168–169°C, yields 70%. ^1^H NMR (500 MHz, CDCl_3_) δ 8.56 (dd, *J* = 9.1, 1.4 Hz, 1H), 7.97 (s, 1H), 7.85 (dd, *J* = 6.7, 1.8 Hz, 1H), 7.79 (ddd, *J* = 9.0, 6.9, 1.8 Hz, 1H), 7.51–7.45 (m, 3H), 7.43–7.37 (m, 2H), 6.88 (td, *J* = 6.8, 1.4 Hz, 1H), and 5.62 (s, 2H). ^13^C NMR (126 MHz, CDCl_3_) δ 162.80 (q, *J*_*CF*_ = 37.5 Hz), 158.18, 140.95, 140.90, 138.02, 137.46, 128.76, 128.74 (q, *J*_*CF*_ = 37.5 Hz), 128.11, 124.98, 122.47, 121.14, 120.33, 119.77, 118.18, 117.48, 116.37, 116.03, 115.19, 113.66, 112.90, and 45.58. HRMS (ESI) *m/z*: calcd. for C_18_H_12_F_6_N_4_O [M + H]^+^, 415.0988; found, 415.0988.

Data for (***A2***) (*(E)*)-*N*-(1-((1-(3-chlorophenyl)-5-(trifluoromethyl)-*1H*-pyrazol-4-yl)methyl)pyridin-2(*1H*)-ylidene)-2,2,2-trifluoroacetamide. White powder, m.p.131–132°C, yields 74%. ^1^H NMR (500 MHz, CDCl_3_) δ 8.57 (d, *J* = 9.0 Hz, 1H), 8.02 (s, 1H), 7.87–7.82 (m, 1H), 7.80 (ddd, *J* = 8.9, 6.9, 1.8 Hz, 1H), 7.54 (d, *J* = 8.0 Hz, 1H), 7.48 (dt, *J* = 8.3, 4.0 Hz, 1H), 7.40 (d, *J* = 4.5 Hz, 2H), 6.88 (t, *J* = 6.8 Hz, 1H), and 5.61 (q, 2H). ^13^C NMR (126 MHz, CDCl_3_) δ 162.85 (q, *J*_*CF*_ = 37.5 Hz), 158.19, 141.27, 140.94, 137.36, 135.41, 131.87, 130.67, 129.85 (q, *J*_*CF*_ = 37.5 Hz), 129.30, 128.32, 126.40, 122.16, 121.16, 120.01, 119.75, 117.86, 117.46, 115.97, 115.71, 115.17, 113.69, 112.88, and 45.39. HRMS (ESI) *m/z*: calcd. for C_18_H_11_ClF_6_N_4_O [M + H]^+^, 449.0598; found, 449.0597.

Data for (***A3***) (*E*)-2,2,2-trifluoro-*N*-(1-((1-(3-fluorophenyl)-5-(trifluoromethyl)-*1H*-pyrazol-4-yl)methyl)pyridin-2(*1H*)-ylidene)acetamide*.* White powder, m. p. 154–155°C, yields 81%. ^1^H NMR (500 MHz, CDCl_3_) δ 8.56 (dd, *J* = 9.1, 1.4 Hz, 1H), 7.99 (s, 1H), 7.85 (dd, *J* = 6.7, 1.8 Hz, 1H), 7.80 (ddd, *J* = 9.0, 6.9, 1.8 Hz, 1H), 7.46 (td, *J* = 8.2, 6.0 Hz, 1H), 7.21 (td, *J* = 8.4, 2.4 Hz, 2H), 7.16 (dt, *J* = 9.1, 2.3 Hz, 1H), 6.89 (td, *J* = 6.8, 1.4 Hz, 1H), and 5.61 (s, 2H). ^13^C NMR (126 MHz, CDCl_3_) δ 162.85 (q, *J*_*CF*_ = 37.5 Hz), 162.39, 162.33, 160.35, 158.17, 141.32, 140.98, 139.05 (d, *J*_*CF*_ = 12.5 Hz), 137.47, 129.45, 129.38, 128.65 (q, *J*_*CF*_ = 37.5 Hz), 122.37, 121.17, 120.76, 120.73, 120.22, 119.74, 118.07, 117.45, 116.87, 116.02, 115.92, 115.85, 115.16, 113.69, 112.89, 112.70, and 45.59. HRMS (ESI) *m/z*: calcd. for C_18_H_11_F_7_N_4_O [M + H]^+^, 433.0894; found, 433.0892.

Data for (***A4***) (*E*)-*N*-(1-((1-(4-chlorophenyl)-5-(trifluoromethyl)-*1H*-pyrazol-4-yl)methyl)pyridin-2(*1H*)-ylidene)-2,2,2-trifluoroacetamide*.* White powder, m.p.159–160°C, yields 72%. ^1^H NMR (500 MHz, CDCl_3_) δ 8.56 (dd, *J* = 9.0, 1.3 Hz, 1H), 7.84 (dd, *J* = 6.7, 1.8 Hz, 1H), 7.80 (ddd, *J* = 8.9, 6.9, 1.8 Hz, 1H), 7.46 (d, *J* = 8.7 Hz, 2H), 7.35 (d, *J* = 8.7 Hz, 2H), 6.88 (td, *J* = 6.8, 1.4 Hz, 1H), and 5.61 (s, 2H). ^13^C NMR (126 MHz, CDCl_3_) δ 162.85 (q, *J*_*CF*_ = 37.5 Hz), 158.17, 141.28, 140.97, 137.47, 136.46, 134.84, 128.70 (q, *J*_*CF*_ = 37.5 Hz), 128.38, 126.25, 122.39, 121.17, 120.24, 119.74, 118.09, 117.45, 116.77, 115.94, 115.16, 113.68, 112.87, and 45.62. HRMS (ESI) *m/z*: calcd. for C_18_H_11_ClF_6_N_4_O [M + H]^+^, 449.0598; found, 449.0596.

Data for (***A5***) (*E*)-2,2,2-trifluoro-*N*-(1-((5-(trifluoromethyl)-1-(4-(trifluoromethyl)phenyl)-*1H*-pyrazol-4-yl)methyl)pyridin-2(*1H*)-ylidene)acetamide*.* Yellowish powder, m. p. 142–143 °C, yield 72%. ^1^H NMR (500 MHz, CDCl_3_) δ 8.57 (dd, *J* = 9.1, 1.4 Hz, 1H), 8.02 (s, 1H), 7.85 (dd, *J* = 6.6, 1.8 Hz, 1H), 7.81 (ddd, *J* = 8.9, 7.0, 1.8 Hz, 1H), 7.76 (d, *J* = 8.4 Hz, 2H), 7.56 (d, *J* = 8.3 Hz, 2H), 6.90 (td, *J* = 6.8, 1.5 Hz, 1H), 5.62 (s, 2H). ^13^C NMR (126 MHz, CDCl_3_) δ 163.85 (q, *J*_*CF*_ = 37.5 Hz), 159.24, 142.72, 142.08, 141.83, 138.54, 131.85 (q, *J*_*CF*_ = 37.5 Hz), 129.75 (q, *J*_*CF*_ = 37.5 Hz), 126.50, 126.47, 126.44, 126.41, 126.34, 124.58, 123.42, 122.41, 122.24, 121.27, 120.78, 120.25, 119.12, 118.49, 118.35, 116.97, 116.20, 114.74, 113.91, 46.69. HRMS (ESI) *m/z*: calcd. for C_19_H_11_F_9_N_4_O [M + H]^+^, 483.0862; found, 483.0860.

Data for (***A6***) (*E*)-*N*-(1-((1-(3-chlorophenyl)-5-(difluoromethyl)-*1H*-pyrazol-4-yl)methyl)pyridin-2(*1H*)-ylidene)-2,2,2-trifluoroacetamide*.* White powder, m. p. 124–125°C, yields 83%. ^1^H NMR (500 MHz, CDCl_3_) δ 8.53 (dd, *J* = 9.1, 1.4 Hz, 1H), 8.05 (s, 1H), 7.97 (dd, *J* = 6.7, 1.8 Hz, 1H), 7.78 (ddd, *J* = 9.0, 6.9, 1.8 Hz, 1H), 7.49–7.40 (m, 3H), 7.31 (dt, *J* = 6.9, 2.1 Hz, 1H), and 6.93–6.67 (m, 2H). ^13^C NMR (126 MHz, CDCl_3_) δ 163.70 (q, *J*_*CF*_ = 37.5 Hz), 159.04, 142.80, 141.93, 139.21, 139.02, 135.43, 133.51 (t, *J*_*CF*_ = 25.0 Hz), 130.59, 129.68, 125.69, 123.31, 122.02, 120.88, 118.58, 117.03, 116.29, 114.76, 114.00, 110.98, 109.11, 107.24, and 46.37. HRMS (ESI) *m/z*: calcd. for C_18_H_12_ClF_5_N_4_O [M + H]^+^, 431.0693; found, 431.0692.

Data for (***A7***) (*E*)-*N*-(1-((5-(difluoromethyl)-1-(3-fluorophenyl)-*1H*-pyrazol-4-yl)methyl)pyridin-2(*1H*)-ylidene)-2,2,2-trifluoroacetamide. White powder, m. p. 127–128°C, yields 69%. ^1^H NMR (500 MHz, CDCl_3_) δ 8.52 (d, *J* = 9.0 Hz, 1H), 8.05 (s, 1H), 7.98 (dd, *J* = 6.6, 1.8 Hz, 1H), 7.77 (ddd, *J* = 8.9, 7.0, 1.8 Hz, 1H), 7.51–7.44 (m, 1H), 7.24–7.15 (m, 3H), 6.94–6.70 (m, 2H), and 5.63 (s, 2H). ^13^C NMR (126 MHz, CDCl_3_) δ 163.78, 163.70 (q, *J*_*CF*_ = 37.5 Hz), 161.79, 159.02, 142.75, 141.99, 139.54, 139.46, 139.11, 133.50 (t, *J*_*CF*_ = 25.0 Hz), 131.01, 130.94, 121.97, 120.91, 120.83, 120.81, 118.61, 117.07, 116.67, 116.51, 116.32, 114.84, 114.03, 113.16, 112.97, 111.02, 109.15, 107.28, and 46.39. HRMS (ESI) *m/z*: calcd. for C_18_H_12_F_6_N_4_O [M + H]^+^, 415.0988; found, 415.0985.

Data for (***A8***) (*E*)-*N*-(1-((5-(difluoromethyl)-1-(4-fluorophenyl)-*1H*-pyrazol-4-yl)methyl)pyridin-2(*1H*)-ylidene)-2,2,2-trifluoroacetamide*.* White powder, m. p. 148–149°C, yields 71%. ^1^H NMR (500 MHz, CDCl_3_) δ 8.54 (dd, *J* = 9.1, 1.4 Hz, 1H), 8.04 (s, 1H), 7.98 (dd, *J* = 6.8, 1.8 Hz, 1H), 7.79 (ddd, *J* = 9.0, 7.0, 1.8 Hz, 1H), 7.45–7.39 (m, 2H), 7.24–7.17 (m, 2H), 6.92–6.64 (m, 2H), and 5.64 (s, 2H). ^13^C NMR (126 MHz, CDCl_3_) δ 163.87, 163.70 (q, *J*_*CF*_ = 37.5 Hz), 161.87, 159.08, 142.47, 141.89, 139.00, 134.38, 134.36, 133.60 (t, *J*_*CF*_ = 25.0 Hz), 127.43, 127.36, 122.05, 120.88, 118.59, 116.75, 116.57, 116.30, 114.72, 114.01, 111.11, 109.24, 107.37, and 46.40. HRMS (ESI) *m/z*: calcd. for C_18_H_12_F_6_N_4_O [M + H]^+^, 415.0988; found, 415.0986.

Data for (***B1***) (*E*)-2-methoxy-*N*-(1-((1-phenyl-5-(trifluoromethyl)-*1H*-pyrazol-4-yl)methyl)pyridin-2(*1H*)-ylidene)acetamide*.* Colorless oil yields 42%. ^1^H NMR (500 MHz, CDCl_3_) δ 8.51 (dd, *J* = 4.9, 1.9 Hz, 1H), 7.81–7.72 (m, 2H), 7.48–7.42 (m, 3H), 7.41–7.35 (m, 2H), 7.27–7.18 (m, 2H), 5.15 (s, 2H), 4.12 (s, 2H), and 3.37 (s, 3H). ^13^C NMR (126 MHz, CDCl_3_) δ 169.83, 153.78, 149.27, 140.98, 139.36, 138.60, 129.37, 128.97, 128.70 (q, *J*_*CF*_ = 37.5 Hz), 125.99, 123.47, 122.64, 121.32, 120.42, 119.17, 117.02, 71.60, 59.24, and 41.57. HRMS (ESI) *m/z*: calcd. for C_19_H_17_F_3_N_4_O_2_ [M + H]^+^, 391.1376; found, 391.1374.

Data for (***B2***) (*E*)-*N*-(1-((1-(2-chlorophenyl)-5-(trifluoromethyl)-*1H*-pyrazol-4-yl)methyl)pyridin-2(*1H*)-ylidene)-2-methoxyacetamide. Yellowish oil yields 48%. ^1^H NMR (500 MHz, CDCl_3_) δ 8.49 (ddd, *J* = 4.9, 2.0, 0.8 Hz, 1H), 7.79 (s, 1H), 7.72 (td, *J* = 7.7, 2.0 Hz, 1H), 7.48 (dd, *J* = 8.1, 1.4 Hz, 1H), 7.42 (ddd, *J* = 8.1, 6.5, 2.5 Hz, 1H), 7.38–7.32 (m, 2H), 7.23 (ddd, *J* = 7.5, 4.9, 1.0 Hz, 1H), 7.14 (d, *J* = 8.0 Hz, 1H), 5.23–5.03 (m, 2H), 4.07 (d, *J* = 3.5 Hz, 2H), and 3.34 (s, 3H). ^13^C NMR (126 MHz, CDCl_3_) δ 169.69, 153.51, 149.37, 141.59, 138.54, 136.68, 133.11, 131.40, 130.16, 130.11 (q, *J*_*CF*_ = 37.5 Hz), 129.53, 127.25, 125.98, 123.11, 122.75, 120.96, 120.86, 120.42, 119.74, 118.81, 116.66, 71.52, 59.23, and 40.97. HRMS (ESI) *m/z*: calcd. for C_19_H_16_ClF_3_N_4_O_2_ [M + H]^+^, 425.0987; found, 425.0984.

Data for (***B3***) (*E*)-*N*-(1-((1-(3-fluorophenyl)-5-(trifluoromethyl)-*1H*-pyrazol-4-yl)methyl)pyridin-2(*1H*)-ylidene)-2-methoxyacetamide*.* Colorless oil yields 45%. ^1^H NMR (500 MHz, CDCl_3_) δ 8.51 (dd, *J* = 5.0, 1.9 Hz, 1H), 7.81–7.74 (m, 2H), 7.42 (td, *J* = 8.2, 6.0 Hz, 1H), 7.26–7.12 (m, 5H), 5.14 (s, 2H), 4.11 (s, 2H), and 3.36 (s, 3H). ^13^C NMR (126 MHz, CDCl_3_) δ 169.85, 163.34, 161.36, 153.77, 149.31, 141.42, 140.47 (d, *J*_*CF*_ = 10.0 Hz), 138.62, 130.26, 130.19, 128.96, 128.65, 123.36, 122.66, 121.69, 121.21, 120.95, 120.37, 119.07, 116.91, 116.56, 116.40, 113.84, 113.64, 71.60, 59.26, and 41.53. HRMS (ESI) *m/z*: calcd. for C_19_H_16_F_4_N_4_O_2_ [M + H]^+^, 409.1282; found, 409.1280.

Data for (***B4***) (*E*)-*N*-(1-((1-(4-chlorophenyl)-5-(trifluoromethyl)-*1H*-pyrazol-4-yl)methyl)pyridin-2(*1H*)-ylidene)-2-methoxyacetamide*.* Colorless oil yields 50%. ^1^H NMR (500 MHz, CDCl_3_) δ 8.50 (dd, *J* = 4.9, 1.2 Hz, 1H), 7.79–7.73 (m, 2H), 7.41 (d, *J* = 8.8 Hz, 2H), 7.32 (d, *J* = 8.7 Hz, 2H), 7.26–7.23 (m, 1H), 7.20 (d, *J* = 8.1 Hz, 1H), 5.12 (s, 2H), 4.10 (s, 2H), and 3.35 (s, 3H). ^13^C NMR (126 MHz, CDCl_3_) δ 169.85, 153.78, 149.29, 141.34, 138.62, 137.83, 135.38, 130.11 (q, *J*_*CF*_ = 37.5 Hz), 129.23, 127.24, 123.38, 122.66, 121.23, 120.84, 120.36, 119.08, 116.93, 71.59, 59.25, and 41.56. HRMS (ESI) *m/z*: calcd. for C_19_H_16_ClF_3_N_4_O_2_ [M + H]^+^, 425.0987; found, 425.0985.

Data for (***B5***) (*E*)-2-methoxy-*N*-(1-((5-(trifluoromethyl)-1-(4-(trifluoromethyl)phenyl)-*1H*-pyrazol-4-yl)methyl)pyridin-2(*1H*)-ylidene)acetamide*.* Colorless oil yields 39%. ^1^H NMR (500 MHz, CDCl_3_) δ 8.52 (dd, *J* = 5.0, 1.9 Hz, 1H), 7.83 (s, 1H), 7.79 (td, *J* = 7.8, 2.0 Hz, 1H), 7.73 (d, *J* = 8.3 Hz, 2H), 7.54 (d, *J* = 8.3 Hz, 2H), 7.28–7.21 (m, 2H), 5.15 (s, 2H), 4.12 (s, 2H), and 3.37 (s, 3H). ^13^C NMR (126 MHz, CDCl_3_) δ 169.91, 153.80, 149.34, 142.16, 141.82, 138.69, 131.33 (q, *J*_*CF*_ = 32.9 Hz), 128.84 (q, *J*_CF_ = 38.5 Hz), 126.84, 126.33, 126.30, 126.27, 126.24, 126.19, 124.67, 123.38, 122.73, 122.50, 121.46, 121.23, 120.35, 120.12, 119.09, 116.94, 71.61, 59.27, and 41.64. HRMS (ESI) *m/z*: calcd. for C_20_H_16_F_6_N_4_O_2_ [M + H]^+^, 459.1251; found, 459.1238.

Data for (***B6***) (*E*)-*N*-(1-((1-(3-chlorophenyl)-5-(difluoromethyl)-*1H*-pyrazol-4-yl)methyl)pyridin-2(*1H*)-ylidene)-2-methoxyacetamide*.* Colorless oil yields 34%. ^1^H NMR (500 MHz, CDCl_3_) δ 8.51 (dd, *J* = 5.1, 1.9 Hz, 1H), 7.75 (td, *J* = 7.7, 2.0 Hz, 1H), 7.61 (s, 1H), 7.48 (s, 1H), 7.42–7.33 (m, 3H), 7.26–7.13 (m, 2H), 6.81 (t, *J* = 52.6 Hz, 1H), 5.10 (s, 2H), 4.06 (s, 2H), and 3.33 (s, 3H). ^13^C NMR (126 MHz, CDCl_3_) δ 169.72, 153.64, 149.37, 141.53, 140.18, 138.60, 134.92, 132.71 (t, *J*_*CF*_ = 27.4 Hz), 130.14, 129.05, 125.67, 123.34, 122.69, 120.72, 120.03, 110.33, 108.46, 106.58, 71.53, 59.22, and 40.83. HRMS (ESI) *m/z*: calcd. for C_19_H_17_ClF_2_N_4_O_2_ [M + H]^+^, 407.1081; found, 407.1080.

Data for (***B7***) (*E*)-*N*-(1-((5-(difluoromethyl)-1-(3-fluorophenyl)-*1H*-pyrazol-4-yl)methyl)pyridin-2(*1H*)-ylidene)-2-methoxyacetamide*.* Yellowish oil yields 43%. ^1^H NMR (500 MHz, CDCl_3_) δ 8.52 (dd, *J* = 5.1, 1.9 Hz, 1H), 7.77 (td, *J* = 7.7, 1.9 Hz, 1H), 7.63 (s, 1H), 7.43 (td, *J* = 8.2, 6.0 Hz, 1H), 7.27–7.11 (m, 5H), 6.83 (t, *J* = 52.6 Hz, 1H), 5.12 (s, 2H), 4.08 (s, 2H), and 3.35 (s, 3H). ^13^C NMR (126 MHz, CDCl_3_) δ 169.72, 163.58, 161.60, 153.66, 149.35, 141.49, 140.43 (d, *J*_*CF*_ = 9.9 Hz), 138.58, 132.63 (t, *J*_*CF*_ = 27.4 Hz), 130.47, 130.40, 122.67, 120.83, 120.80, 120.71, 120.04, 115.99, 115.82, 113.08, 112.88, 110.36, 108.49, 106.61, 71.53, 59.21, and 40.85. HRMS (ESI) *m/z*: calcd. for C_19_H_17_F_3_N_4_O_2_ [M + H]^+^, 391.1376; found, 391.1375.

Data for (***B8***) (*E*)-*N*-(1-((5-(difluoromethyl)-1-(4-fluorophenyl)-*1H*-pyrazol-4-yl)methyl)pyridin-2(*1H*)-ylidene)-2-methoxyacetamide*.* Yellowish oil yields 55%. ^1^H NMR (500 MHz, CDCl_3_) δ 8.52 (ddd, *J* = 4.9, 2.0, 0.8 Hz, 1H), 7.77 (td, *J* = 7.7, 2.0 Hz, 1H), 7.60 (s, 1H), 7.45–7.40 (m, 2H), 7.27–7.24 (m, 1H), 7.22–7.12 (m, 3H), 6.79 (t, *J* = 52.7 Hz, 1H), 5.11 (s, 2H), 4.08 (s, 2H), and 3.35 (s, 3H). ^13^C NMR (126 MHz, CDCl_3_) δ 169.70, 163.55, 161.56, 153.70, 149.35, 141.15, 138.57, 135.35 (d, *J*_*CF*_ = 3.1 Hz), 132.82 (t, *J*_*CF*_ = 27.4 Hz), 127.39, 127.32, 122.65, 120.73, 119.55, 116.23, 116.05, 110.42, 108.55, 106.67, 71.54, 59.21, and 40.85. HRMS (ESI) *m/z*: calcd. for C_19_H_17_F_3_N_4_O_2_ [M + H]^+^, 391.1376; found, 391.1375.

Data for (***C1***) (*E*)-2,2,3,3,3-pentafluoro-*N*-(1-((1-phenyl-5-(trifluoromethyl)-*1H*-pyrazol-4-yl)methyl)pyridin-2(*1H*)-ylidene)propanamide*.* White powder, m. p. 162–163°C, yields 84%. ^1^H NMR (500 MHz, CDCl_3_) δ 8.58 (dd, *J* = 8.9, 1.4 Hz, 1H), 7.87–7.77 (m, 3H), 7.53–7.45 (m, 3H), 7.40 (dd, *J* = 7.5, 2.4 Hz, 2H), 6.90 (td, *J* = 6.9, 1.5 Hz, 1H), and 5.63 (s, 2H). ^13^C NMR (126 MHz, CDCl_3_) δ 164.19 (t, *J*_*CF*_ = 24.7 Hz), 158.78, 142.21, 141.26, 139.04, 138.58, 131.33 (q, *J*_*CF*_ = 32.9 Hz), 129.84, 129.18, 126.01, 122.48, 122.20, 121.34, 120.49, 120.21, 119.92, 119.19, 118.22, 117.93, 117.63, 117.04, 115.04, 110.01, 109.71, 107.78 (q, *J*_*CF*_ = 37.4 Hz), 105.85, 105.55, 46.60, 46.58, 46.57, and 46.55. HRMS (ESI) *m/z*: calcd. for C_19_H_12_F_8_N_4_O [M + H]^+^, 465.0956; found, 465.0956.

Data for (***C2***) (*E*)-*N*-(1-((1-(2-chlorophenyl)-5-(trifluoromethyl)-*1H*-pyrazol-4-yl)methyl)pyridin-2(*1H*)-ylidene)-2,2,3,3,3-pentafluoropropanamide*.* White powder, m. p. 155–156 °C, yields 77%. ^1^H NMR (500 MHz, CDCl_3_) δ 8.60 (dd, *J* = 9.6, 1.5 Hz, 1H), 7.88 (s, 1H), 7.85–7.80 (m, 2H), 7.54 (dt, *J* = 8.1, 1.0 Hz, 1H), 7.52–7.46 (m, 1H), 7.43–7.37 (m, 2H), 6.90 (td, *J* = 6.8, 1.5 Hz, 1H), and 5.64 (s, 2H). ^13^C NMR (126 MHz, CDCl_3_) δ 164.27 (t, *J*_*CF*_ = 24.7 Hz), 158.82, 142.18, 141.64, 138.39, 136.41, 132.90, 131.74, 130.72 (q, *J*_*CF*_ = 38.0 Hz), 130.35, 129.36, 127.45, 123.16, 122.25, 121.01, 120.47, 120.18, 119.90, 118.86, 118.20, 117.91, 117.62, 117.15, 116.71, 114.99, 109.97, 109.68, 107.74 (q, *J*_*CF*_ = 36.9 Hz), 105.81, 105.51, and 46.34. HRMS (ESI) *m/z*: calcd. for C_19_H_11_ClF_8_N_4_O [M + H]^+^, 499.0566; found, 499.0565.

Data for (***C3***) (*E*)-2,2,3,3,3-pentafluoro-*N*-(1-((1-(3-fluorophenyl)-5-(trifluoromethyl)-*1H*-pyrazol-4-yl)methyl)pyridin-2(*1H*)-ylidene)propanamide*.* White powder, m. p. 152–153°C, yields 80%. ^1^H NMR (500 MHz, CDCl_3_) δ 8.57 (dd, *J* = 9.0, 1.4 Hz, 1H), 7.89–7.77 (m, 3H), 7.49–7.41 (m, 1H), 7.25–7.12 (m, 3H), 6.91 (td, *J* = 6.8, 1.5 Hz, 1H), and 5.63 (s, 2H). ^13^C NMR (126 MHz, CDCl_3_) δ 163.21 (t, *J*_*CF*_ = 24.7 Hz), 162.35, 160.37, 157.76, 141.20, 140.57, 139.05 (d, *J*_*CF*_ = 10.0 Hz), 137.47, 129.47, 129.40, 128.98, 128.68, 128.53 (q, *J*_*CF*_ = 38.4 Hz), 128.37, 128.07, 122.34, 121.41, 121.23, 120.74, 120.72, 120.20, 119.42, 119.13, 118.84, 118.05, 117.14, 117.07, 116.86, 116.57, 116.04, 115.88, 114.86, 114.58, 114.30, 113.97, 112.88, 112.68, 108.93, 108.63, 107.74 (q, *J*_*CF*_ = 36.9 Hz), 104.77, 104.47, 45.55, 45.53, 45.51, and 45.49. HRMS (ESI) *m/z*: calcd. for C_19_H_11_F_9_N_4_O [M + H]^+^, 483.0862; found, 483.0861.

Data for (***C4***) (*E*)-*N*-(1-((1-(4-chlorophenyl)-5-(trifluoromethyl)-*1H*-pyrazol-4-yl)methyl)pyridin-2(*1H*)-ylidene)-2,2,3,3,3-pentafluoropropanamide*.* White powder, 100–101°C, yields 76%. ^1^H NMR (500 MHz, CDCl_3_) δ 8.58 (dt, *J* = 8.4, 1.5 Hz, 1H), 7.86–7.77 (m, 3H), 7.46 (d, *J* = 8.7 Hz, 2H), 7.35 (d, *J* = 8.7 Hz, 2H), 6.90 (td, *J* = 6.8, 1.4 Hz, 1H), and 5.62 (s, 2H). ^13^C NMR (126 MHz, CDCl_3_) δ 163.21 (t, *J*_*CF*_ = 24.7 Hz), 158.81, 142.20, 141.59, 138.46, 137.47, 135.92, 129.44, 128.53 (q, *J*_*CF*_ = 38.4 Hz), 127.27, 123.40, 122.29, 121.25, 120.45, 120.16, 119.88, 119.10, 118.17, 118.00, 117.89, 117.60, 116.96, 114.96, 109.96, 109.66, 107.74 (q, *J*_*CF*_ = 36.9 Hz), 105.80, 105.50, and 46.57. HRMS (ESI) *m/z*: calcd. for C_19_H_11_ClF_8_N_4_O [M + H]^+^, 499.0566; found, 499.0565.

Data for (***C5***) (*E*)-2,2,3,3,3-pentafluoro-*N*-(1-((5-(trifluoromethyl)-1-(4-(trifluoromethyl)phenyl)-*1H*-pyrazol-4-yl)methyl)pyridin-2(*1H*)-ylidene)propanamide*.* Yellowish powder, m. p. 117–118 °C, yields 87%. ^1^H NMR (500 MHz, CDCl_3_) δ 8.60 (dt, *J* = 9.0, 1.0 Hz, 1H), 7.90–7.81 (m, 3H), 7.77 (d, *J* = 8.3 Hz, 2H), 7.57 (d, *J* = 8.3 Hz, 2H), 6.93 (td, *J* = 6.8, 1.5 Hz, 1H), and 5.70–5.60 (m, 2H). ^13^C NMR (126 MHz, CDCl_3_) δ 163.21 (t, *J*_*CF*_ = 24.7 Hz), 158.83, 142.36, 141.95, 141.84, 138.63, 131.85 (q, *J*_*CF*_ = 33.3 Hz), 129.61 (q, *J*_*CF*_ = 38.4 Hz), 126.54, 126.51, 126.48, 126.45, 126.35, 124.62, 123.41, 122.45, 122.28, 121.26, 120.49, 120.20, 119.92, 119.12, 118.61, 118.21, 117.93, 117.64, 116.97, 115.94, 115.65, 115.36, 115.09, 110.30, 110.00, 109.71, 109.43, 107.78 (q, *J*_*CF*_ = 36.9 Hz), 106.14, 105.85, 105.55, 105.26, and 46.69. HRMS (ESI) *m/z*: calcd. for C_20_H_11_F_11_N_4_O [M + H]^+^, 533.0830; found, 533.0831.

Data for (***C6***) (*E*)-*N*-(1-((1-(3-chlorophenyl)-5-(difluoromethyl)-*1H*-pyrazol-4-yl)methyl)pyridin-2(*1H*)-ylidene)-2,2,3,3,3-pentafluoropropanamide*.* White powder, m. p. 72–73°C, yields 75%. ^1^H NMR (500 MHz, CDCl_3_) δ 8.57 (dd, *J* = 9.2, 1.2 Hz, 1H), 8.00 (s, 1H), 7.94 (dd, *J* = 6.7, 1.9 Hz, 1H), 7.79 (ddd, *J* = 8.9, 7.0, 1.8 Hz, 1H), 7.53–7.43 (m, 3H), 7.32 (dt, *J* = 7.0, 2.0 Hz, 1H), 6.92–6.65 (m, 2H), and 5.66 (s, 2H). ^13^C NMR (126 MHz, CDCl_3_) δ 164.10 (t, *J*_*CF*_ = 24.5 Hz), 158.64, 142.40, 142.09, 139.16, 138.99, 135.44, 133.42 (t, *J*_*CF*_ = 29.7 Hz), 130.61, 129.69, 125.65, 123.27, 122.04, 120.49, 120.20, 119.92, 118.22, 117.93, 117.64, 117.15, 115.04, 110.98, 110.34, 110.04, 109.75, 109.11, 107.78 (q, *J*_*CF*_ = 37.1 Hz), 107.24, 105.88, 105.59, and 46.05. HRMS (ESI) *m/z*: calcd. for C_19_H_12_ClF_7_N_4_O [M + H]^+^, 481.0661; found, 481.0661.

Data for (***C7***) (*E*)-*N*-(1-((5-(difluoromethyl)-1-(3-fluorophenyl)-*1H*-pyrazol-4-yl)methyl)pyridin-2(*1H*)-ylidene)-2,2,3,3,3-pentafluoropropanamide*.* White powder, m. p. 81–82°C, yields 82%. ^1^H NMR (500 MHz, CDCl_3_) δ 8.57 (dd, *J* = 9.0, 1.4 Hz, 1H), 7.99 (s, 1H), 7.95 (dd, *J* = 6.8, 1.8 Hz, 1H), 7.79 (ddd, *J* = 8.9, 7.0, 1.8 Hz, 1H), 7.53–7.46 (m, 1H), 7.25–7.18 (m, 3H), 6.92–6.68 (m, 2H), and 5.66 (s, 2H). ^13^C NMR (126 MHz, CDCl_3_) δ 164.10 (t, *J*_*CF*_ = 24.5 Hz), 163.81, 161.82, 158.72, 142.43, 142.02, 139.35 (d, *J*_*CF*_ = 10.0 Hz), 138.89, 138.87, 138.85, 133.40 (t, *J*_*CF*_ = 29.8 Hz), 131.03, 130.96, 122.14, 120.79, 120.76, 120.49, 120.20, 119.92, 118.21, 117.93, 117.64, 117.12, 116.72, 116.56, 114.92, 113.16, 112.96, 111.04, 109.17, 107.78 (q, *J*_*CF*_ = 37.1 Hz), 107.30, and 46.07. HRMS (ESI) *m/z*: calcd. for C_19_H_12_F_8_N_4_O [M + H]^+^, 465.0956; found, 465.0955.

Data for (***C8***) (*E*)-*N*-(1-((5-(difluoromethyl)-1-(4-fluorophenyl)-*1H*-pyrazol-4-yl)methyl)pyridin-2(*1H*)-ylidene)-2,2,3,3,3-pentafluoropropanamide*.* Colorless oil yields 79%. ^1^H NMR (500 MHz, CDCl_3_) δ 8.56 (dd, *J* = 9.1, 1.6 Hz, 1H), 8.02–7.92 (m, 2H), 7.79 (ddt, *J* = 8.6, 6.9, 1.7 Hz, 1H), 7.41 (ddd, *J* = 8.6, 4.6, 1.6 Hz, 2H), 7.26–7.15 (m, 2H), 6.88 (td, *J* = 6.8, 1.5 Hz, 1H), 6.73 (t, *J* = 52.7 Hz, 1H), and 5.65 (s, 2H). ^13^C NMR (126 MHz, CDCl_3_) δ 164.22 (t, *J*_*CF*_ = 24.5 Hz), 163.88, 161.89, 158.74, 142.14, 141.97, 138.84, 134.28 (d, *J*_*CF*_ = 3.3 Hz), 133.49 (t, *J*_*CF*_ = 29.6 Hz), 127.39, 127.32, 122.16, 120.21, 119.92, 117.93, 117.64, 116.79, 116.66, 116.61, 114.87, 111.13, 109.26, 107.95, 107.65, 107.39, and 46.07. HRMS (ESI) *m/z*: calcd. for C_19_H_12_F_8_N_4_O [M + H]^+^, 465.0956; found, 465.0956.

Data for (***D1***) (*E*)-3-(difluoromethyl)-1-methyl-*N*-(1-((1-phenyl-5-(trifluoromethyl)-*1H*-pyrazol-4-yl)methyl)pyridin-2(*1H*)-ylidene)-*1H*-pyrazole-4-carboxamide*.* White powder, m. p. 169–170 °C, yields 64%. ^1^H NMR (500 MHz, DMSO-*d*_*6*_) δ 8.36 (dd, *J* = 9.2, 1.4 Hz, 1H), 8.23–8.16 (m, 2H), 7.83 (ddd, *J* = 9.0, 6.8, 1.8 Hz, 1H), 7.65–7.59 (m, 3H), 7.57 (s, 1H), 7.51 (dd, *J* = 6.5, 3.1 Hz, 2H), 7.36 (t, *J* = 54.5 Hz, 1H), 6.86 (td, *J* = 6.8, 1.5 Hz, 1H), 5.69 (s, 2H), and 3.92 (s, 3H). ^13^C NMR (126 MHz, DMSO-*d*_*6*_) δ 168.92, 158.39, 144.31 (t, *J*_*CF*_ = 22.2 Hz), 141.24, 140.50, 140.40, 139.22, 135.37, 135.34, 130.40, 129.82, 128.27 (q, *J*_*CF*_ = 38.3 Hz), 126.54, 123.81, 123.13, 123.09, 123.06, 121.67, 120.47, 120.23, 119.52, 117.37, 112.44, 112.26, 110.40, 108.54, 46.44, and 39.57. HRMS (ESI) *m/z*: calcd. for C_22_H_17_F_5_N_6_O [M + H]^+^, 477.1457; found, 477.1457.

Data for (***D2***) (*E*)-*N*-(1-((1-(2-chlorophenyl)-5-(trifluoromethyl)-*1H*-pyrazol-4-yl)methyl)pyridin-2(*1H*)-ylidene)-3-(difluoromethyl)-1-methyl-*1H*-pyrazole-4-carboxamide*.* White powder, m. p. 158–159°C, yields 56%. ^1^H NMR (500 MHz, CDCl_3_) δ 8.45 (dd, *J* = 9.1, 1.4 Hz, 1H), 7.87 (s, 1H), 7.67–7.32 (m, 8H), 6.61 (td, *J* = 6.7, 1.5 Hz, 1H), 5.68–5.48 (m, 2H), and 3.91 (s, 3H). ^13^C NMR (126 MHz, CDCl_3_) δ 169.53, 158.61, 145.40 (t, *J*_*CF*_ = 22.7 Hz), 140.57, 139.96, 137.98, 136.31, 134.31, 132.75, 131.73, 130.30 (q, *J*_*CF*_ = 38.3 Hz), 130.22, 129.65, 129.42, 127.46, 123.15, 123.05, 123.02, 122.98, 121.51, 121.00, 118.85, 118.28, 116.70, 111.87, 111.83, 110.00, 108.12, 46.03, and 39.41. HRMS (ESI) *m/z*: calcd. for C_22_H_16_ClF_5_N_6_O [M + H]^+^, 511.1067; found, 511.1068.

Data for (***D3***) (*E*)-3-(difluoromethyl)-*N*-(1-((1-(3-fluorophenyl)-5-(trifluoromethyl)-*1H*-pyrazol-4-yl)methyl)pyridin-2(*1H*)-ylidene)-1-methyl-1H-pyrazole-4-carboxamide*.* White powder, m. p. 140–141°C, yields 58%. ^1^H NMR (500 MHz, DMSO-*d*_*6*_) δ 8.32 (dd, *J* = 9.1, 1.4 Hz, 1H), 8.20–8.13 (m, 2H), 7.80 (ddd, *J* = 8.9, 6.8, 1.8 Hz, 1H), 7.64 (td, *J* = 8.2, 6.3 Hz, 1H), 7.59 (s, 1H), 7.50–7.21 (m, 4H), 6.83 (td, *J* = 6.7, 1.5 Hz, 1H), 5.66 (s, 2H), and 3.90 (s, 3H). ^13^C NMR (126 MHz, DMSO-*d*_*6*_) δ 168.91, 163.13, 161.17, 158.35, 144.30 (t, *J*_*CF*_ = 22.3 Hz), 141.19, 140.86, 140.43, 140.31 (d, *J*_*CF*_ = 10.2 Hz), 135.34, 131.65, 131.58, 128.38 (q, *J*_*CF*_ = 38.3 Hz), 123.67, 123.07, 123.04, 123.01, 122.86, 121.52, 120.50, 120.43, 119.38, 117.60, 117.43, 117.23, 114.30, 114.10, 112.37, 112.25, 110.38, 108.52, 46.29, 46.26, and 39.54. HRMS (ESI) *m/z*: calcd. for C_22_H_16_F_6_N_6_O [M + H]^+^, 495.1363; found, 495.1360.

Data for (***D4***) (*E*)-*N*-(1-((1-(4-chlorophenyl)-5-(trifluoromethyl)-*1H*-pyrazol-4-yl)methyl)pyridin-2(*1H*)-ylidene)-3-(difluoromethyl)-1-methyl-*1H*-pyrazole-4-carboxamide*.* White powder, m. p. 75–76°C, yields 57%. ^1^H NMR (500 MHz, DMSO-*d*_*6*_) δ 8.32 (dd, *J* = 9.2, 1.4 Hz, 1H), 8.18–8.13 (m, 2H), 7.80 (ddd, *J* = 9.0, 6.8, 1.8 Hz, 1H), 7.66 (d, *J* = 8.7 Hz, 2H), 7.57 (s, 1H), 7.53 (d, *J* = 8.7 Hz, 2H), 7.31 (t, *J* = 54.5 Hz, 1H), 6.83 (td, *J* = 6.7, 1.5 Hz, 1H), 5.65 (s, 2H), and 3.89 (s, 3H). ^13^C NMR (126 MHz, DMSO-*d*_*6*_) δ 168.88, 158.34, 144.26 (t, *J*_*CF*_ = 22.0 Hz), 141.22, 140.78, 140.46, 137.95, 135.33, 135.03, 129.92, 128.35, 128.34 (q, *J*_*CF*_ = 38.0 Hz), 123.69, 123.07, 123.03, 123.00, 121.54, 120.47, 120.42, 119.40, 117.25, 112.40, 112.23, 110.36, 108.50, 46.32, and 39.55. HRMS (ESI) *m/z*: calcd. for C_22_H_16_ClF_5_N_6_O [M + H]^+^, 511.1067; found, 511.1068.

Data for (***D5***) (*E*)-3-(difluoromethyl)-1-methyl-*N*-(1-((5-(trifluoromethyl)-1-(4-(trifluoromethyl)phenyl)-*1H*-pyrazol-4-yl)methyl)pyridin-2(*1H*)-ylidene)-*1H*-pyrazole-4-carboxamide*.* White powder, m. p. 139–140 °C, yields 65%. ^1^H NMR (500 MHz, CDCl_3_) δ 8.43 (d, *J* = 9.0 Hz, 1H), 7.88 (s, 1H), 7.76 (d, *J* = 8.3 Hz, 2H), 7.65–7.55 (m, 5H), 7.27 (t, 1H), 6.62 (td, *J* = 6.8, 1.4 Hz, 1H), 5.57 (s, 2H), and 3.93 (s, 3H). ^13^C NMR (126 MHz, CDCl_3_) δ 169.57, 158.77, 145.29 (t, *J*_*CF*_ = 22.9 Hz), 141.80, 141.09, 139.90, 137.76, 134.41, 132.16, 131.76 (q, *J*_*CF*_ = 33.2 Hz), 129.27 (q, *J*_*CF*_ = 38.5 Hz), 126.48, 126.45, 126.42, 126.39, 126.32, 124.57, 123.39, 122.98, 122.40, 121.91, 121.24, 119.46, 119.09, 116.94, 111.88, 111.80, 110.00, 108.13, 46.11, and 39.51. HRMS (ESI) *m/z*: calcd. for C_23_H_16_F_8_N_6_O [M + H]^+^, 545.1331; found, 545.1328.

Data for (***D6***) (*E*)-*N*-(1-((1-(3-chlorophenyl)-5-(difluoromethyl)-*1H*-pyrazol-4-yl)methyl)pyridin-2(*1H*)-ylidene)-3-(difluoromethyl)-1-methyl-*1H*-pyrazole-4-carboxamide*.* White powder, m. p. 126–127°C, yields 55%. ^1^H NMR (500 MHz, DMSO-*d*_*6*_) δ 8.30 (d, *J* = 13.3 Hz, 2H), 8.20 (d, *J* = 6.8 Hz, 1H), 7.80 (s, 1H), 7.73 (t, *J* = 8.1 Hz, 1H), 7.62–7.33 (m, 6H), 6.79 (t, *J* = 6.7 Hz, 1H), 5.66 (s, 2H), and 3.91 (s, 3H). ^13^C NMR (126 MHz, DMSO-*d*_*6*_) δ 169.05, 158.16, 144.69 (t, *J*_*CF*_ = 22.3 Hz), 141.59, 140.92, 140.50, 140.27, 135.26, 134.00, 133.28 (t, *J*_*CF*_ = 26.8 Hz), 131.43, 129.53, 125.64, 124.39, 123.07, 123.04, 123.01, 120.36, 119.56, 119.54, 119.52, 112.33, 112.31, 111.14, 110.47, 109.28, 108.61, 107.42, 45.41, and 39.53. HRMS (ESI) *m/z*: calcd. for C_22_H_17_ClF_4_N_6_O [M + H]^+^, 493.1161; found, 493.1159.

Data for (***D7***) (*E*)-3-(difluoromethyl)-*N*-(1-((5-(difluoromethyl)-1-(4-fluorophenyl)-*1H*-pyrazol-4-yl)methyl)pyridin-2(*1H*)-ylidene)-1-methyl-*1H*-pyrazole-4-carboxamide*.* White powder, m. p. 185–186°C, yields 51%. ^1^H NMR (500 MHz, DMSO-*d*_*6*_) δ 8.32–8.26 (m, 2H), 8.20 (dd, *J* = 6.8, 1.8 Hz, 1H), 7.78–7.73 (m, 2H), 7.57–7.28 (m, 6H), 6.80 (td, *J* = 6.7, 1.5 Hz, 1H), 5.64 (s, 2H), and 3.91 (s, 3H). ^13^C NMR (126 MHz, DMSO-*d*_*6*_) δ 169.00, 163.37, 161.41, 158.14, 144.64 (t, *J*_*CF*_ = 22.3 Hz), 141.15, 140.97, 140.30, 135.79 (d, *J*_*CF*_ = 2.9 Hz), 135.25, 133.33 (t, *J*_*CF*_ = 26.6 Hz), 128.22, 128.14, 123.04, 123.01, 122.98, 120.34, 119.07, 119.04, 119.02, 116.78, 116.59, 112.36, 112.30, 111.13, 110.44, 109.26, 108.57, 107.40, 45.35, and 39.54. HRMS (ESI) *m/z*: calcd. for C_22_H_17_F_5_N_6_O [M + H]^+^, 477.1457; found, 477.1456.

### X-ray diffraction

Compound **A3** with high purity was dissolved in ethyl acetate/petroleum ether (v/v = 1: 5), and the solution was placed in a glass bottle with a lid and stood on a flat table. With the slow and natural volatilization of the solvent, the crystal grew slowly. After 3 days, a single crystal meeting the requirements of crystal diffraction was obtained.

The crystal size and strength were measured by Agilent Gemini E double-source X-ray single crystal diffractometer. **A3** crystal was installed in inert oil at room temperature and transferred to a diffractometer with cold airflow. With Mo Kα ray as the radiation source and graphite monochromator, data were collected by the Xscan program, and 7,347 diffraction points were collected in the range of 6.58 < 2θ < 51.98 (-13 ≤ H ≤ 13, -16 ≤ k ≤11, -15 ≤ l ≤ 11) by ω/2θ scanning mode, respectively. The strength data were corrected by LP and empirical absorption, and the structure was solved by a direct method by using the SHELXTL-97 program on a microcomputer. The structure was refined by using the whole matrix least square correction program.

### Insecticidal activity assay

*Preparation of test agent*. Dissolve accurately weighed tested compound in DMF to prepare 1% mother liquor, and then dilute it with distilled water containing 0.1% Tween-80 to corresponding concentrations for later use.

Insecticidal activity against *Helicoverpa armigera*. The soaking method was adopted (Yang et al., 2022). After soaking a proper amount of corn leaves for 10 s, they were placed in a plastic Petri dish with filter paper and dried naturally in the shade. Ten larvae of the 2nd instar *H. armigera* were attached to each dish and placed in an observation room at 26°C with light (16/8 h). The result was counted 2 days later, and the no-response insect body when it was lightly touched with a brush was regarded as dead. Each treatment was repeated 3 times, and DMF without the tested compound was used as a blank control.

Insecticidal activity against *Chilo suppressalis* (Huang et al., 2022)*.* The soaking method was adopted. The *Zizania latifolia* slices were soaked in a tested solution for 10 s, then taken out, and placed in a plastic Petri dish with filter paper to naturally dry in the shade. Ten larvae of the 3rd instar *C. suppressalis* were attached to each dish, and the room was observed at 26°C under light (16/8 h). The result was counted 4 days later, and the no-response insect body when it was lightly touched with a brush was regarded as dead. Each treatment was repeated three times, and DMF without the tested compound was used as a blank control.

Insecticidal activity against *Nilaparvata lugens* (Ghareeb et al., 2021)*.* The seedlings with two leaves and one heart were placed in a Petri dish with a bottom diameter of 3 cm and covered with white sand. After the 3rd instar nymphae of *N. lugens* were stunned by CO_2_, about 15 nymphae were received in each dish, and then 2.5 ml of the tested agent was sprayed with Potter spray tower. The mortality of *N. lugens* was investigated 1 day after the treatment. Each treatment was repeated three times, and DMF without the tested compound was used as a blank control.

Insecticidal activity against *Plutella xylostella* (Yang et al., 2021)*.* The soaking method was adopted. After soaking a proper amount of radish leaves for 10 s, it was placed in a plastic Petri dish with filter paper and dried naturally in the shade. Each dish was inoculated with 10 2nd instar *P. xylostella* larvae and placed in an observation room at 22°C with light (16/8 h). The result was counted 2 days later, and the no-response insect body when it was lightly touched with a brush was regarded as dead. Each treatment was repeated three times, and DMF without the tested compound was used as a blank control.

Insecticidal activity against *Aphis craccivora* (Liu et al., 2022)*.* About 30 nymphs of alfalfa aphid were placed on a horsebean leaf dish, and then 2.5 ml of the tested agent was sprayed with Potter spray tower. After treatment, they were cultured in an observation room at 20–22°C. The result was counted 24 h later, and the no-response insect body when it was lightly touched with tweezers was regarded as dead. Each treatment was repeated three times, and DMF without the tested compound was used as a blank control.

The insecticidal activities of the test compounds against the abovementioned five insects were calculated by the following formula:

$$P_{1}=\left(K/N\right)\times 100,$$ (1)

$$P_{2}=\left(P_{t}-P_{0}\right)/\left(1-P_{0}\right)\times 100,$$ (2)

where P_1_ is the mortality rate; K is the dead insect number; N is the total number of experimental insects; P_2_ is the corrected mortality rate; P_t_ is the mortality rate of treatment; P_0_ is the mortality rate of CK. If the mortality rate of CK is less than 5%, without the required correction, and if the mortality rate of CK is between 5 and 20%, P_2_ should be calculated according to formula II.

### Bee toxicity evaluation assay

The test bees were Italian bees (*Apis mellifera*), and the newly born adult worker bees (<24 h) were selected for drug drip treatment. Weigh 50 mg of test substance **FLP** and fully dissolve it in DMSO to obtain 10 × 10^4^ mg A.I./L of stock liquid medicine; dissolve **B4** fully in DMSO and fix the volume to a 1-ml volumetric flask to obtain 11.6 × 10^4^ mg A.I./L stock solution.

Contact toxicity. 10 × 10^4^ mg A.I./L of mother liquor was used as the test liquid, and 1 μL of the solution containing the tested drug was dripped onto the back of the front chest of bees by a micro-dropper. Then, bees were put into a beekeeping box and cultured in dark conditions at a temperature of 30 ± 1°C and relative humidity of 50%, with a 50% sucrose solution as food for 48 h. Taking the solvent drip bees without the drug to be tested as the blank control, the treatment group and the control group were set up with three replicates, and 10 bees were used in each replicate.

Oral toxicity. Before the formal experiment, a pre-experiment was carried out. According to the formal experimental conditions, three to four dose groups were set at large intervals to observe the death of bees between 24 and 48 h and determine the linear range of the lethal effect of drugs on bees. According to the pre-test results, determine the concentration range of the formal test and set five to seven test dose groups. Starve bees for 2 h before being infected, and then eliminate bees that are dying or have abnormal behaviors; put 200 μL of test liquid medicine in the feeding tube, and after the liquid medicine is consumed (within 6 h), take out the feeder to measure the weight of the food that has not been consumed. Then, the bees were fed with sucrose solution without test samples. Cultivate in the dark. At the same time, feeding 50% sucrose solution as a blank control, the treatment group and the control group were set up with three replicates, and 20 bees were used in each replicate. According to the results of the pre-experiment, if the toxicity of the drug to bees is low, the upper limit dose of 100 μg a.i./bee is set for the limit test of bees, that is, when the tested substance reaches 100 μg a.i./bee, no bees die, so there is no need to continue the test. If the solubility of the test substance is less than 100 μg a. i./bee, the maximum solubility is adopted as the upper limit concentration. The test results were examined at 24 and 48 h, respectively, and the number of deaths and poisoning symptoms were recorded. The standard of death is touching the bee's body, and when it can't crawl, it is judged as death.

Statistical analysis. Using the statistical software SPSS16.0, the LD_50_ value and 95% confidence limit of the half lethal dose of the sample to bees were obtained by regression statistical analysis with the probit analysis method.

### Density functional theory (DFT-B3LYP) calculation

Flupyrimin and the most effective compound **B4** were selected for DFT calculations. They were drawn in OpenBable (http://www.cheminfo.org/Chemistry/Cheminformatics/FormatConverter/index.html) and then optimized using DFT/B3LYP combined with standard 6-31G (d,p) methods in the Gaussian 09 W package. The highest occupied molecular orbital (HOMO), lowest unoccupied molecular orbital (LUMO), and the electrostatic potential (ESP) were carried out using the DFT-B3LYP/6-31G (d,p) basis set, combined with the Multiwfn software package (Lu and Chen, 2012) and VMD program (Humphrey et al., 1996). The range of colors on the surface of the ESP is fading from red (most negative) to blue (most positive).

### Docking simulation

As everyone knows, the nAChR is a potential target for neonicotinoid insecticides. So, structural models for flupyrimin, **A3**, **B4**, and **D6** are based on the crystal structure of the imidacloprid-AChBP (*Aplysia*) complex (Protein Data Bank ID code 3c79). Imidacloprid was removed, and then the four selected compounds were individually redocked. Docking calculations were carried out by using the Sybyl x2.0 (Norel et al., 1995) and AutoDock Vina programs (Harris et al., 2008; Feinstein and Brylinski, 2015; Eberhardt et al., 2021). The PyMoL software (Lill and Danielson, 2011) was used to visualize and analyze the resultant model structures, as well as to generate graphical presentations and illustrative figures.

## Results and discussion

### Chemistry

**SCHEME 1:**
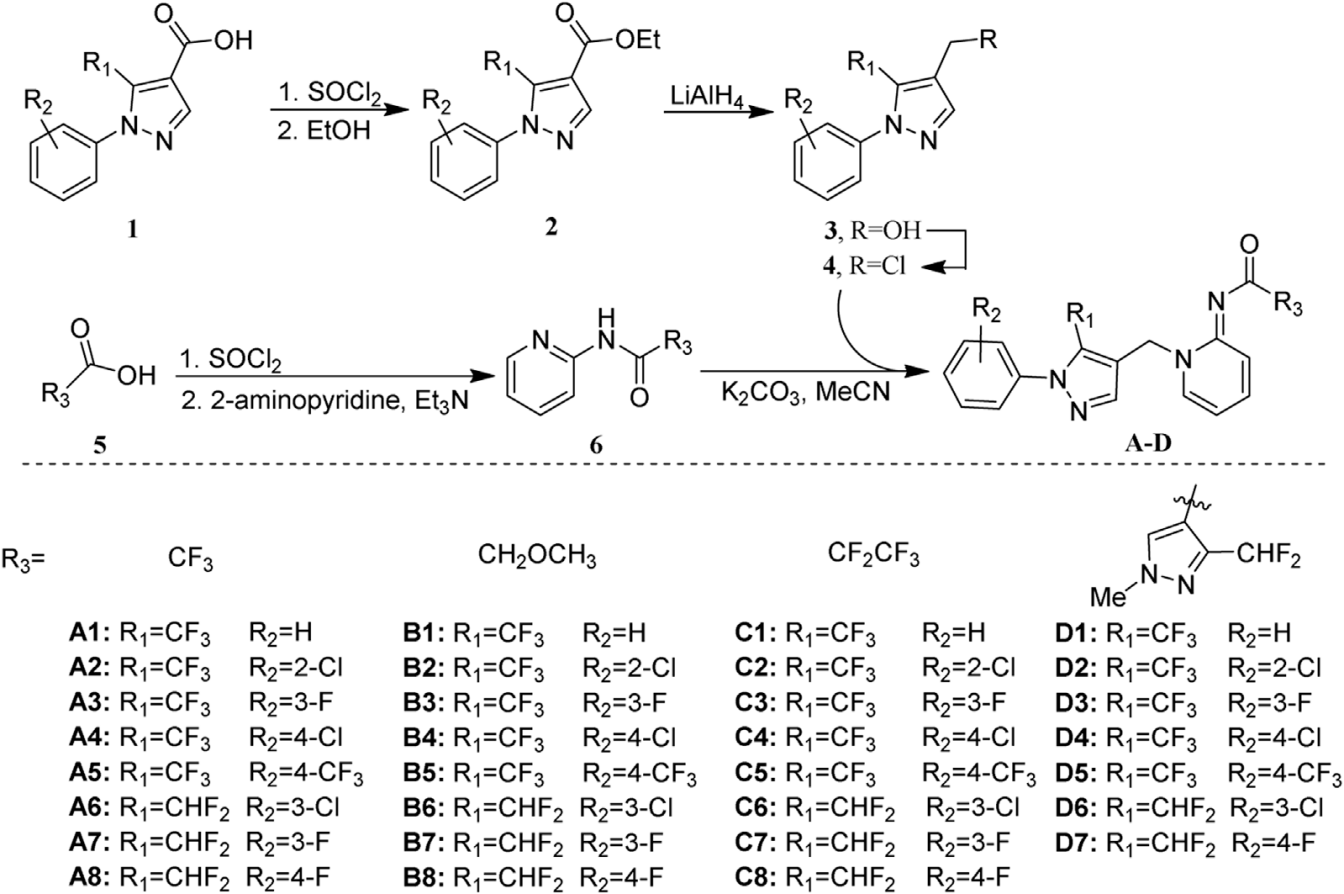
Synthetic pathway of the target compounds **A–D**.

The general synthetic pathway of target compounds was illustrated in Scheme 1. The key intermediates **4–1** ∼ **4–8** were synthesized with satisfactory yield through acylation, esterification, reduction, and chlorination, subsequently starting from 1-aryl-*1H*-pyrazole-4-carboxylic acid. Another key intermediate N-(pyridine-2-yl)amide **6–1** ∼ **6–4** was prepared in high yields *via* acylation of 2-aminopyridine conveniently. The target compounds **A–D** could be obtained by the coupling of **4** and **6** in the presence of K_2_CO_3_ and a catalytic amount of KI.

### Crystal structure analysis

**FIGURE 3:**
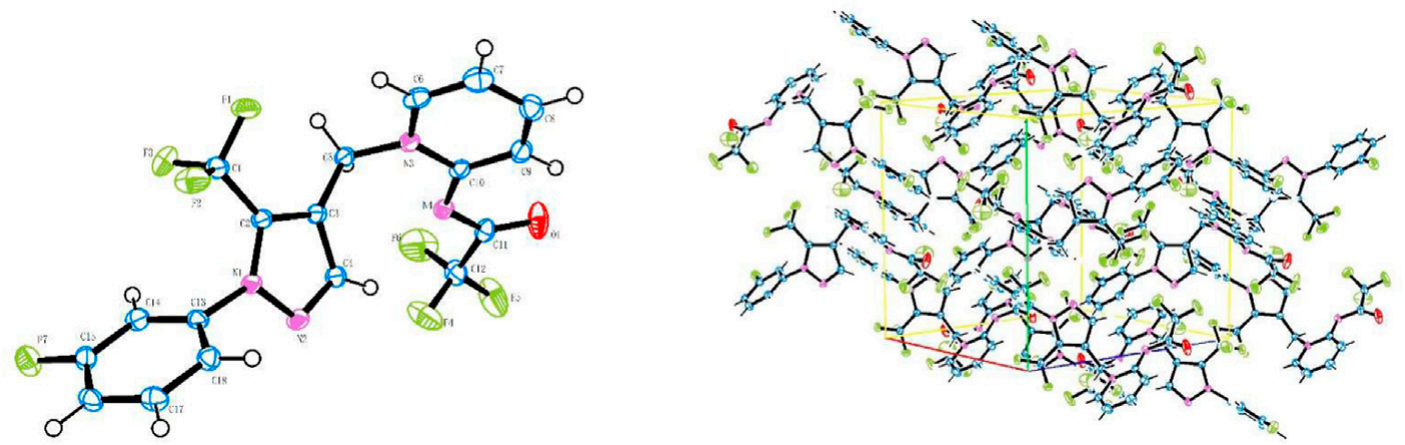
Crystal structure of compound **A3** and the packing representation (CCDC number 2106763).

The molecular structure of compound **A3** is shown in Figure 3 and its structural parameters are summarized in Table 1. The C (10) = N(M) bond length is a typical exocyclic C = N bond (1.345 Å), indicating that the pyridine ring of compound **A3** is dearomatized and its double bond is in the trans configuration. It is in consistence with the structure of flupyrimin.

**TABLE 1:** Structural parameters of compound **A3**.

Items	Data
Empirical formula	C_18_H_11_F_7_N_4_O
Formula weight	432.31
Temperature/K	113.20 (10)
Crystal system	Monoclinic
Space group	P2_1_/c
a/Å, b/Å, and c/Å	10.7864(6), 13.1528(8), and 12.9293(8)
α/°, β/°, and γ/°	90.00, 106.573 (6), and 90.00
Volume/Å^3^	1758.10 (18)
Z	4
ρ_calc_/mg mm^−3^	1.633
μ/mm^−1^	0.156
F(ooo)	872
Crystal size/mm^3^	0.24 × 0.22 × 0.13
2Θ range for data collection	6.58–51.98°
Index ranges	-13 ≤ h ≤ 13, -16 ≤ k ≤ 11, and -15 ≤ l ≤ 11
Reflections collected	7,347
Independent reflections	3,368 [R (int) = 0.0236 (inf-0.9Å)]
Data/restraints/parameters	3,368/0/275
Goodness-of-fit on F^2^	1.018
Final R indexes [I > 2σ (I) i.e., F_o_ > 4σ (F_o_)]	R_1_ = 0.0453 and wR_2_ = 0.0957
Final R indexes [all data]	R_1_ = 0.0576 and wR_2_ = 0.1035
Largest diff. peak/hole/e Å^−3^	0.346/-0.339
Flack parameters	N
Completeness	0.9970

### Insecticidal activity

The insecticidal activities are summarized in Table 2. All the synthesized compounds exhibited no insecticidal activities against *Helicoverpa armigera*, and the control agent flupyrimin was also inactive at 400 μg/ml. However, the lethality of the control agent chlorantraniliprole at this concentration reached 100% for *Helicoverpa armigera*. Most compounds showed no insecticidal activity against the *Chilo suppressalis*, but fortunately, compounds **C5** and **C7** displayed lethality rates of 100 and 40%, respectively. We speculated that the pentafluoroacetyl moieties as well as the benzene ring of the aryl pyrazole moiety containing fluorine atom substitutions of **C5** and **C7** may be an important reason why they exhibited some activities for *Chilo suppressalis*. Compounds **A2**, **A6**, **C5**, **D3**, **D6**, and **D7** led to a 100% mortality rate for *Nilaparvata lugens* at 400 μg/ml, which is equivalent to the insecticidal activity of the control Flupyrimin and better activity than the control chlorantraniliprole.

**TABLE 2:** Insecticidal activities of the target compounds at 400 μg/ml.

Compound	Log P	Mortality rate (%)									
		*A. craccivora*		*N. lugens*		*P. xylostella*			*C. suppressalis*		*H. armigera*
		400	100	400	100	400	100	25	400	100	400
		μg/mL	μg/mL	μg/mL	μg/mL	μg/mL	μg/mL	μg/mL	μg/mL	μg/mL	μg/mL
**A1**	4.65	0	—	0	—	76.7	—	—	0	—	0
**A2**	5.21	100	0	100	0	36.7	—	—	0	—	0
**A3**	4.81	0	—	0	—	100	86.7	0	0	—	0
**A4**	5.21	0	—	0	—	0	—	—	0	—	0
**A5**	5.57	0	—	0	—	73.3	—	—	0	—	0
**A6**	4.55	0	—	100	0	50.0	—	—	0	—	0
**A7**	4.15	0	—	0	—	63.3	—	—	0	—	0
**A8**	4.15	0	—	0	—	0	—	—	0	—	0
**B1**	3.17	0	—	48.9	—	100	100	16.7	0	—	0
**B2**	3.72	0	—	0	—	100	96.7	73.3	0	—	0
**B3**	3.32	0	—	0	—	100	100	76.7	0	—	0
**B4**	3.72	0	—	0	—	100	96.7	100	0	—	0
**B5**	4.09	0	—	0	—	100	100	0	0	—	0
**B6**	3.07	0	—	0	—	100	83.3	0	0	—	0
**B7**	2.67	0	—	0	—	66.7	—	—	0	—	0
**B8**	2.67	0	—	0	—	56.7	—	—	0	—	0
**C1**	5.25	0	—	0	—	0	—	—	0	—	0
**C2**	5.81	0	—	0	—	0	—	—	0	—	0
**C3**	5.41	0	—	0	—	76.7	—	—	0	—	0
**C4**	5.81	0	—	0	—	73.3	—	—	0	—	0
**C5**	6.17	0	—	0	—	70.0	—	—	100	0	0
**C6**	5.16	0	—	0	—	60.0	—	—	0	—	0
**C7**	4.76	0	—	0	—	66.7	—	—	40.0	—	0
**C8**	4.76	0	—	0	—	0	—	—	0	—	0
**D1**	4.43	0	—	0	—	46.7	—	—	0	—	0
**D2**	4.99	0	—	0	—	43.3	—	—	0	—	0
**D3**	4.59	0	—	100	0	63.3	—	—	0	—	0
**D4**	4.99	0	—	0	—	100	60.0	6.7	0	—	0
**D5**	5.35	0	—	0	—	63.3	—	—	0	—	0
**D6**	4.33	0	—	100	0	100	100	56.7	0	—	0
**D7**	3.93	0	—	100	0	73.3	—	—	0	—	0
**FLP**	3.24	100	100	100	100	100	0	0	100	—	0
**CAT**	4.26	0	—	0	—	100	100	100	100	100	100

Note: FLP, flupyrimin; CAT, chlorantraniliprole.

Overall, the target compounds showed excellent insecticidal activities against *Plutella xylostella*, with 11 compounds (**A3**, **B1–B6**, **D4**, and **D6**) retaining the lethality rates of 100%. The structure and activity relationship of the title compounds against *Plutella xylostella* is summarized as follows: in addition to **A8**, when R_3_ was trifluoroacetyl (**A1-A7**), the compounds containing fluorine (**A3**, **A5**, and **A7**) or hydrogen (**A1**) atoms in the substituent R_2_ had higher insecticidal activities, with the mortality rates varying from 63.3 to 100%. Keeping the substituent R_3_ unchanged, the replacement of R_2_ by other halogen atoms would lead to a significant reduction in insecticidal activities. For the compounds **B1–B6** with R_3_ being methoxymethyl, these compounds had the highest insecticidal activities, among which six compounds showed 100% lethality. It was worth noting that even if R_2_ was replaced by fluorine or other halogen atoms, there was still no obvious effect on the insecticidal activity for **B1–B6**. But when R_1_ was replaced by difluoromethyl, the insecticidal activities were significantly lower than those of trifluoromethyl. After R_3_ was substituted by a pentafluoropropionyl group, the overall insecticidal activities of the obtained compounds **C1–C8** were significantly lower than those of compounds whose R_3_ was a trifluoroacetyl moiety. In this case, when R_2_ was substituted by a 4-Cl or 3-Cl moiety, the mortality rates of **C4** and **C6** reached 73.3 and 60%, respectively, which were both higher than those of compounds **A4** and **A6** whose R_3_ was a trifluoroacetyl group. For the compounds **D1–D7** in which R_3_ was pyrazolyl moiety, the overall insecticidal activities of these compounds were excellent, with lethality rates greater than 40%. Contrary to the compounds whose R_3_ was trifluoroacetyl group, when R_2_ was substituted by chlorine atom (**C8** and **D6**), the lethality of *Plutella xylostella* was 100%, while the insecticidal activities of the compound substituted by fluorine atom were relatively low. Among all compounds, the insecticidal activities of compounds whose R_1_ was a difluoromethyl group (**A7**, **B7**, and **C7**) were lower than those of compounds whose R_1_ was a trifluoromethyl group (**A3**, **B3**, and **C3**).

Further activity screening was carried out by selecting highly active compounds at a concentration of 400 μg/mL. As shown in Table 2, unfortunately, some compounds with 100% lethality to the *Aphis craccivora*, *Nilaparvata lugens*, and *Chilo suppressalis* at 400 μg/ml showed no activity at 100 μg/ml. Obviously, the tested compounds maintained high insecticidal activities against *Plutella xylostella* at 100 μg/ml, with eight compounds showing lethality rates of over 80%. Even if the concentration was reduced to 25 μg/ml, the lethality rates of **B2**, **B3**, and **B4** to *Plutella xylostella* were still greater than 70%, while the control flupyrimin had no insecticidal activity. In conclusion, the results of re-screening showed that when R_1_ was substituted with trifluoromethyl moiety, the insecticidal activities were significantly higher than those when substituted with a difluoromethyl group, and as well as when R_3_ was a methoxymethyl group, these compounds showed the best insecticidal activities against *Plutella xylostella*.

To sum up, except **A2** exhibited a certain lethality to *Aphis craccivora* and some compounds showed partial insecticidal activities against *Nilaparvata lugens*, most of the compounds had poor activities against these two sucking pests at 400 μg/ml, indicating that they were less active at the neonicotinoid receptor; meantime, their insecticidal activities higher than those of the chlorantraniliprole implied that they still retained certain neonicotinoid receptor activity. More importantly, the insecticidal activities of **B4** and chlorantraniliprole to *Plutella xylostella* were almost parallel, which indicated that **B4** had the potential to be developed as a special insecticide for *Plutella xylostella*, which was of great significance for solving the current increasingly urgent problem of *Plutella xylostella* resistance.

### DFT (B3LYP) calculation

**FIGURE 4:**
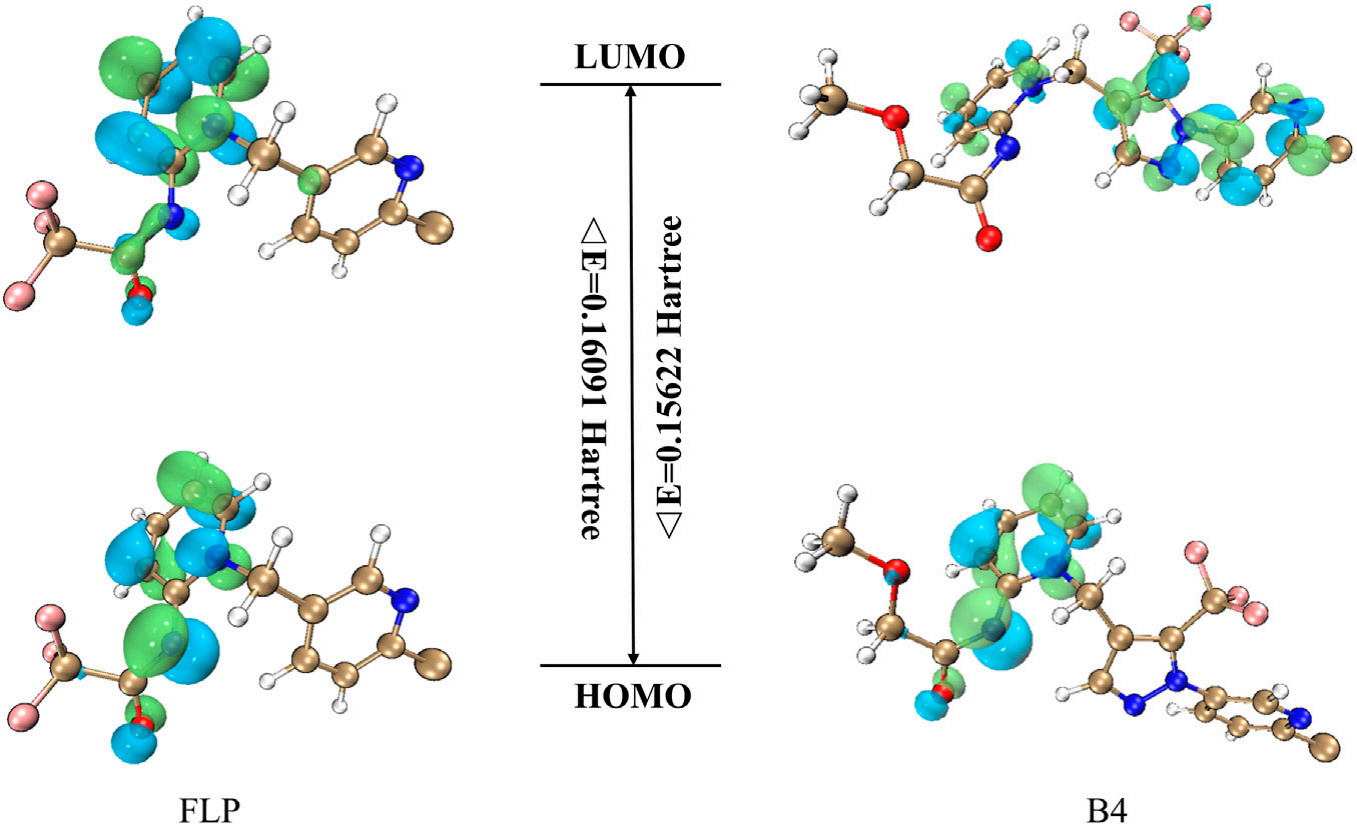
Frontier molecular orbitals of **FLP** (flupyrimin) and **B4**.

To study which properties intrinsic to the molecule will affect the **A–D** insecticidal activity against the *Plutella xylostella*, we, therefore, performed DFT calculations for the **FLP** and **B4** based on the B3LYP/6-31G (d,p) method to see if useful information could be observed. According to the frontier molecular orbital theory, the HOMO and LUMO are the two most important factors that influence the bioactivities of compounds. The HOMO energy is associated with the ionization potential, and the LUMO energy is related to electron affinity (Jiang et al., 2015). The higher HOMO energy tends to preferentially donate electrons in the first place, and the lower LUMO energy indicates that the molecule has the priority to accept electrons. Moreover, a lower energy gap (ΔE) between HOMO and LUMO usually means that the molecule has better insecticidal or fungicidal activity (Jiang et al., 2015). As shown in Figure 4, the positive molecular orbitals were symbolized with blue, and the negative molecular orbitals were symbolized with green for both the HOMO and LUMO. From Table 3, it can be concluded that the compound **B4** had lower ΔE (0.15622 Hartree) than the control **FLP** (0.16091 Hartree), indicating that **B4** had better insecticidal activity, which was also very consistent with the insecticidal data. At the same time, the frontier molecular orbitals are located in the main groups, in which atoms can easily bind to the receptor. Analyzing the LUMO and HOMO maps of the **FLP** and **B4** illustrates that their HOMO orbital distributions are similar, which are mainly located on the dearomatized pyridine ring, the outer double bond, and the carbonyl group. However, their LUMO orbital cloud density distributions are quite different, which is likely to be a possible reason leading to their different bioactivity behavior. When the electron transition takes place, the LUMO orbital cloud density of the **FLP** is still mainly concentrated in the dearomatized pyridine ring; while the **B4** electron transition takes place from the dearomatized pyridine ring to the arypyrazole group, we introduce an active skeleton. The results suggest that when compound **B4** binds to the receptor, the dearomatized pyridine ring, the outer double bond, and the carbonyl group deliver electrons, whereas the arypyrazole group receives electrons.

**TABLE 3:** Total energy and frontier orbital energy.

Energy	FLP	B4
E_Total_/Hartree	−1499.98765	−1878.81944
E_HOMO_/Hartree	−0.23416	−0.21187
E_LUMO_/Hartree	−0.07325	−0.05565
ΔE/Hartree	0.16091	0.15622
TPSA	47.26	61.43
CLogP	2.572	4.103

Note: ΔE = E_LUMO_–E_HOMO_.

**FIGURE 5:**
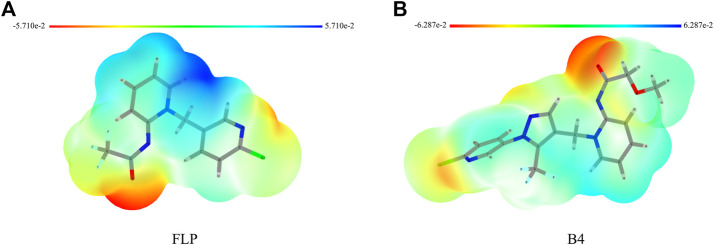
ESP of **FLP (A)** and **B4 (B)**.

Knowing the molecular electrostatic potential, especially the ESP surface, can help us check the interactions between receptors and small molecules. Hence, we selected the **FLP** and the highly active compound **B4** for ESP analysis. As shown in Figure 5, the surface of the electrostatic distribution of the **B4** is different from that of the **FLP**. The positive regions (blue) of **FLP** are mainly around the dearomatized pyridine ring compared with those of the **B4,** which are relatively scattered, including the arylpyrazole and dearomatized pyridine rings. The negative regions (red) are concentrated on the carbonyl oxygen atom, indicating that it may play an important role in its binding to the target site receptor.

Physiochemical properties of molecules play a key role in agrochemical bio-behaviors. For the compounds **FLP** and **B4**, we predicted the topological polar surface areas (TPSAs) from the website (http://www.molinspiration.com/cgi-bin/properties) and the octanol-water partition coefficients (ClogP) by ChemDraw software. According to statistics, the ClogP values of most commercial pesticides are close to 4.00 ± 2.30 (Hao et al., 2011). As shown in Table 3, the ClogP value of the control **FLP** is 2.572, which is lower than that of commercial insecticides. The ClogP value of optimized candidate compound **B4** is 4.103, which is close to that of commercial insecticides. It can be seen from Table 3 that the most suitable TPSA value for these kinds of compounds is about 50, and the TPSA value of **B4** is 61.43, which is much higher. The results showed that we perhaps need to maintain its ClogP value at around 2.572 and reduce its TPSA value to near 50 to improve the insecticidal activity of designed compounds in the future.

### Docking simulation

**FIGURE 6:**
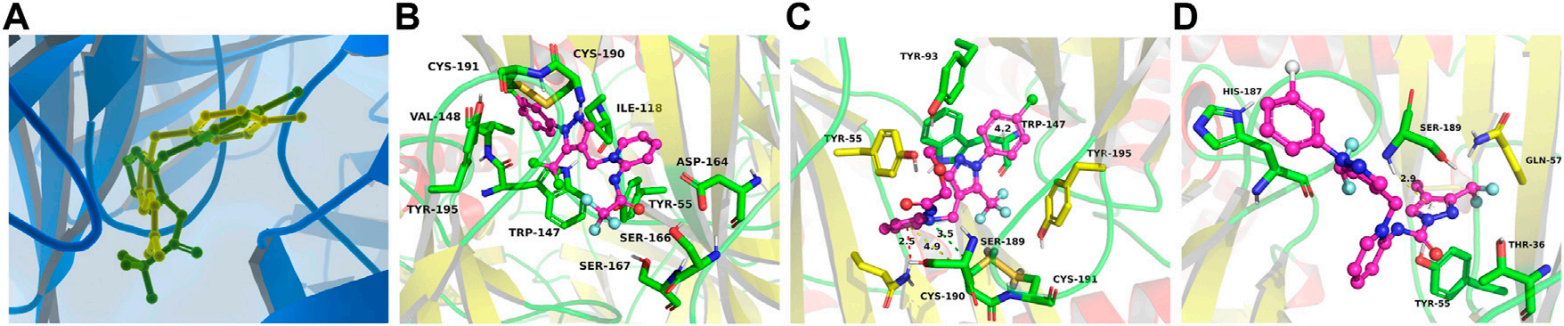
Simulated binding modes of compounds imidacloprid (yellow) and **FLP** (green) **(A)**, **A3 (B)**, **B4 (C)**, and **D6 (D)** with AChBP (PDB: 3c79).

In support of the structure–activity relationship, the representative compounds **A3**, **B4**, and **D6** were docked with the AChBP of *Aplysia californica*, which is a suitable structural surrogate for the insect nAChR ligand-binding domain (Onozaki et al., 2017). Two different popular docking programs, the Surflex-Dock and AutoDock Vina, were used for molecular docking to mutually verify the accuracy of simulation results (Table 4). Table 4 suggests that the docking results of the two programs are consistent. The control agent **FLP** showed the highest score (4.38) and the lowest predicted receptor affinity (-8.30 kcal/mol). The compound **B4** displayed a higher scoring value (3.69) and a lower affinity value (-6.00 kcal/mol) than the scoring value and predicted affinity of **A3** and **D6**, which is consistent with the insecticidal activities data of these compounds. It is worth noting that the predicted *K*_*i*_ value of the compound **B4** reached 39.99 uM, indicating that **B4** can bind to the receptor well. Figure 6 (**C**) showed that the compound **B4** was capable of forming hydrophobic, л-alkyl, and van der Waals interactions with hydrophobic amino acids (Tyr93, Trp147, and Tyr195). In addition, the N atom on the dearomatized pyridine ring of **B4** formed a hydrogen bond interaction with the sulfhydryl group of the residue Cys190, which may greatly promote the binding between **B4** and the receptor.

**TABLE 4:** Model evaluation and docking results by Sybyl x2.0 and AutoDock Vina procedures.

Sybyl				AutoDock Vina		
Compound	Total score	Crash	C score	Affinity (kcal/mol)	Estimated *K*_i_	Ligand efficiency
**FLP**	4.38	−2.84	2	−8.30	824.26 nM	−0.40
**A3**	3.13	−6.47	5	−4.70	0.36 mM	−0.16
**B4**	3.69	−6.21	3	−6.00	39.99 uM	−0.21
**D6**	3.15	−8.35	4	−4.20	0.83 mM	−0.12

Note: Ligand efficiency = $\frac{-∆G}{HA}$ , -∆G represents the binding free energy or docking score, and HA represents the number of heavy atoms of the ligand.

### Safety to bees

To further determine whether compound **B4** is safe for non-target biological insect pollinators, such as bees, acute oral toxicity and contact toxicity experiments of the **B4** and **FLP** to honeybees (*Apis mellifera*) were performed (Table 5). The toxicity test results revealed that compound **B4** and positive control **FLP** are low toxic to adult bees, with the LD_50_ of both oral toxicity and contact toxicity tests being >11.0 μg a.i./bee. This means when a broad-scale outbreak of *Plutella xylostella* occurs during the flowering period of nectar crops such as rape, the application of compound **B4** will not create a threat to the bees and damage the ecosystem.

**TABLE 5:** Bee toxicity (*Apis mellifera*) of **B4** and flupyrimin.

Compound	Bee toxicity	Time (h)	Mortality	Toxic regression equation	95% CI	LD_50_	Toxicity level
B4	Contact toxicity	24	3	—	—	>11.0	Low toxicity
		48	3	—	—	>11.0	Low toxicity
	Oral toxicity	24	15	—	—	>11.0	Low toxicity
		48	17	—	—	>11.0	Low toxicity
Flupyrimin	Contact toxicity	24	12	—	—	>11.0	Low toxicity
		48	20	y = −1.841x+0.027	59.48–76.55	67.61	Low toxicity
	Contact toxicity	24	9	—	—	>11.0	Low toxicity
		48	13	—	—	>11.0	Low toxicity

## Conclusion

In conclusion, using flupyrimin as a lead compound, a series of novel compounds were designed and synthesized based on a molecular hybridization strategy to combine the active moiety arylpyrazolyl with the pyridinimide ring. The bioassay results showed that the title compounds displayed good insecticidal activities against *Plutella xylostella*, and the lethality of nine compounds (**A3**, **B1–B6**, **D4**, and **D6**) reached 100% at 400 μg/ml. In the meantime, when the concentration dropped to 25 μg/ml, the insecticidal activities against *Plutella xylostella* for compounds **B2**, **B3**, and **B4** reached more than 70%. The analysis of the structure–activity relationship for the title compounds against *Plutella xylostella* showed that when R_3_ is methoxymethyl moiety, the activities of the title compounds are generally better, as well as when R_1_ is a trifluoromethyl group, the activities are higher than those of the difluoromethyl group, which provides guidance for our further development of insecticides in the future. Also, from the results of DFT calculations of compound **B4** and **FLP**, the energy gaps between the HOMO and LUMO, ClogP, and TPSA of compound **B4** and **FLP** are different. Furthermore, the DFT calculation results and docking results provided meaningful information to design higher-activity insecticides. The toxicity experiment tests also evidenced that **B4** is low toxic to adult bees. Combining with the abovementioned experimental results, further structural optimization work is underway and will be reported in the future.

## Data Availability

The original contributions presented in the study are included in the article/Supplementary Material; further inquiries can be directed to the corresponding authors.
