# Supplementary material for: Design, synthesis, and insecticidal activity of a novel series of flupyrimin analogs bearing 1-aryl-1H-pyrazol-4-yl subunits
Source: Front Chem. 2022 Oct 3;10:1019573. doi: 10.3389/fchem.2022.1019573 (PMC9574050; doi:10.3389/fchem.2022.1019573)
Supplement: Supplementary file 1 [file DataSheet1.PDF]

Supporting Material

Design, synthesis and insecticidal activity of a novel series of flupyrimin analogues bearing 1-aryl-1H-pyrazol-4-yl subunits

Fenghai Zhao†, Xianjun Tang†, Jiaxing Huang*, Jiaqi Li, Yumei Xiao, Zhaohai Qin*

College of science, China Agricultural University, Beijing 100193, China

* Corresponding author. J. X. Huang, Tel.(Fax):86-10-62732873; E-mail: huangjiaxing@cau.edu.cn; Z. H. Qin, Tel.(Fax):86-10-62732958; E-mail: qinzhaohai@263.net

A1

H-NMR

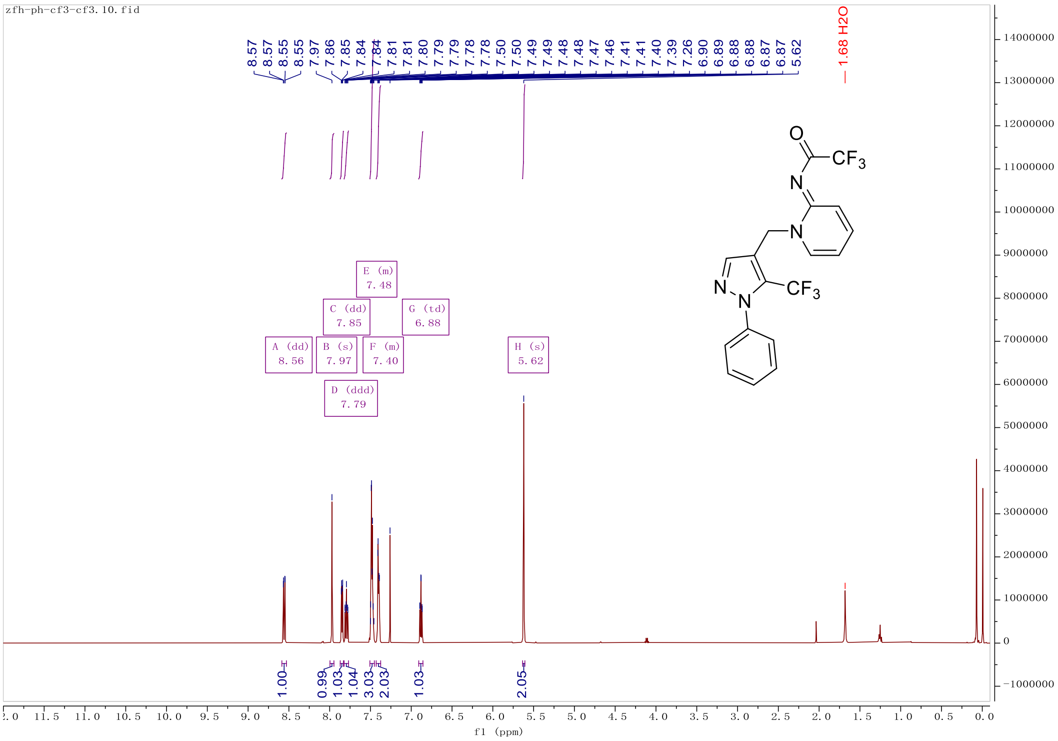

^{13}C-NMR

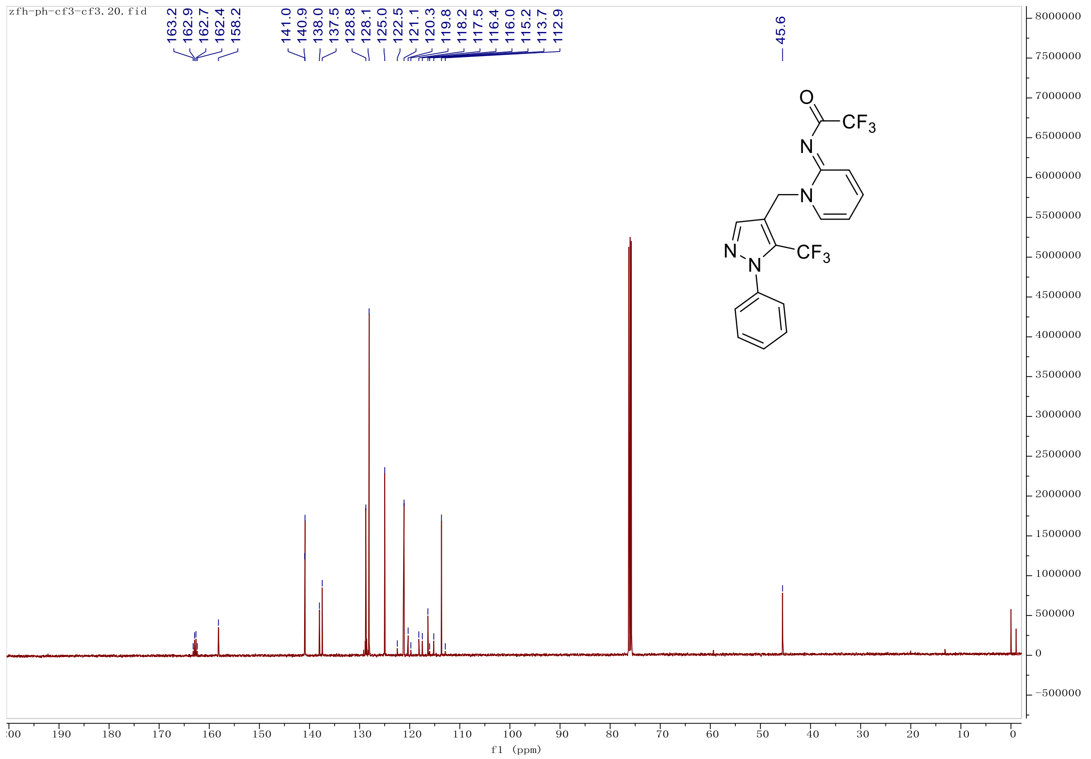

HIGH RESLUTION MASS SPECTROMETRY REPORT

Sample No.	Formula (M)	Ion Formula	Measured m/z	Calc m/z	Diff (ppm)
**A1**	$C_{18}H_{12}F_6N_4O$	$[M+H]^+$	415.0988	415.0988	0

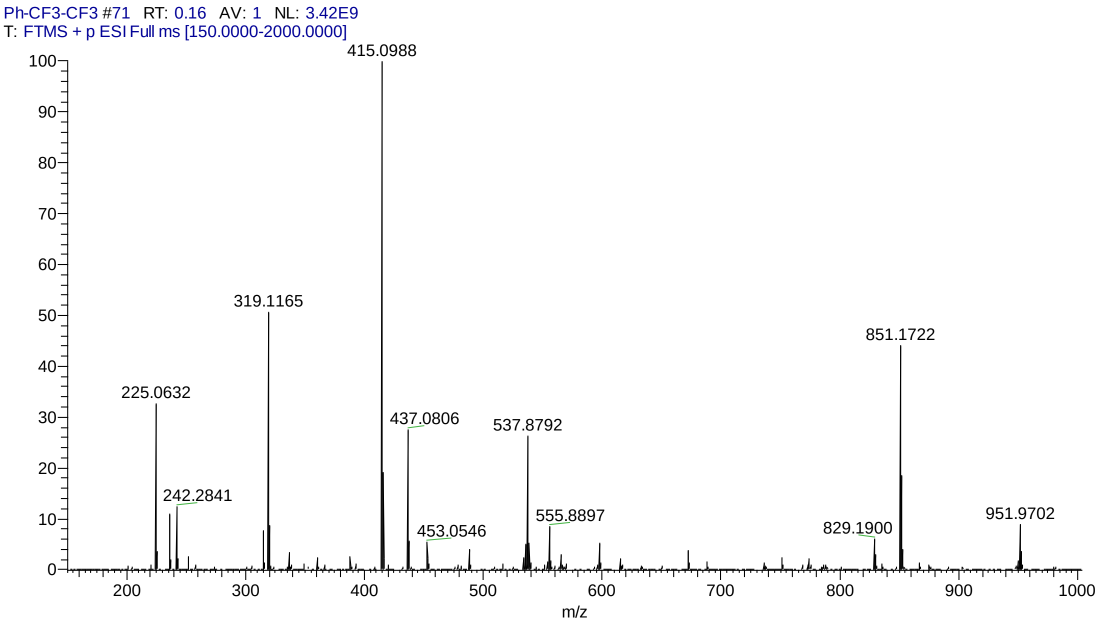

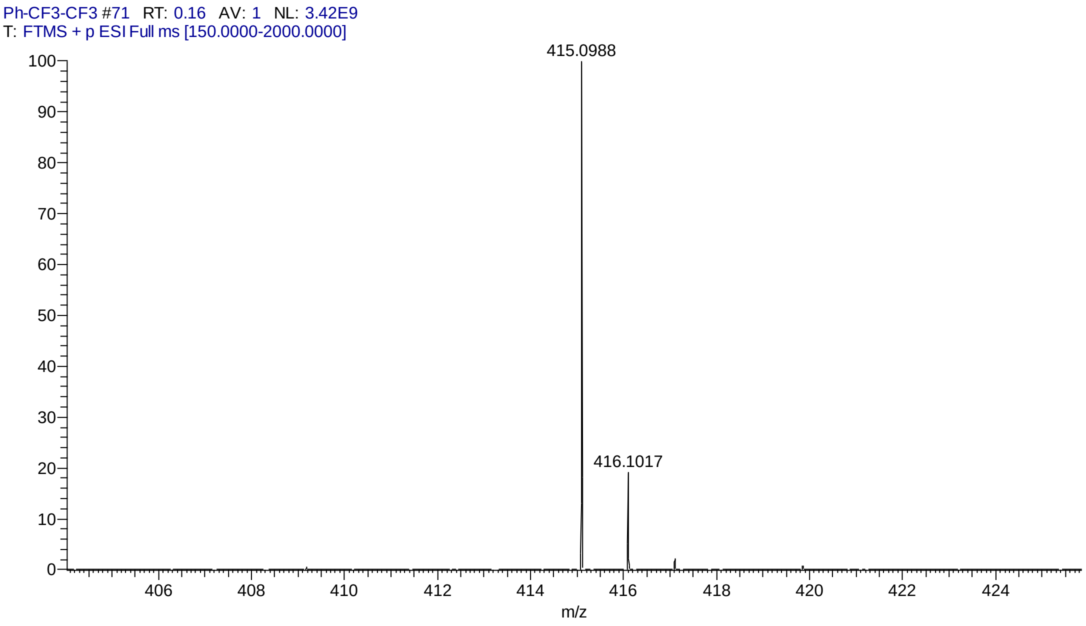

A2

H-NMR

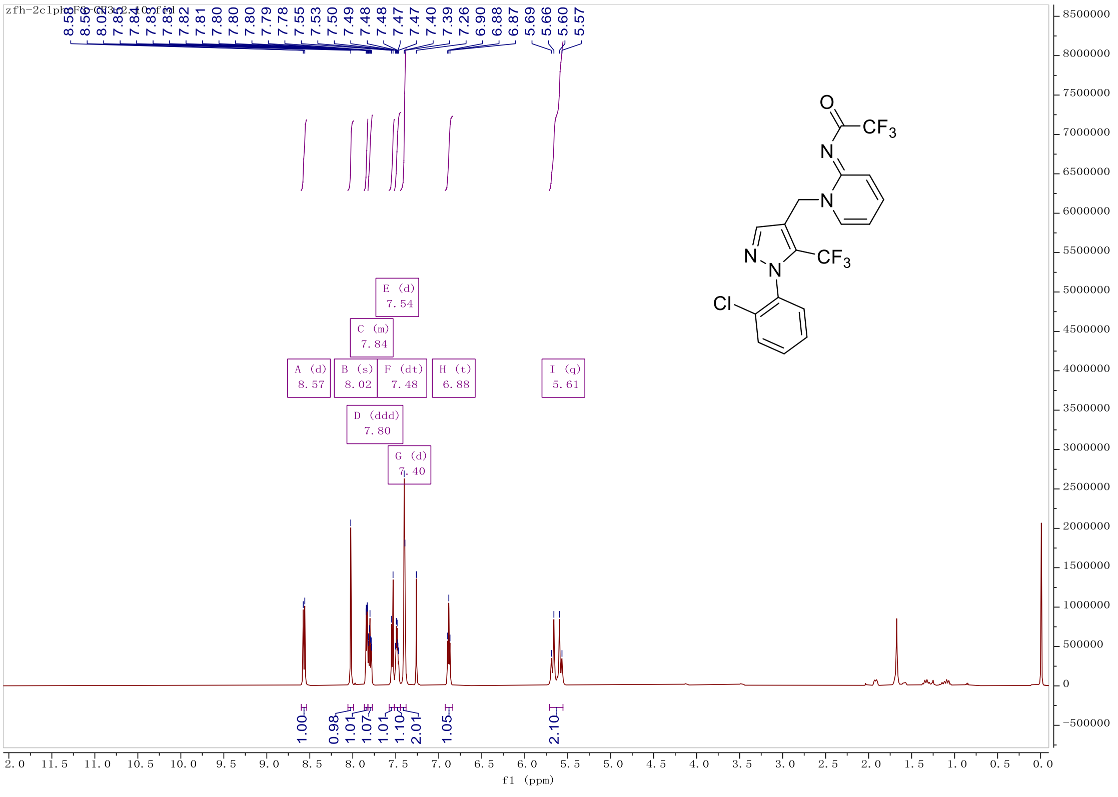

^{13}C-NMR

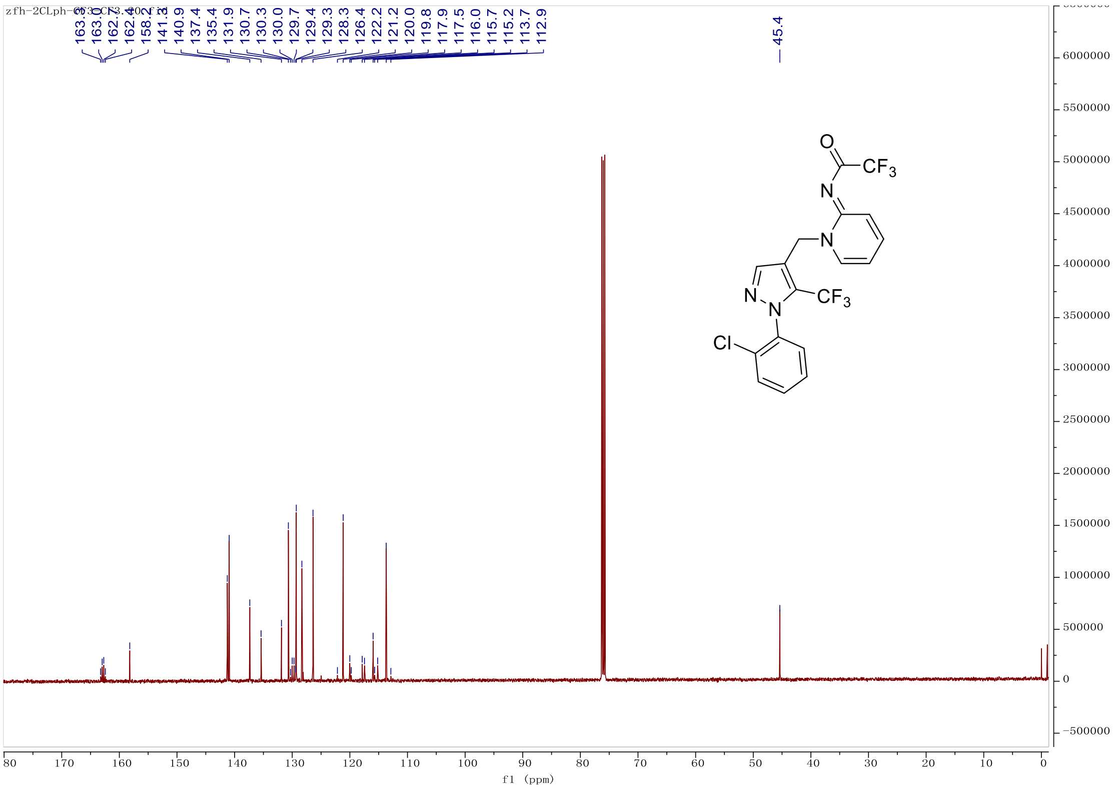

HIGH RESLUTION MASS SPECTROMETRY REPORT

Sample No.	Formula (M)	Ion Formula	Measured m/z	Calc m/z	Diff (ppm)
**A2**	$C_{18}H_{11}ClF_6N_4O$	[M+H]+	449.0597	449.0598	-0.22

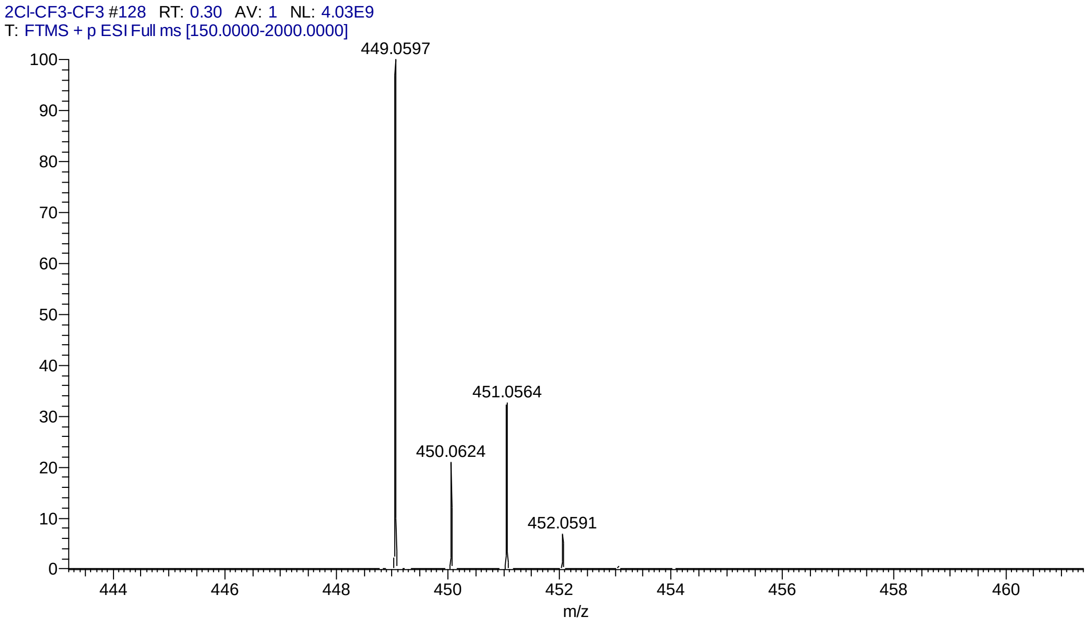

A3

H-NMR

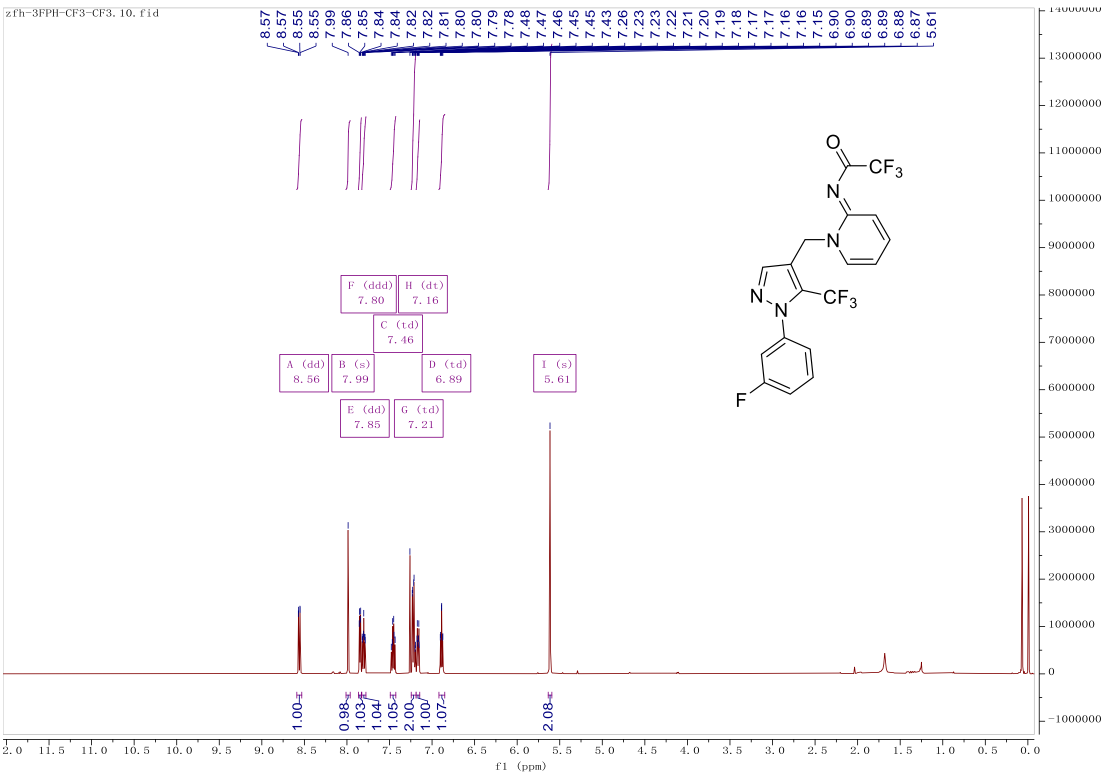

^{13}C-NMR

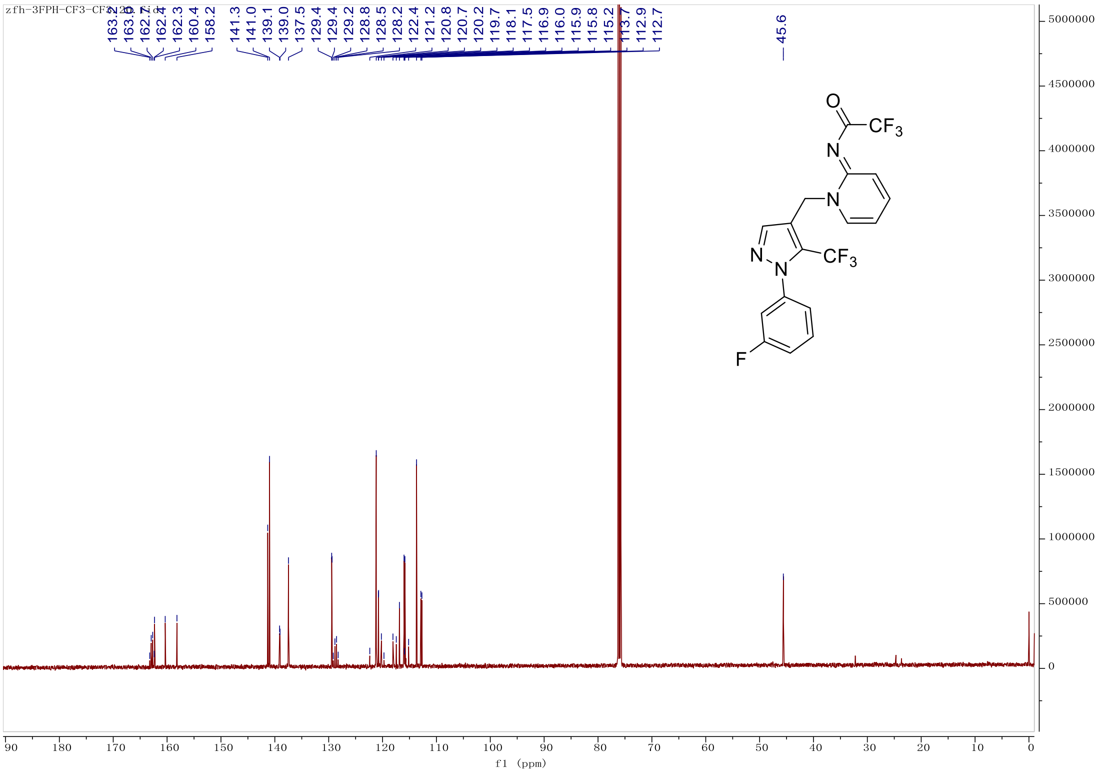

HIGH RESLUTION MASS SPECTROMETRY REPORT

Sample No.	Formula (M)	Ion Formula	Measured m/z	Calc m/z	Diff (ppm)
**A3**	C_{18}H11F_7N_4O	$[M+H]^+$	433.0892	433.0894	-0.46

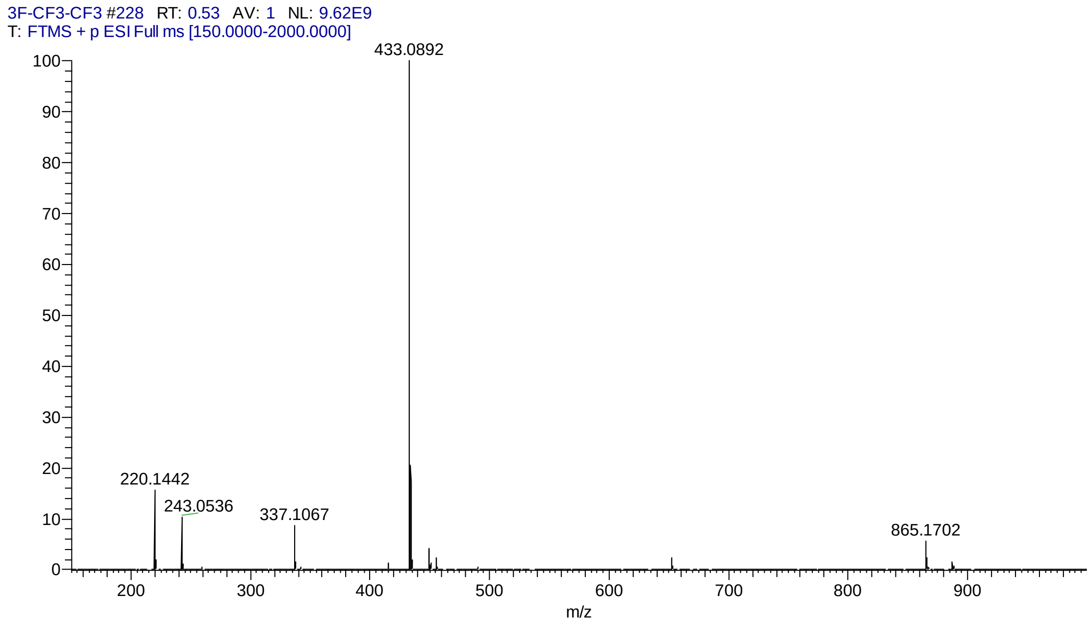

A4

H-NMR

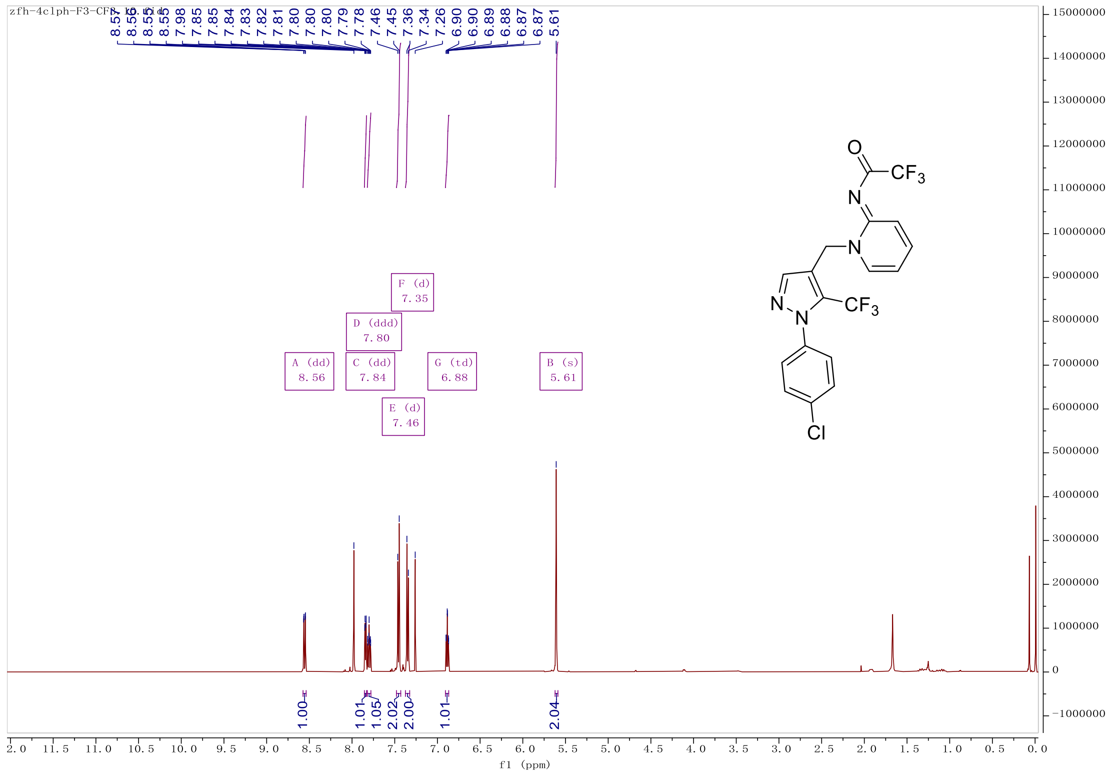

^{13}C-NMR

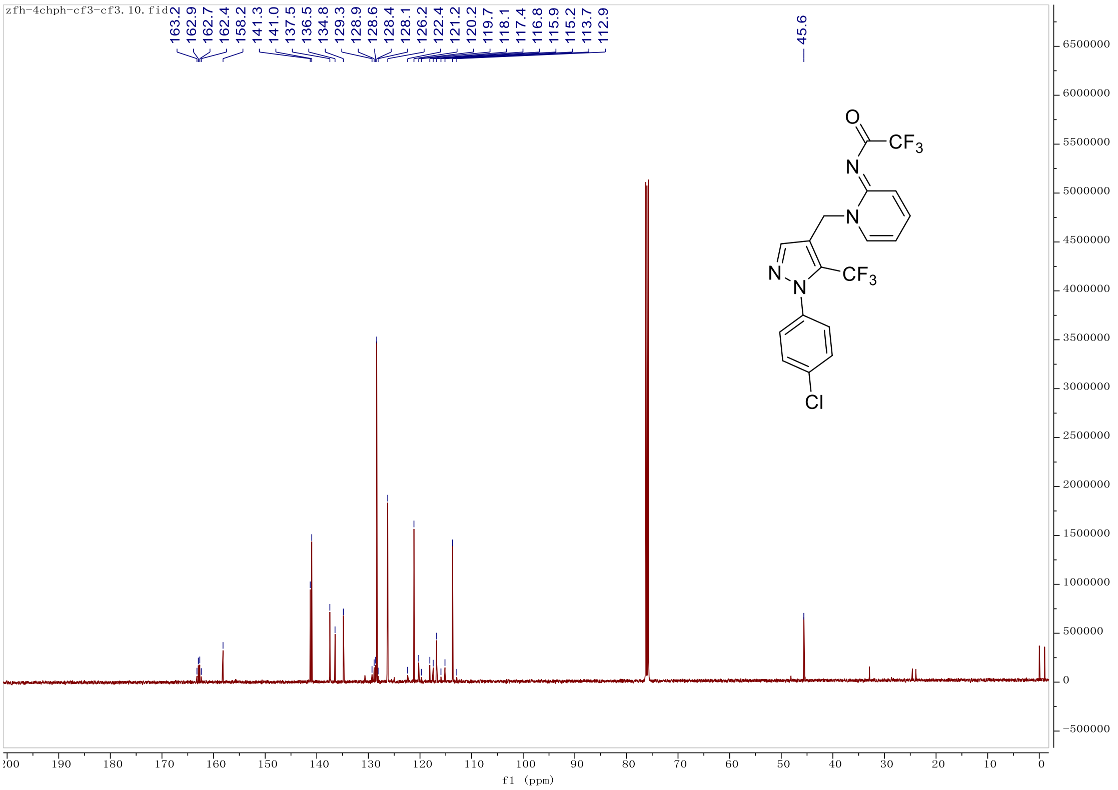

HIGH RESLUTION MASS SPECTROMETRY REPORT

Sample No.	Formula (M)	Ion Formula	Measured m/z	Calc m/z	Diff (ppm)
**A4**	$C_{18}H_{11}ClF_6N_4O$	$[M+H]^+$	449.0596	449.0598	0

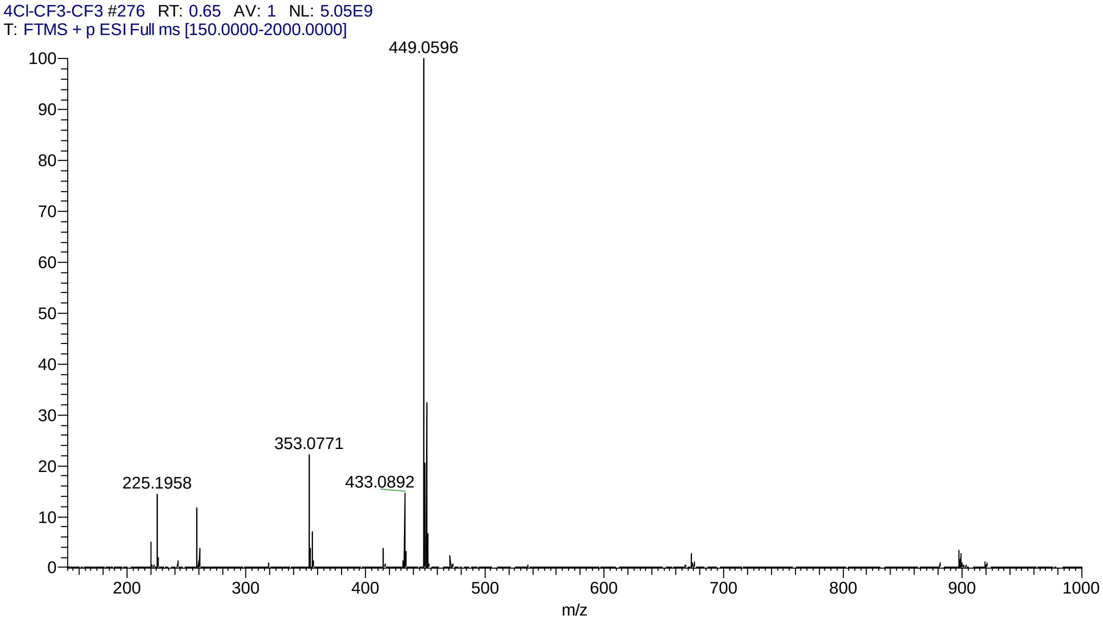

A5

H-NMR

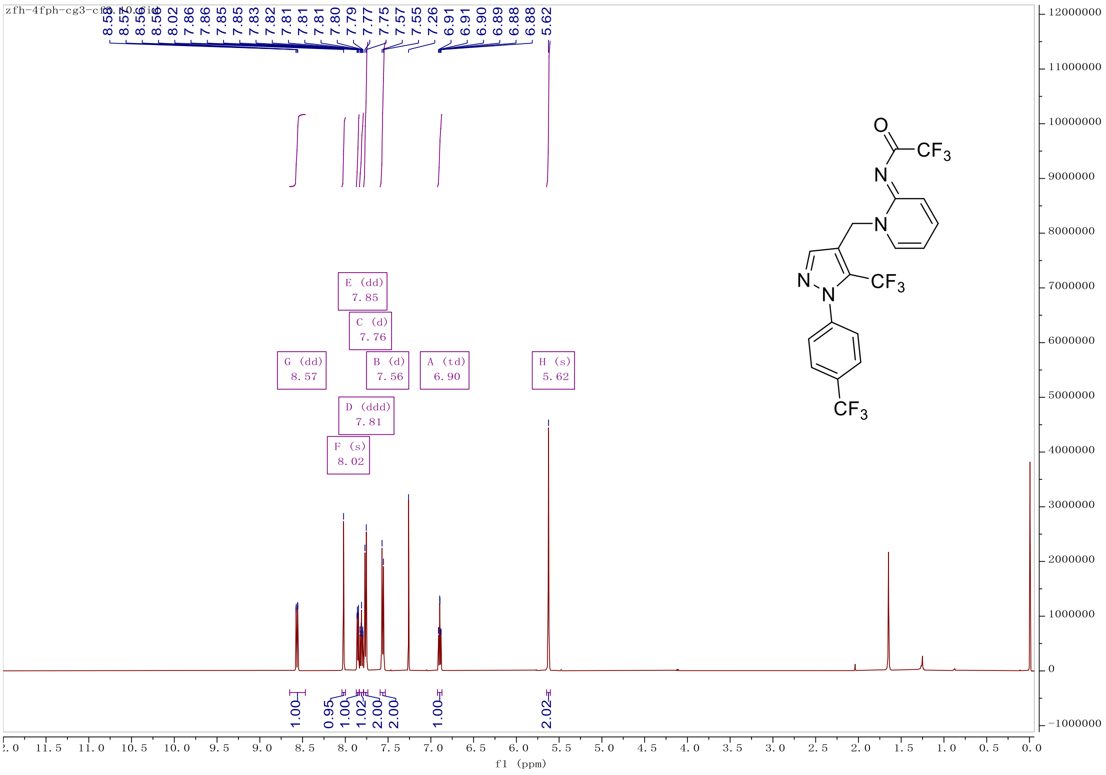

^{13}C-NMR

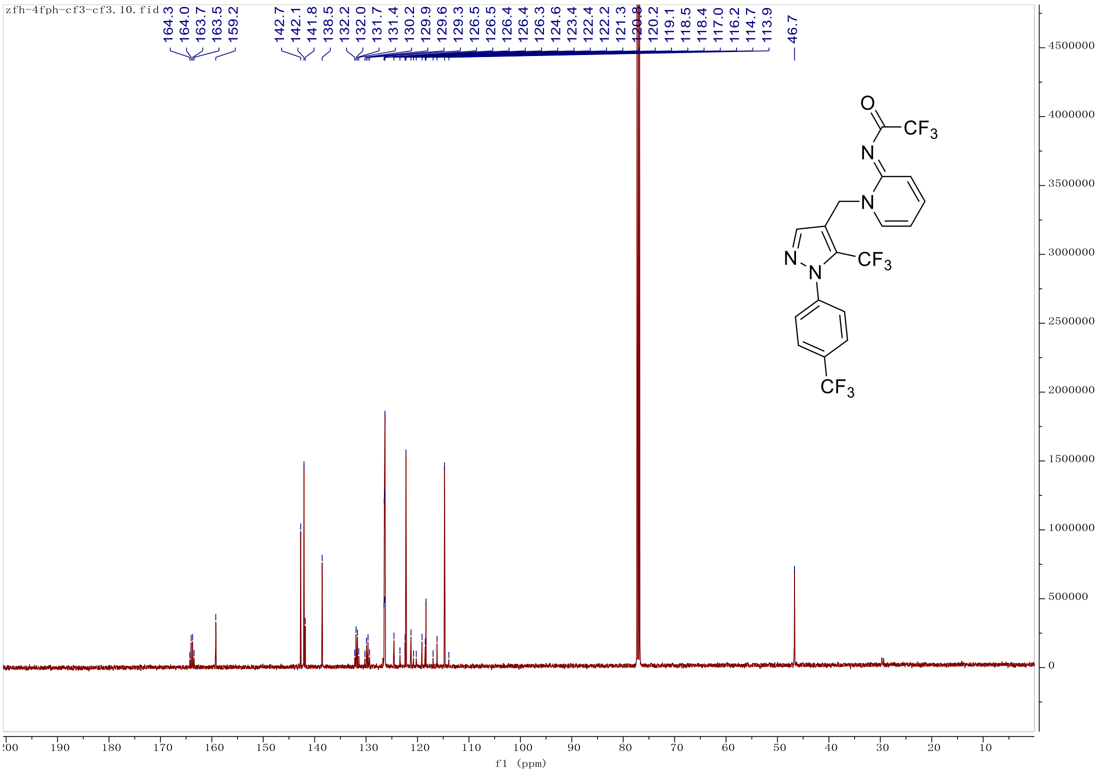

HIGH RESLUTION MASS SPECTROMETRY REPORT

Sample No.	Formula (M)	Ion Formula	Measured m/z	Calc m/z	Diff (ppm)
**A5**	$C_{18}H_{11}F_7N_4O$	$[M+H]^+$	433.0893	433.0894	-0.23

	$C_{19}H_{11}F_9N_4O$	$[M+H]^+$	483.0860	483.0862	-0.41

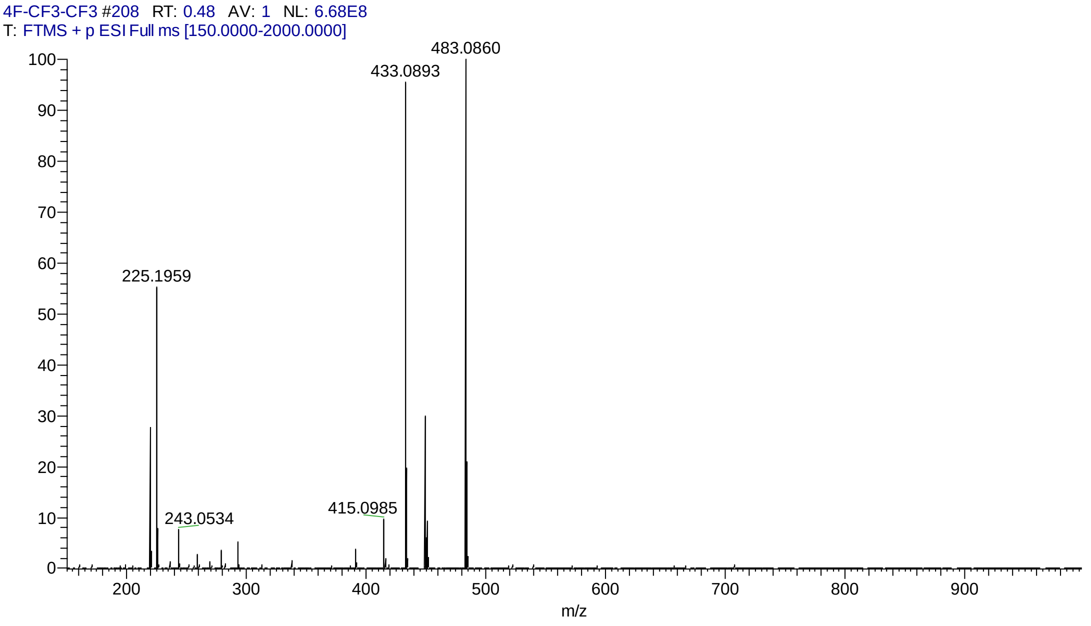

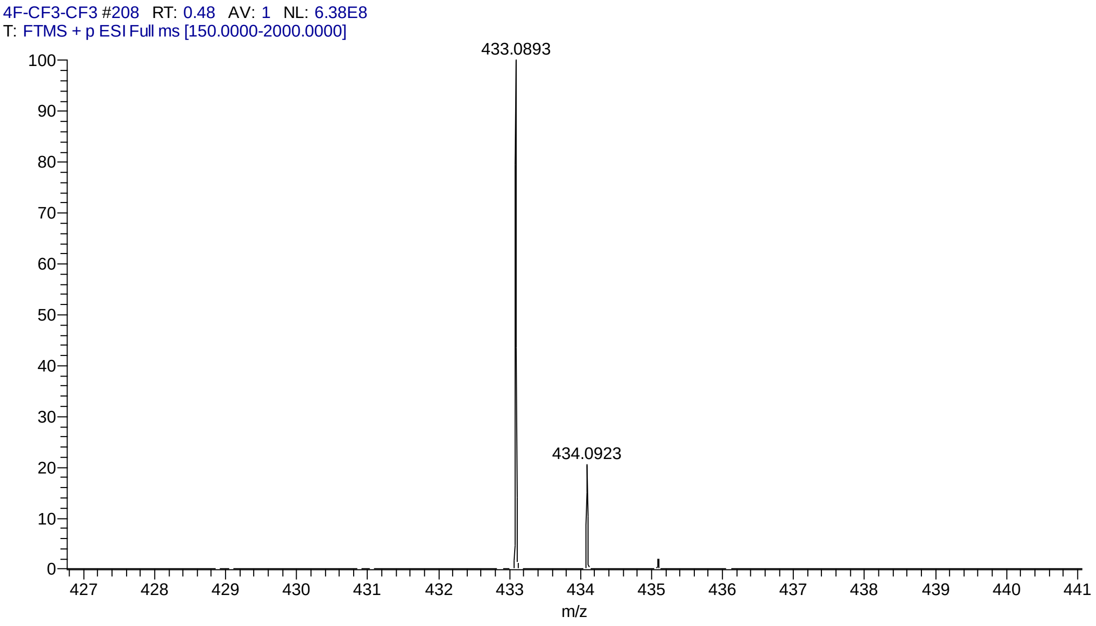

A6

H-NMR

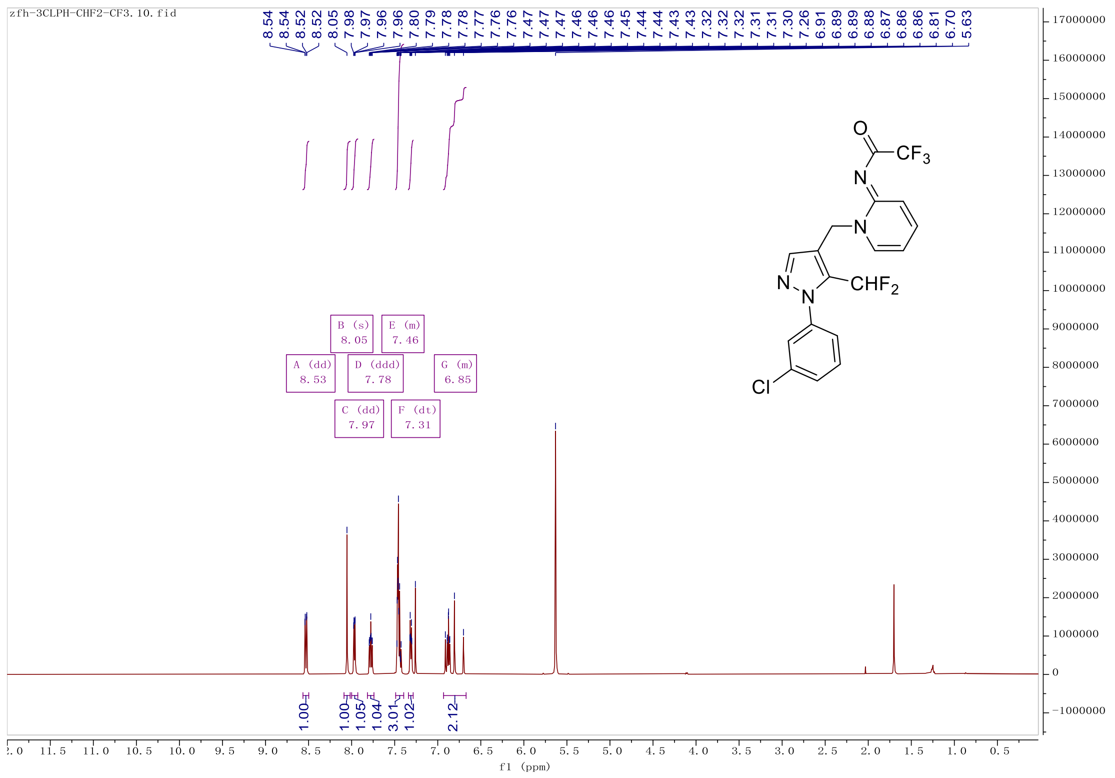

^{13}C-NMR

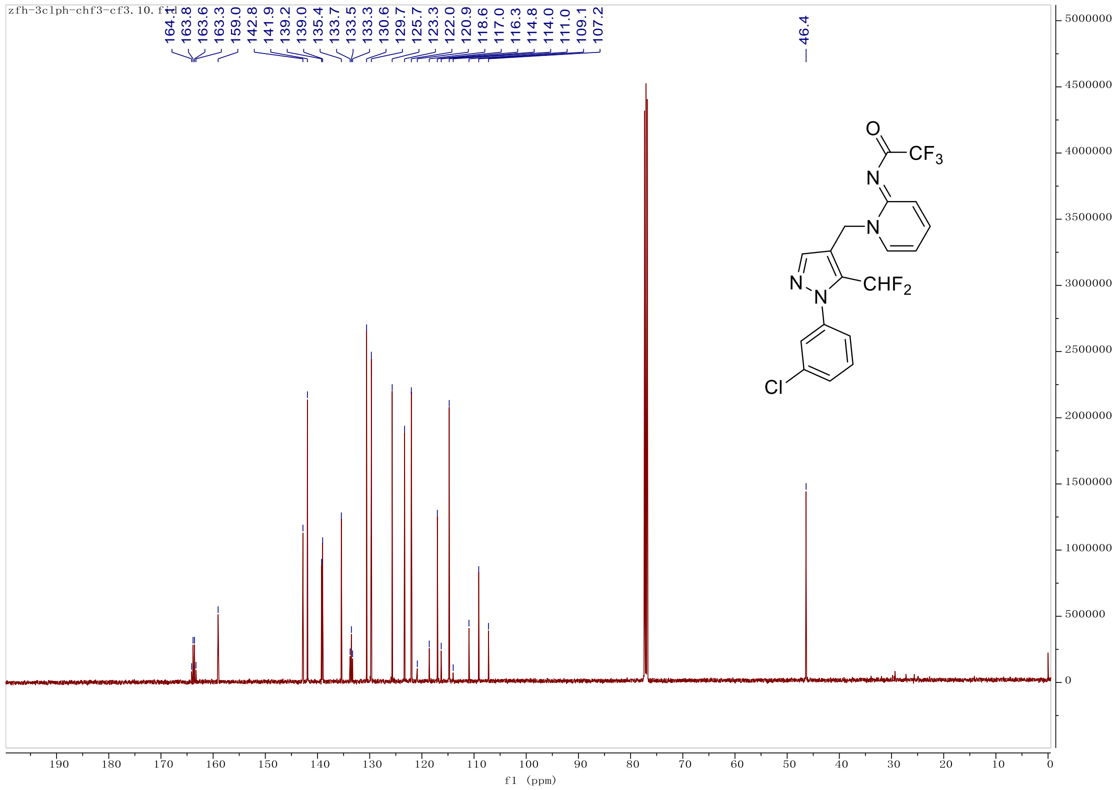

HIGH RESLUTION MASS SPECTROMETRY REPORT

Sample No.	Formula (M)	Ion Formula	Measured m/z	Calc m/z	Diff (ppm)
**A6**	$C_{18}H_{12}ClF_5N_4O$	$[M+H]^+$	431.0692	431.0693	-0.23

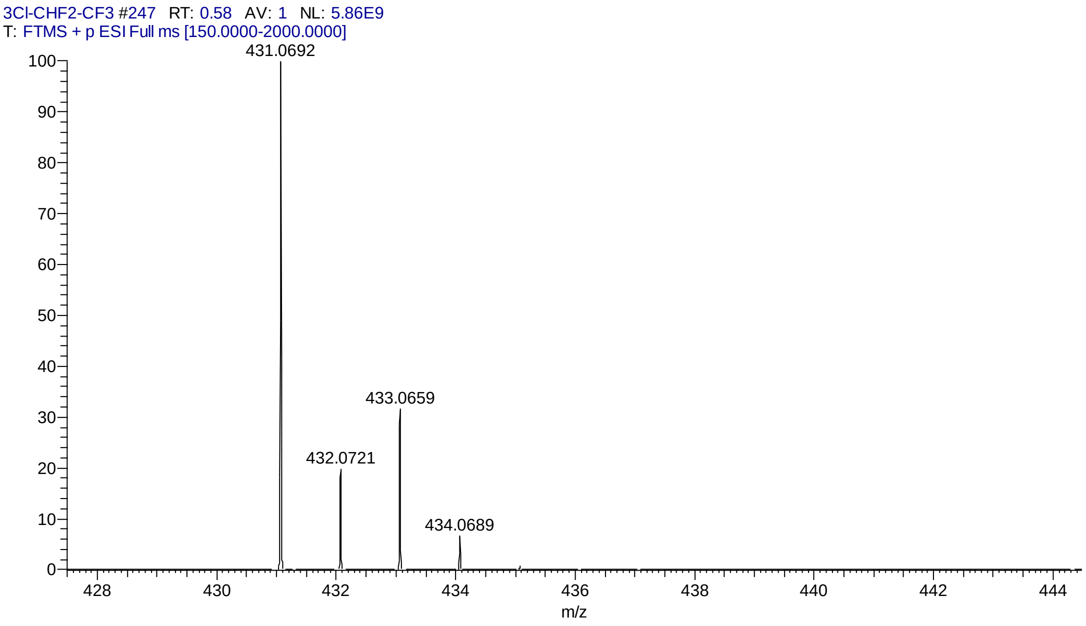

A7

H-NMR

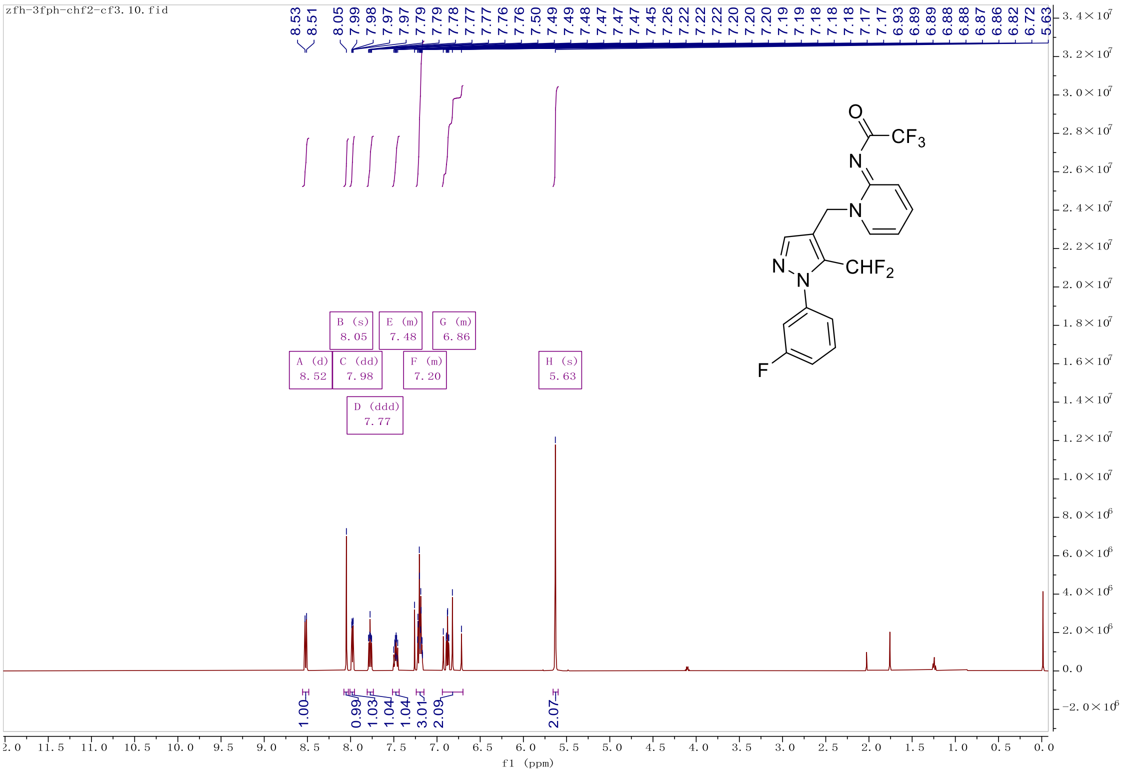

^{13}C-NMR

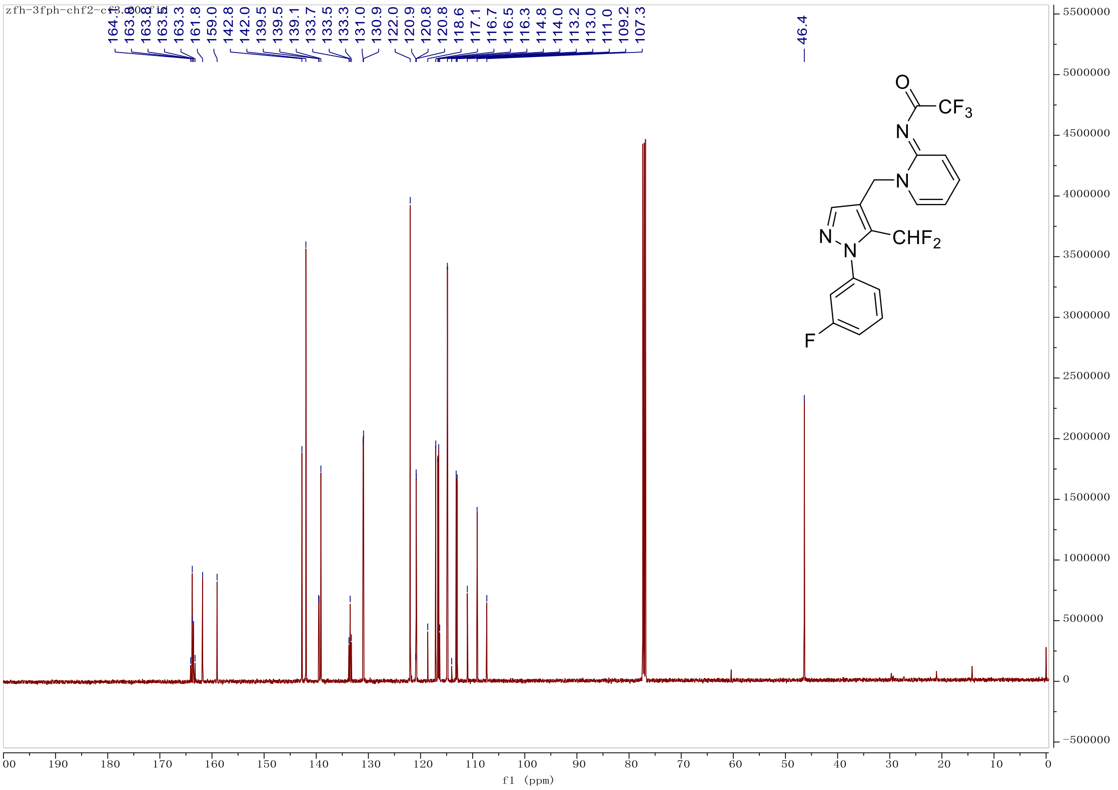

HIGH RESLUTION MASS SPECTROMETRY REPORT

Sample No.	Formula (M)	Ion Formula	Measured m/z	Calc m/z	Diff (ppm)
**A7**	$C_{18}H_{12}F_6N_4O$	$[M+H]^+$	415.0985	415.0988	-0.72

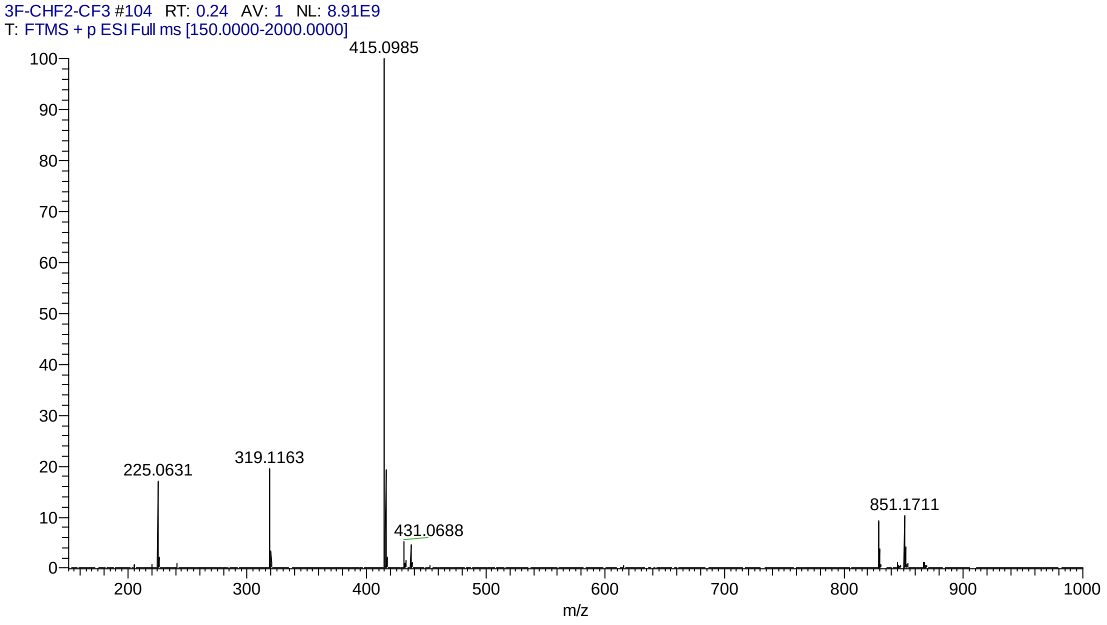

A8

H-NMR

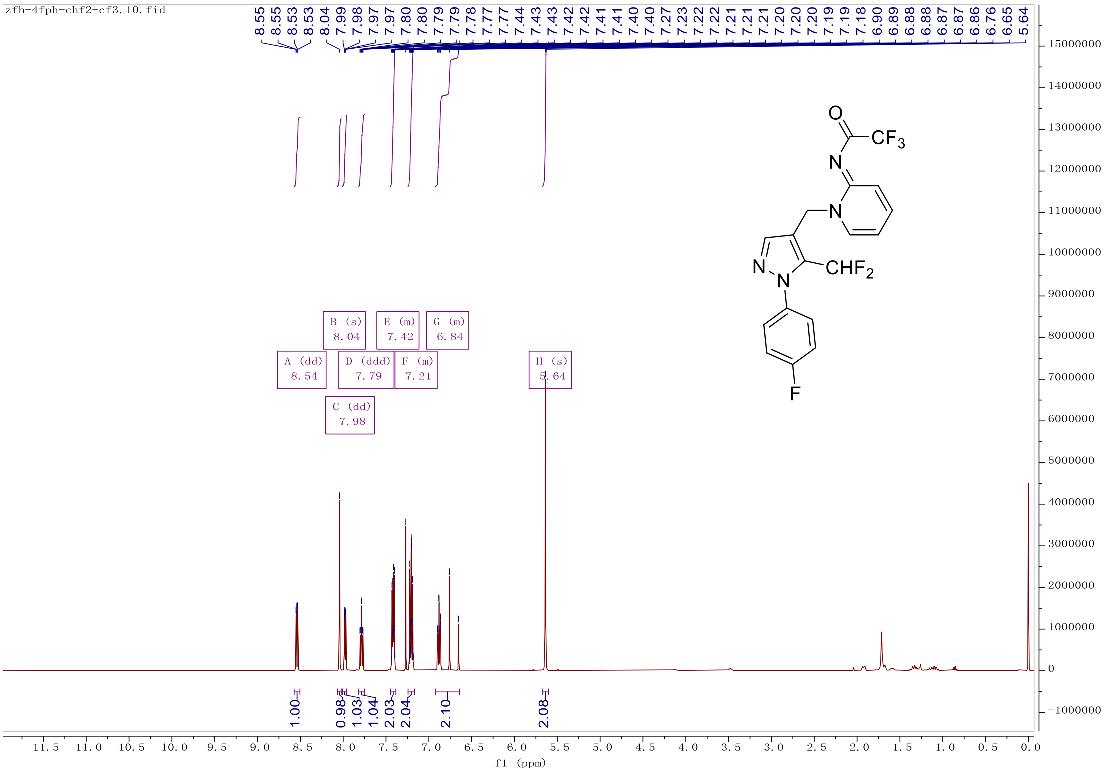

^{13}C-NMR

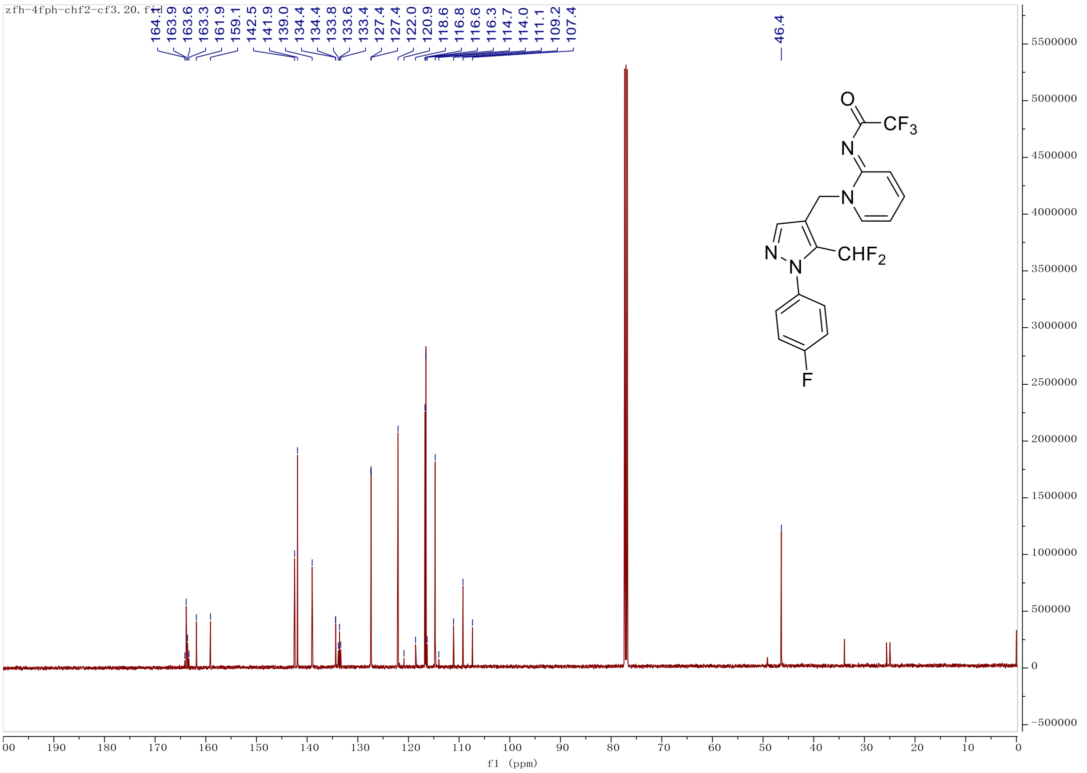

HIGH RESLUTION MASS SPECTROMETRY REPORT

Sample No.	Formula (M)	Ion Formula	Measured m/z	Calc m/z	Diff (ppm)
**A8**	$C_{18}H_{12}F_6N_4O$	$[M+H]^+$	415.0986	415.0988	-0.48

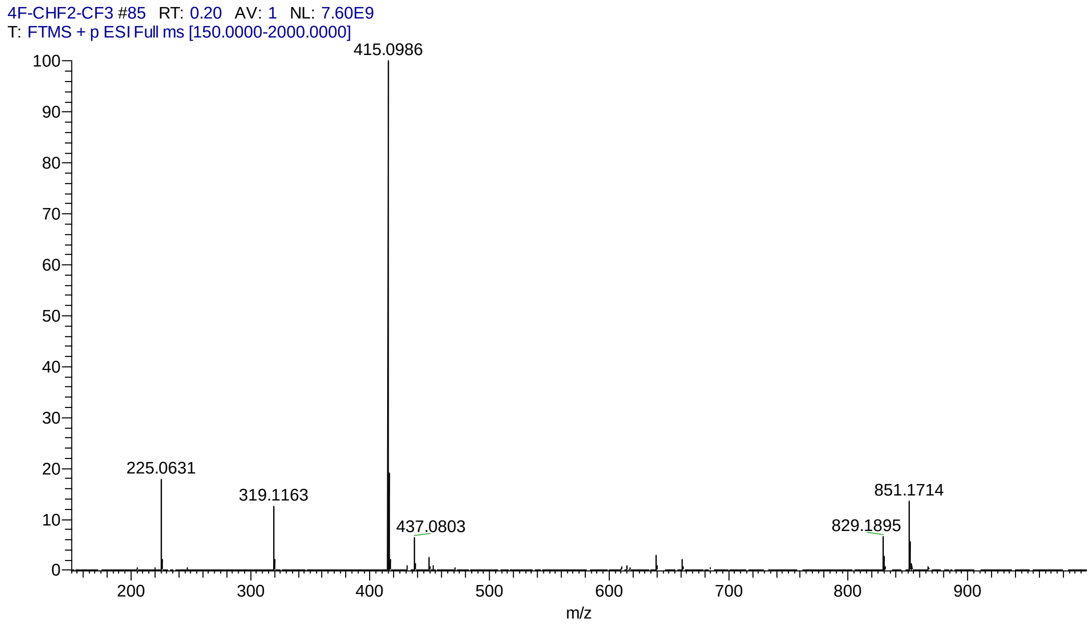

B1

H-NMR

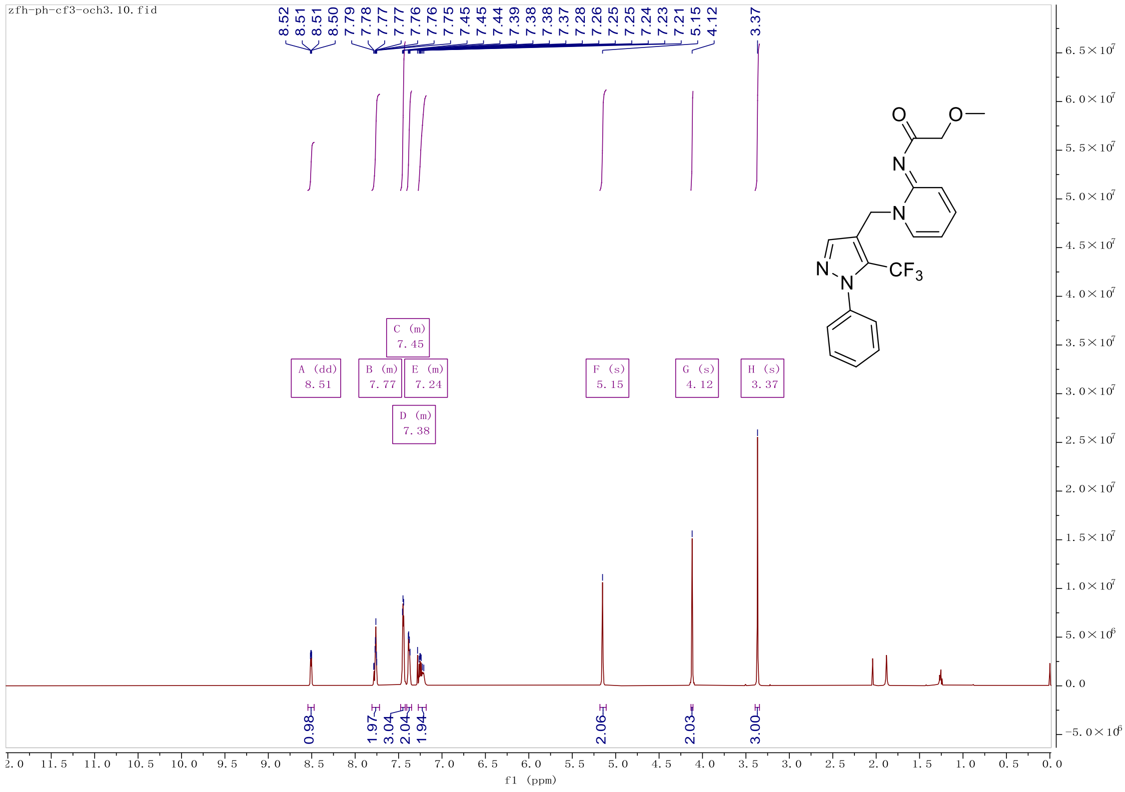

^{13}C-NMR

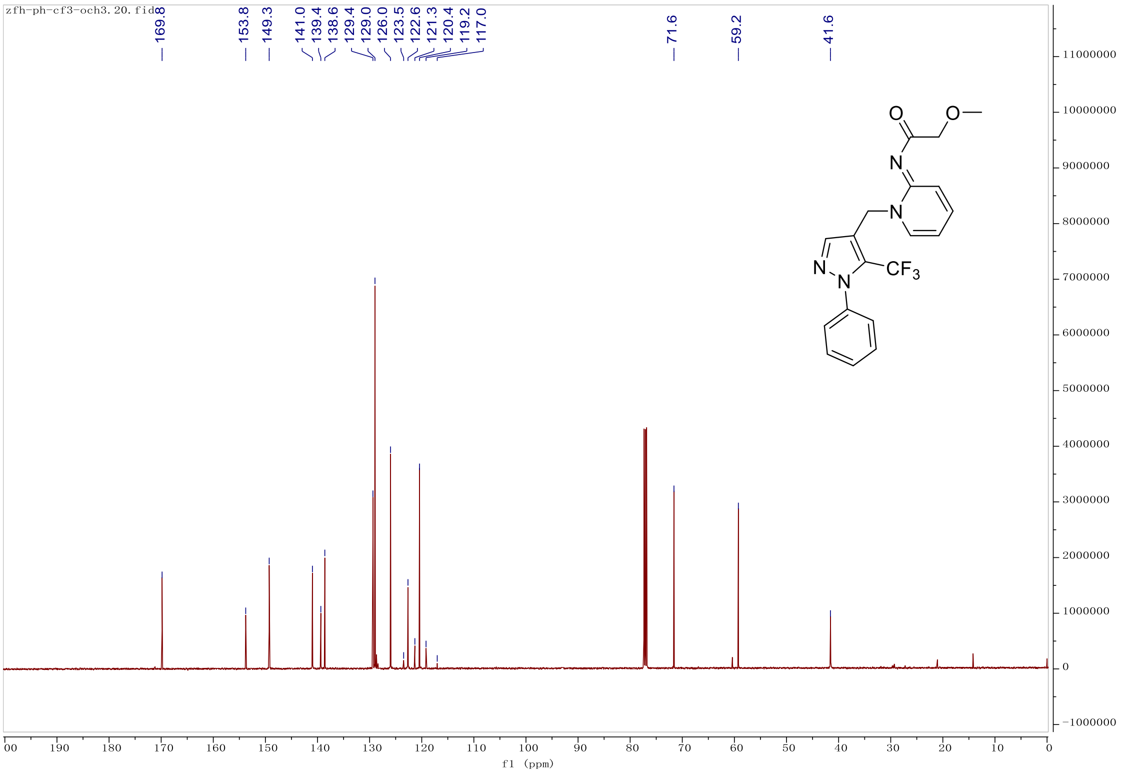

HIGH RESLUTION MASS SPECTROMETRY REPORT

Sample No.	Formula (M)	Ion Formula	Measured m/z	Calc m/z	Diff (ppm)
**B1**	$C_{19}H_{17}F_3N_4O_2$	$[M+H]^+$	391.1374	391.1376	-0.51

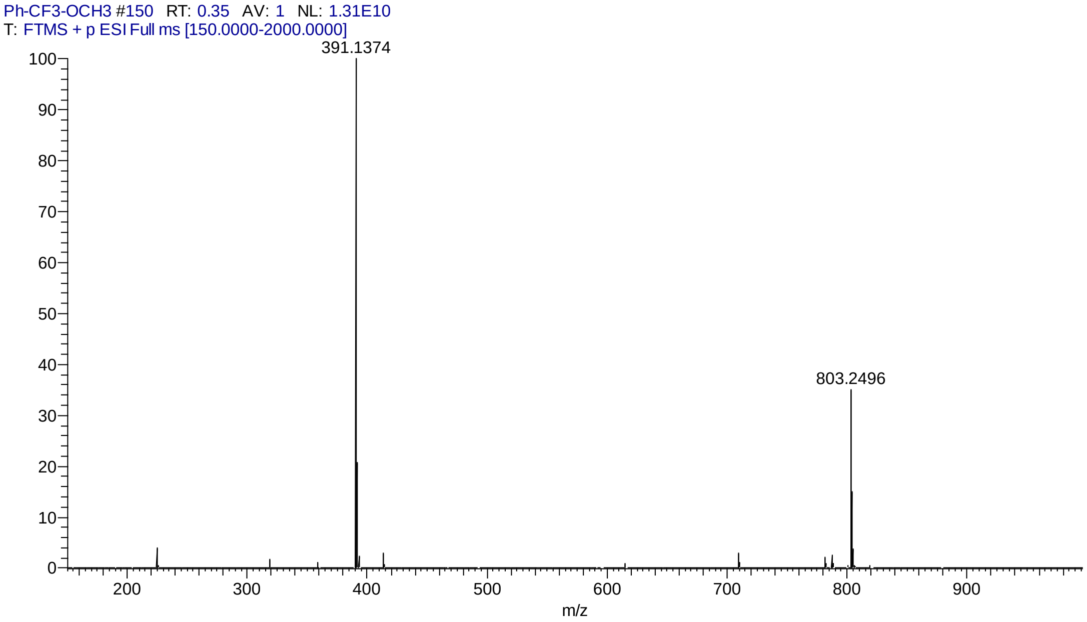

B2

H-NMR

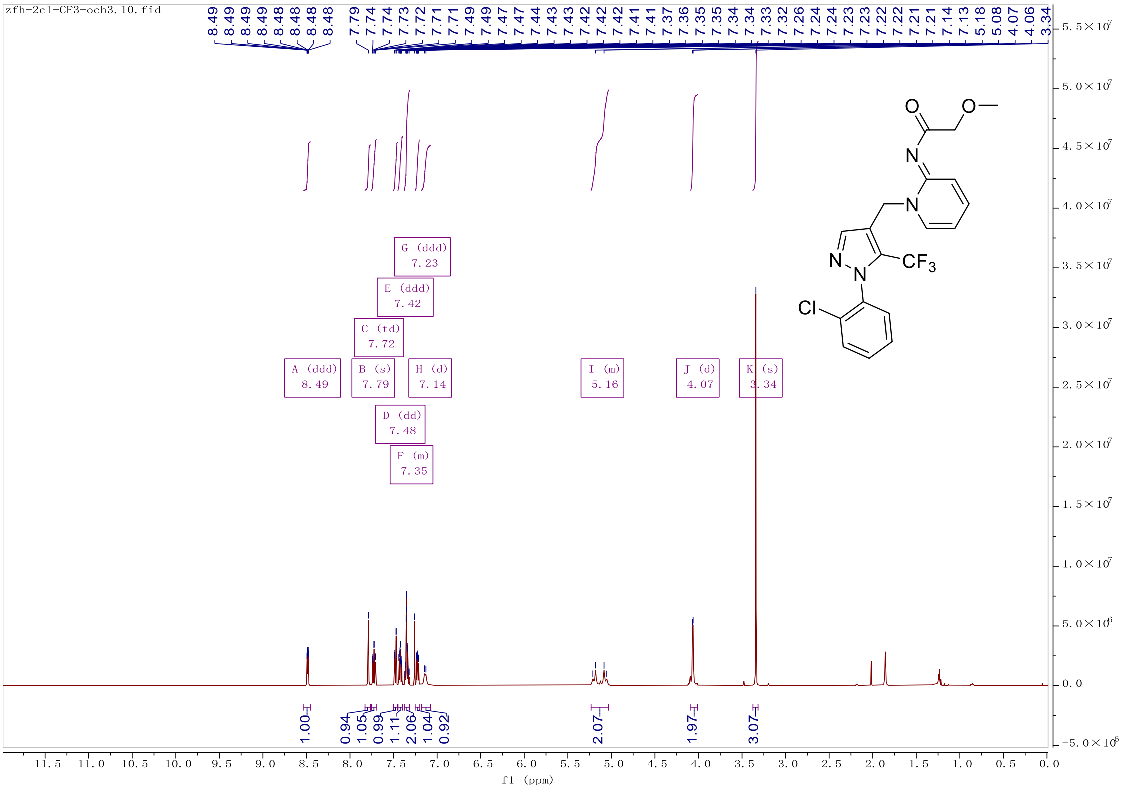

^{13}C-NMR

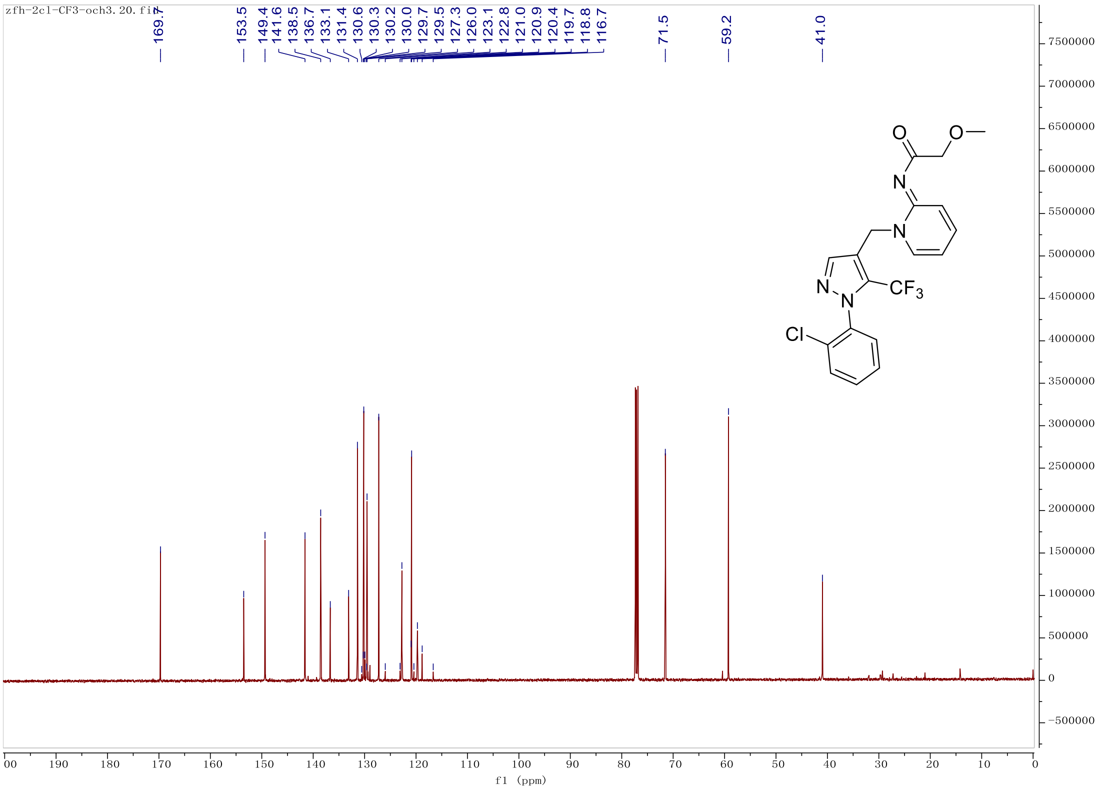

HIGH RESLUTION MASS SPECTROMETRY REPORT

Sample No.	Formula (M)	Ion Formula	Measured m/z	Calc m/z	Diff (ppm)
**B2**	$C_{19}H_{16}ClF_3N_4O_2$	$[M+H]^+$	425.0984	425.0987	-0.71

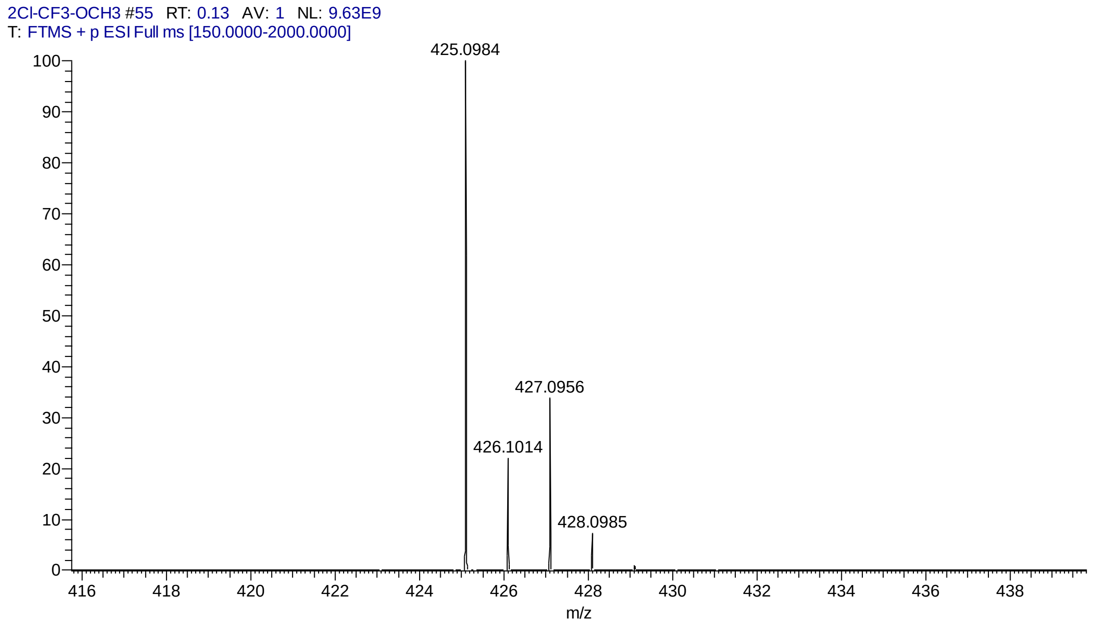

B3

H-NMR

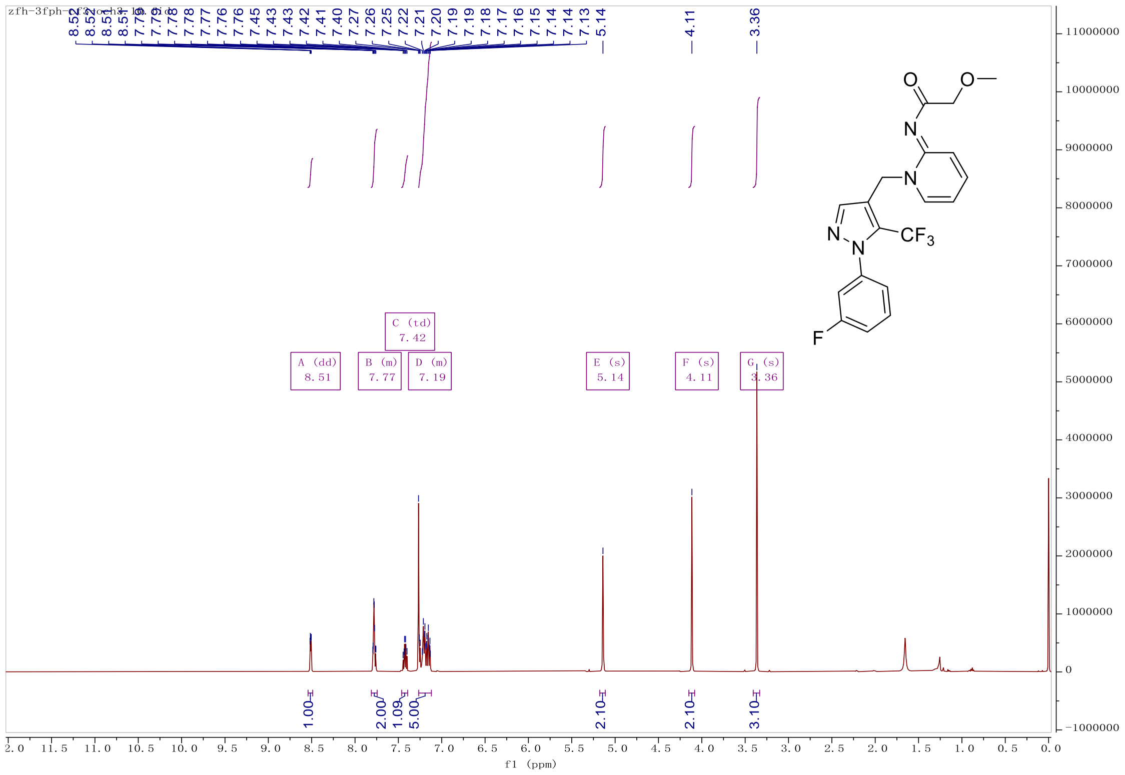

^{13}C-NMR

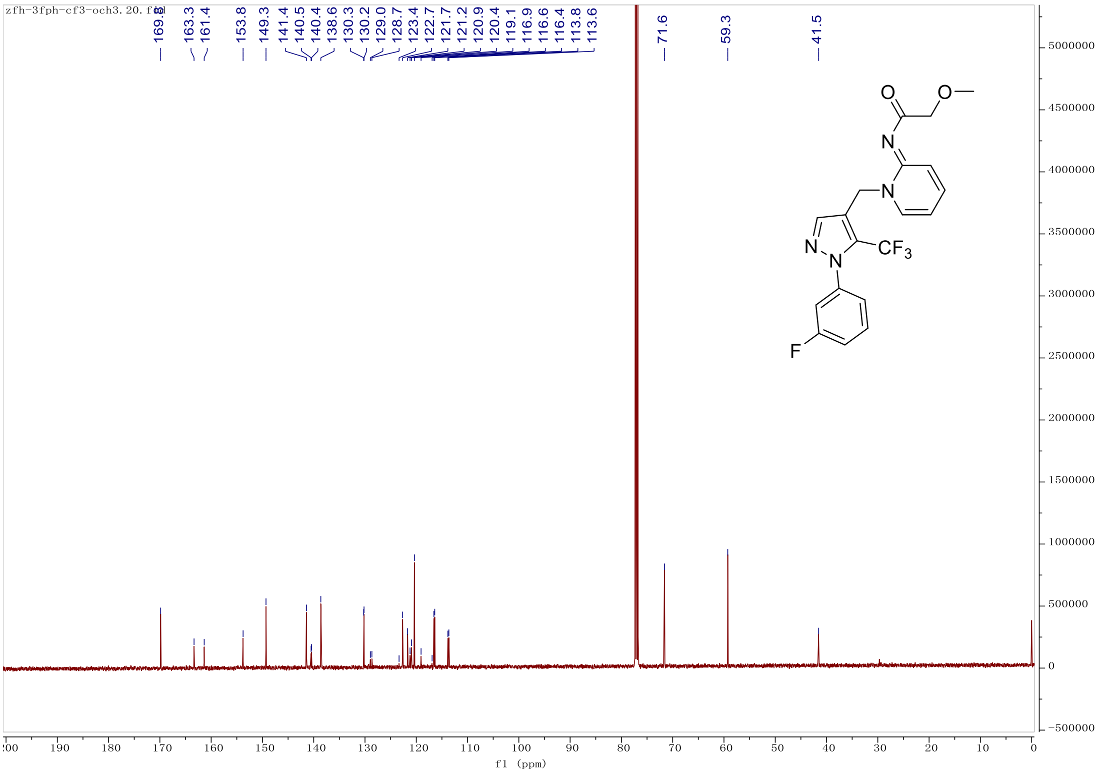

HIGH RESLUTION MASS SPECTROMETRY REPORT

Sample No.	Formula (M)	Ion Formula	Measured m/z	Calc m/z	Diff (ppm)
**B3**	$C_{19}H_{16}F_4N_4O_2$	$[M+H]^+$	409.1280	409.1282	-0.49

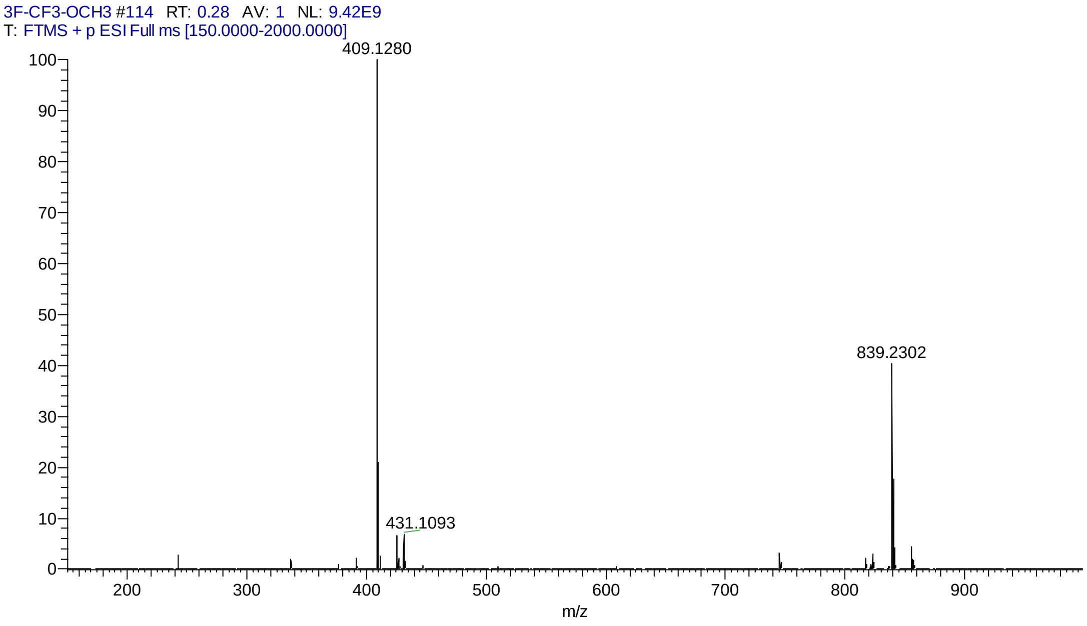

B4

H-NMR

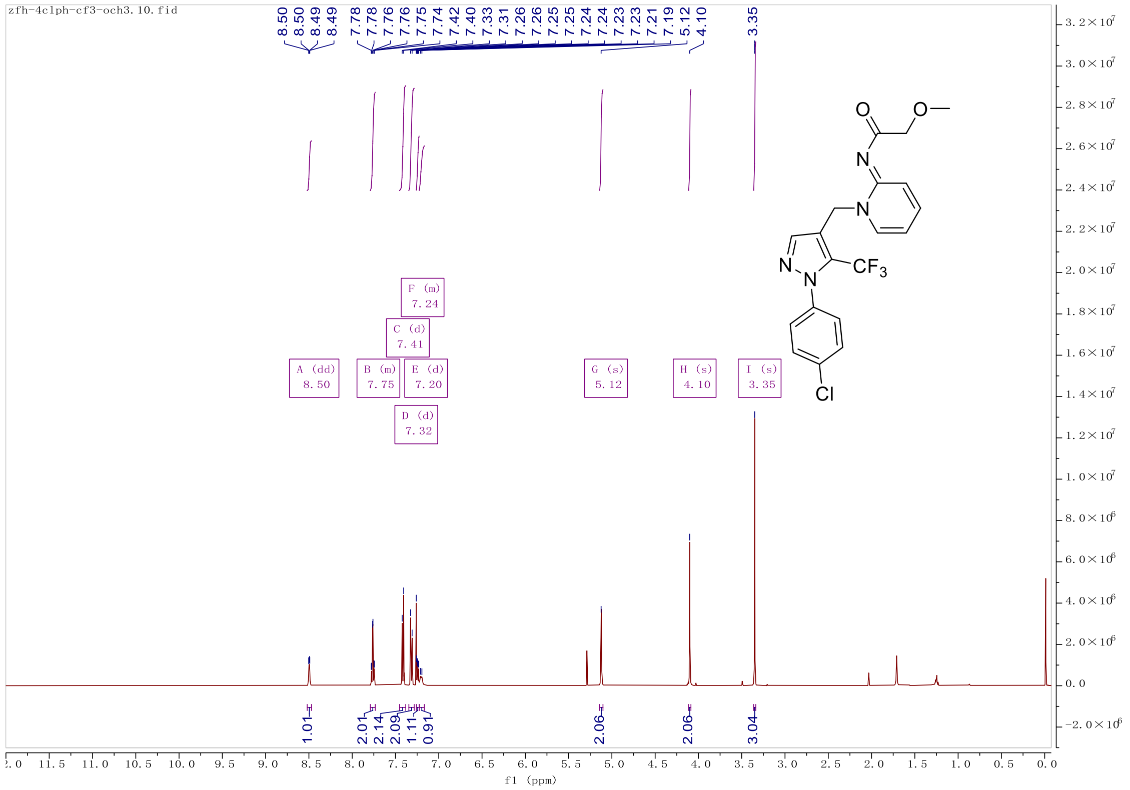

^{13}C-NMR

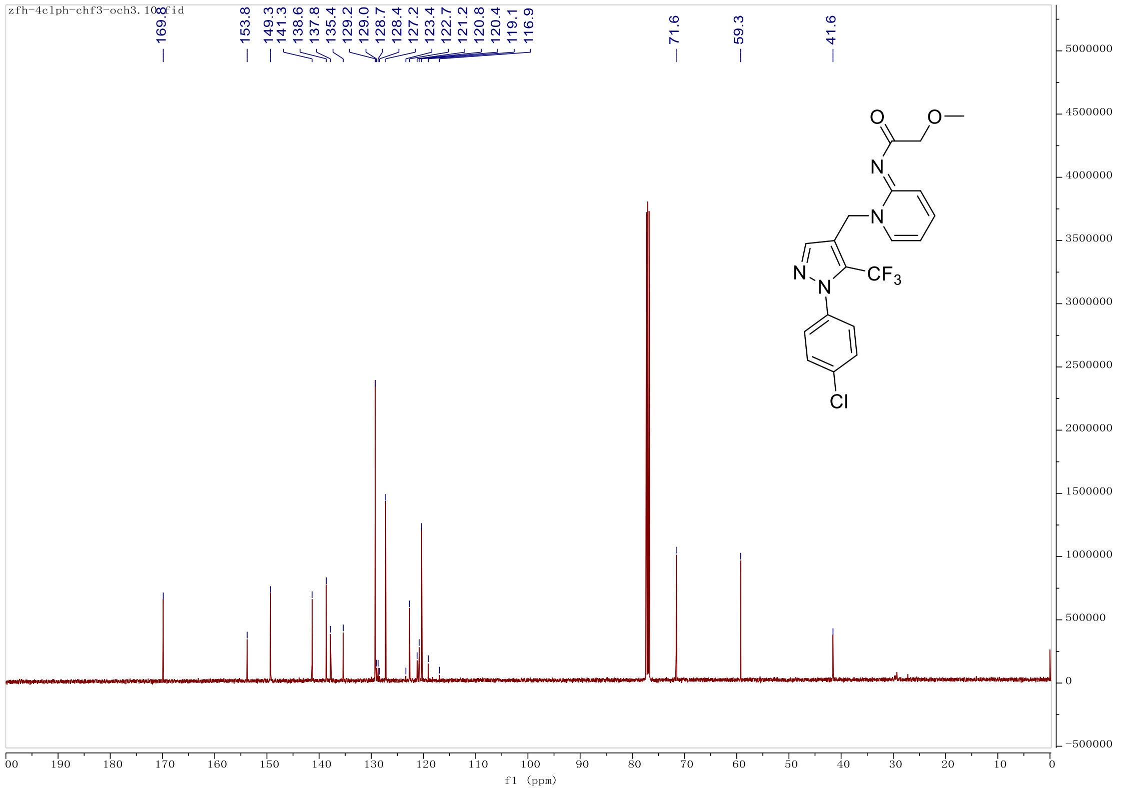

HIGH RESLUTION MASS SPECTROMETRY REPORT

Sample No.	Formula (M)	Ion Formula	Measured m/z	Calc m/z	Diff (ppm)
**B4**	$C_{19}H_{16}ClF_3N_4O_2$	$[M+H]^+$	425.0985	425.0987	-0.47

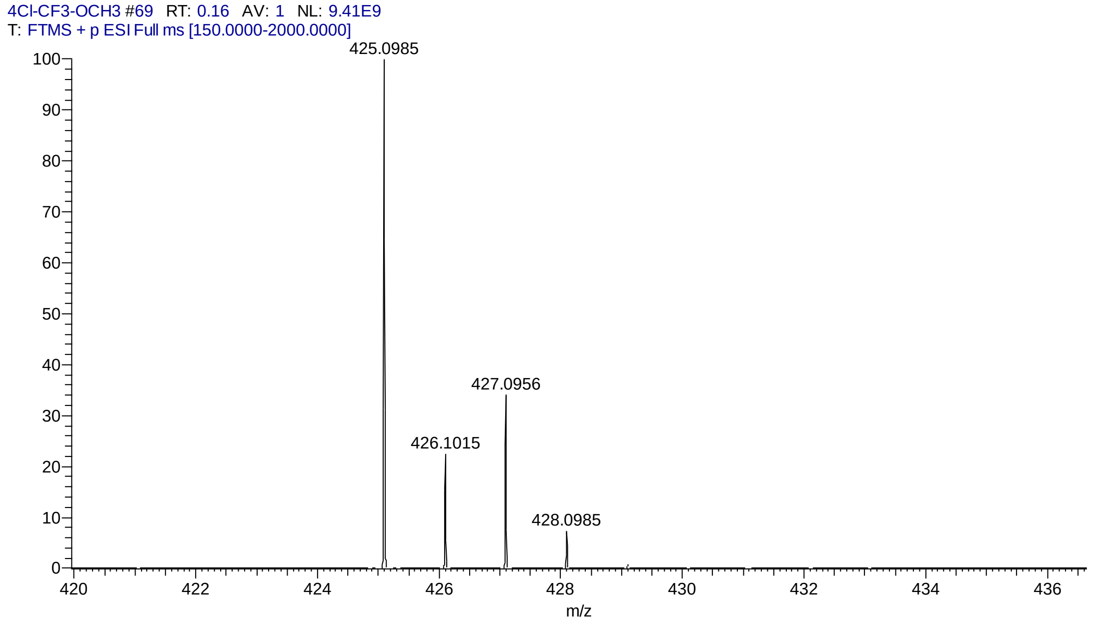

B5

H-NMR

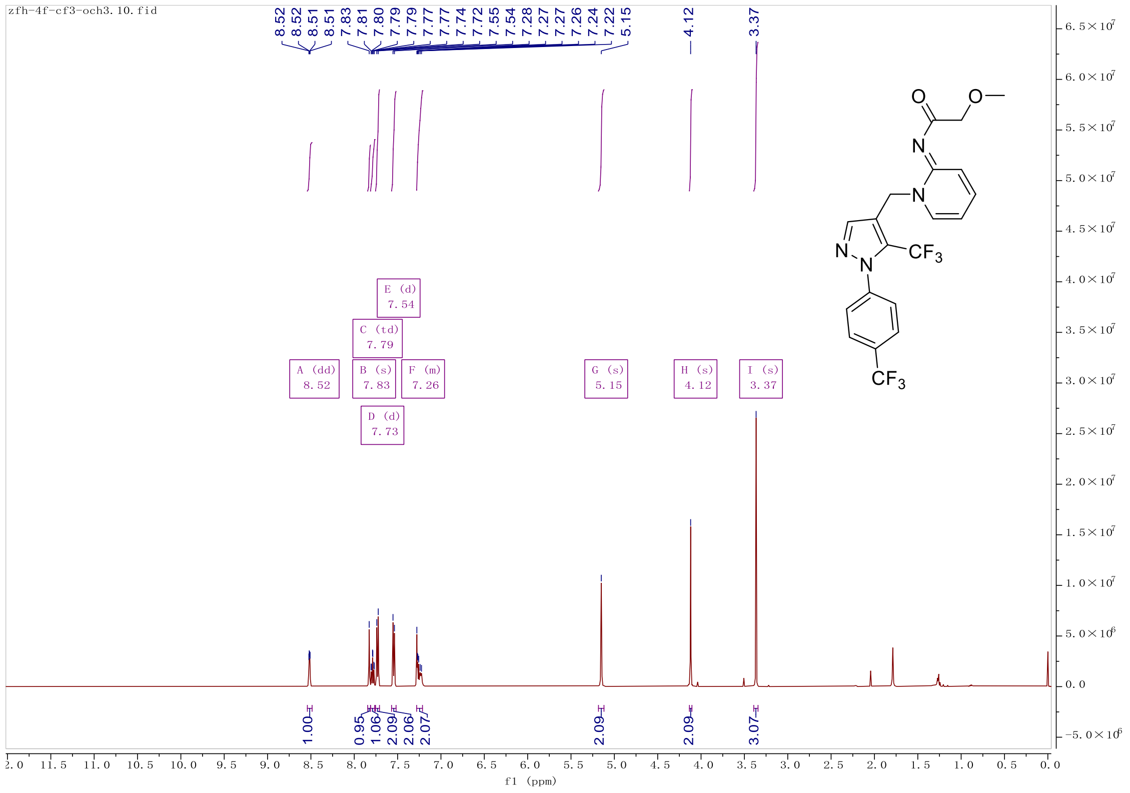

^{13}C-NMR

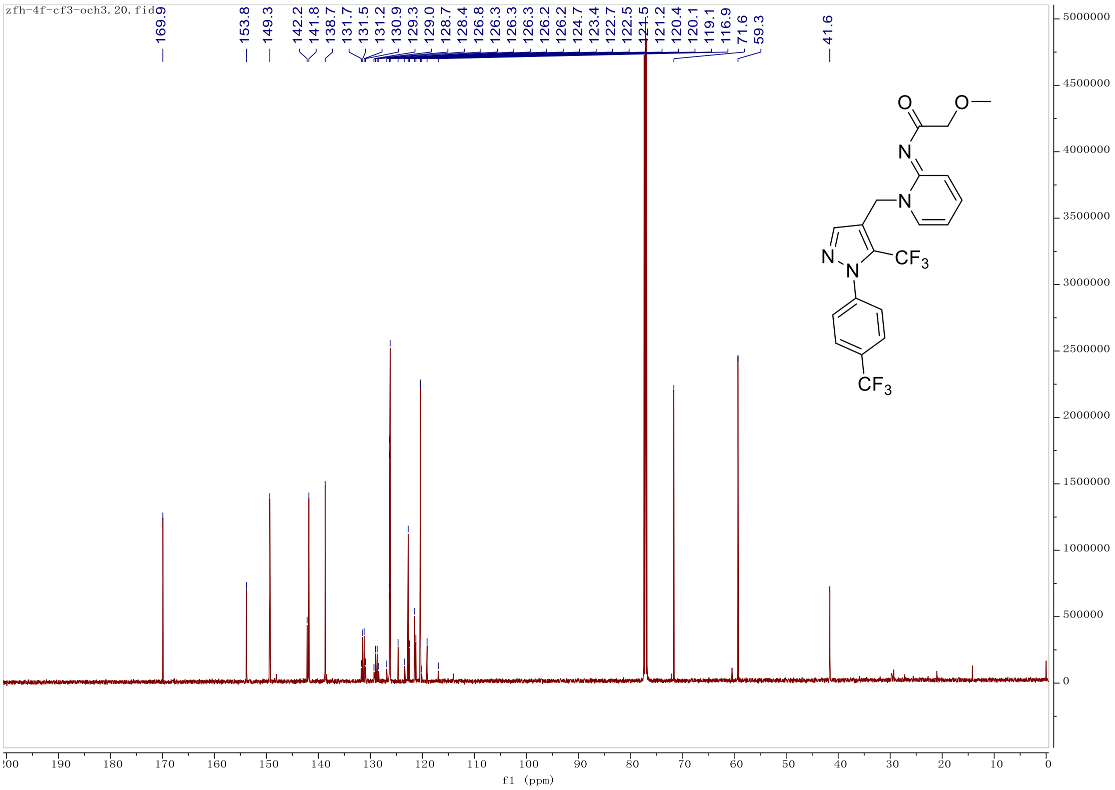

HIGH RESLUTION MASS SPECTROMETRY REPORT

Sample No.	Formula (M)	Ion Formula	Measured m/z	Calc m/z	Diff (ppm)
**B5**	$C_{19}H_{16}F_4N_4O_2$	$[M+H]^+$	409.1280	409.1282	-0.49
	$C_{20}H_{16}F_6N_4O_2$	$[M+H]^+$	459.1238	459.125	-2.61

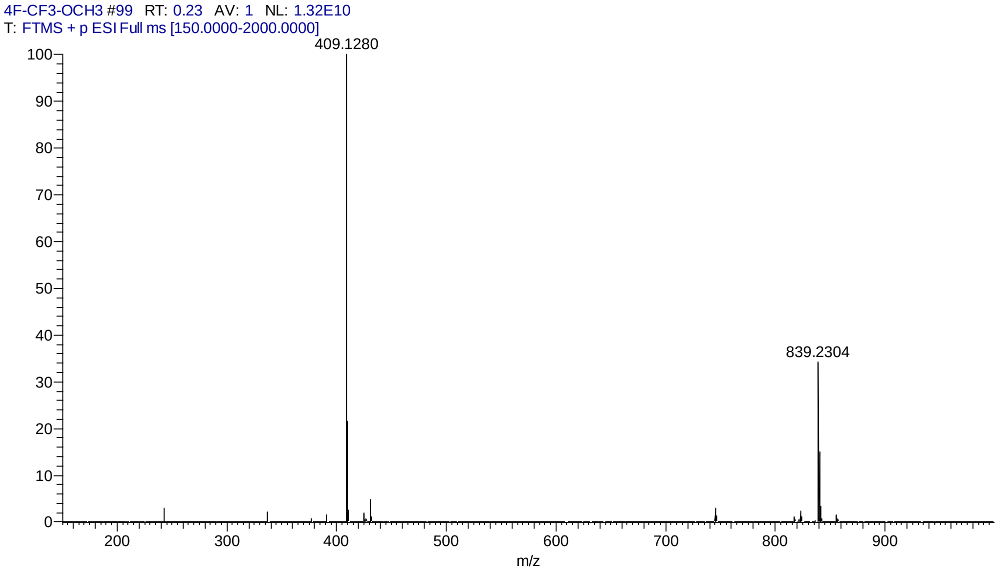

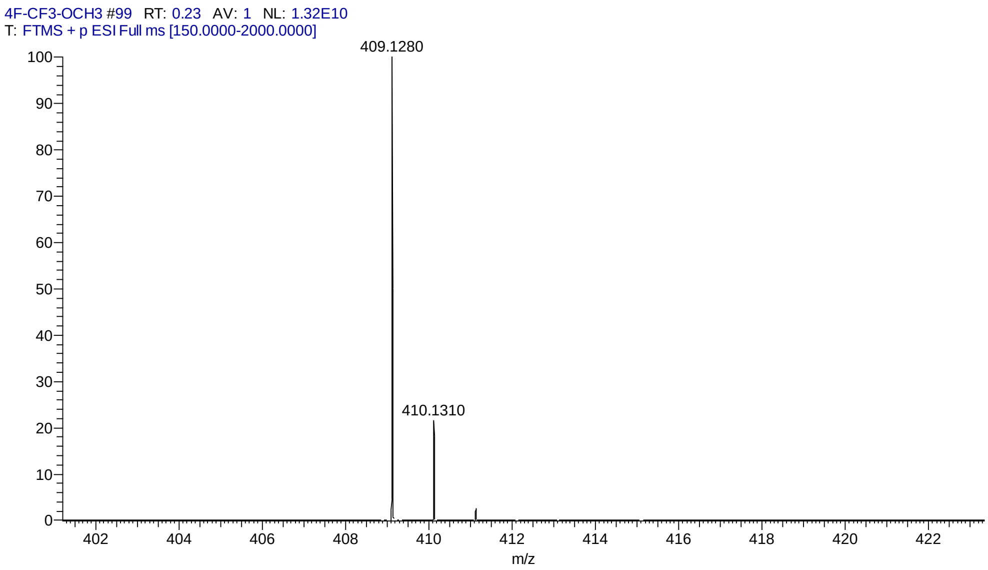
4F-CF3-OCH3 #99 RT: 0.23 AV: 1 NL: 1.32E10
T: FTMS + p ESI Full ms [150.0000-2000.0000]
409.1280
410.1310
m/z

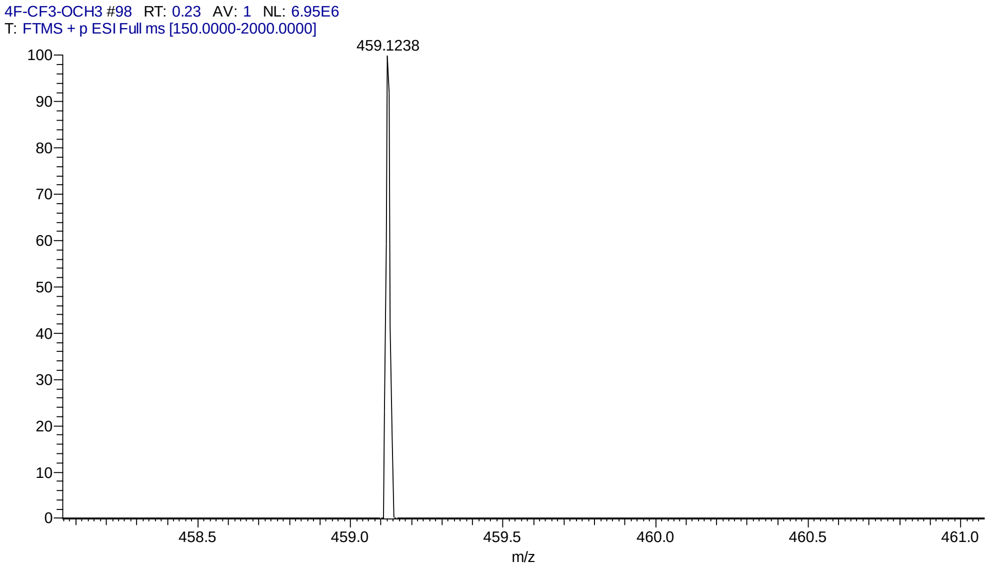
4F-CF3-OCH3 #98 RT: 0.23 AV: 1 NL: 6.95E6
T: FTMS + p ESI Full ms [150.0000-2000.0000]
459.1238
m/z

B6

H-NMR

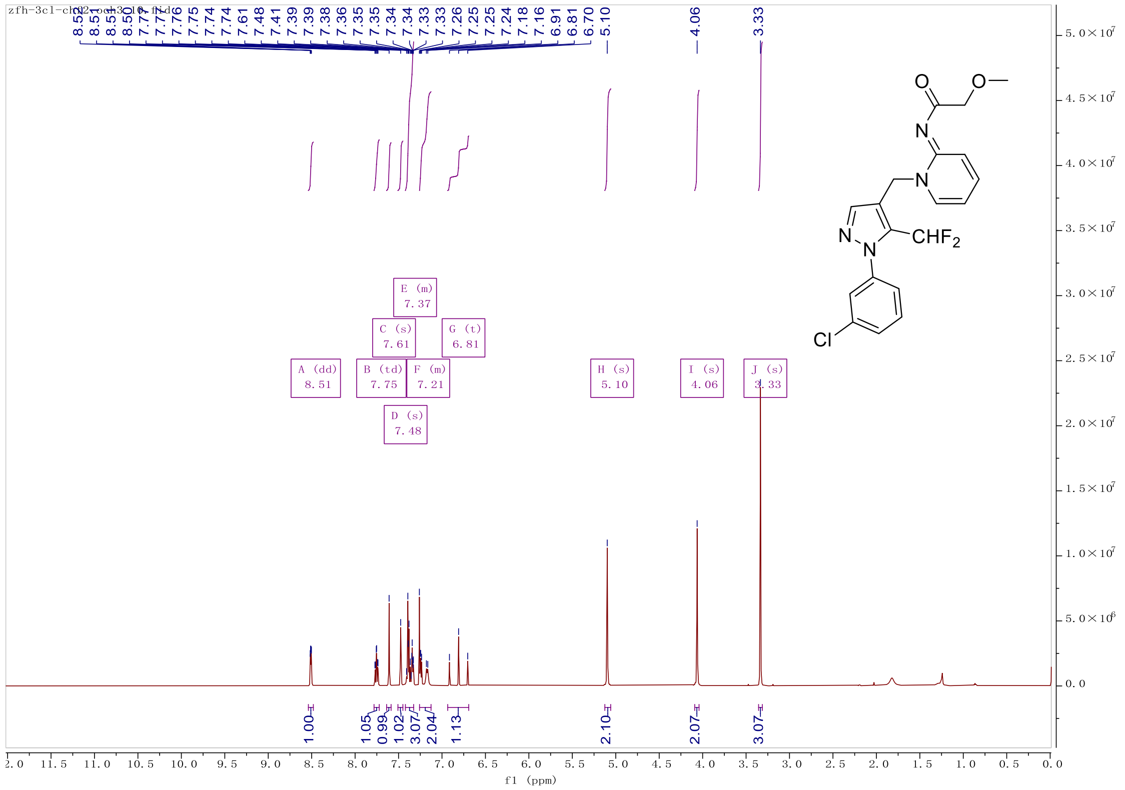

^{13}C-NMR

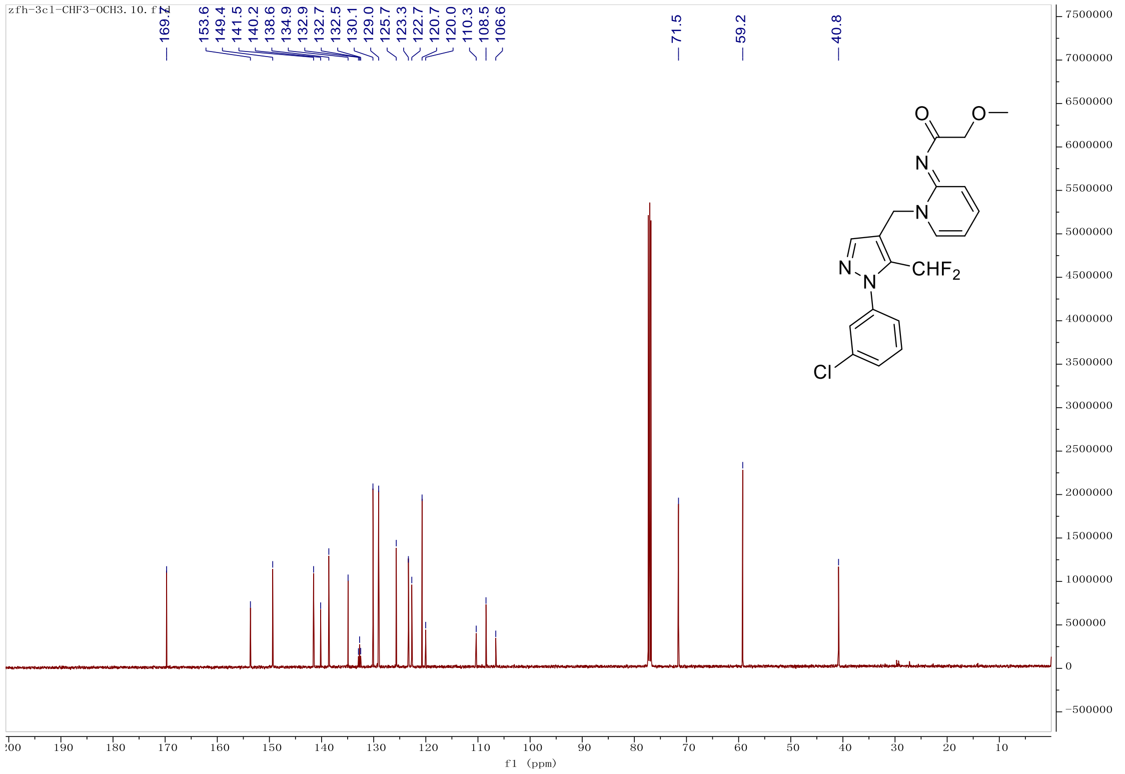

HIGH RESLUTION MASS SPECTROMETRY REPORT

Sample No.	Formula (M)	Ion Formula	Measured m/z	Calc m/z	Diff (ppm)
**B6**	$C_{19}H_{17}ClF_2N_4O_2$	$[M+H]^+$	407.1080	407.1081	-0.25

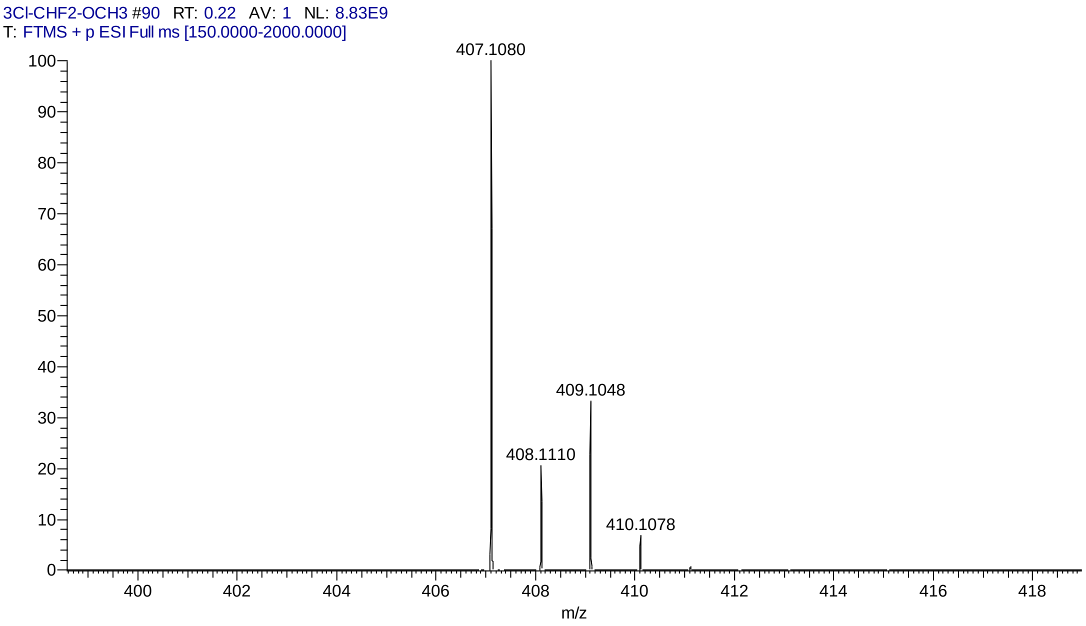

B7

H-NMR

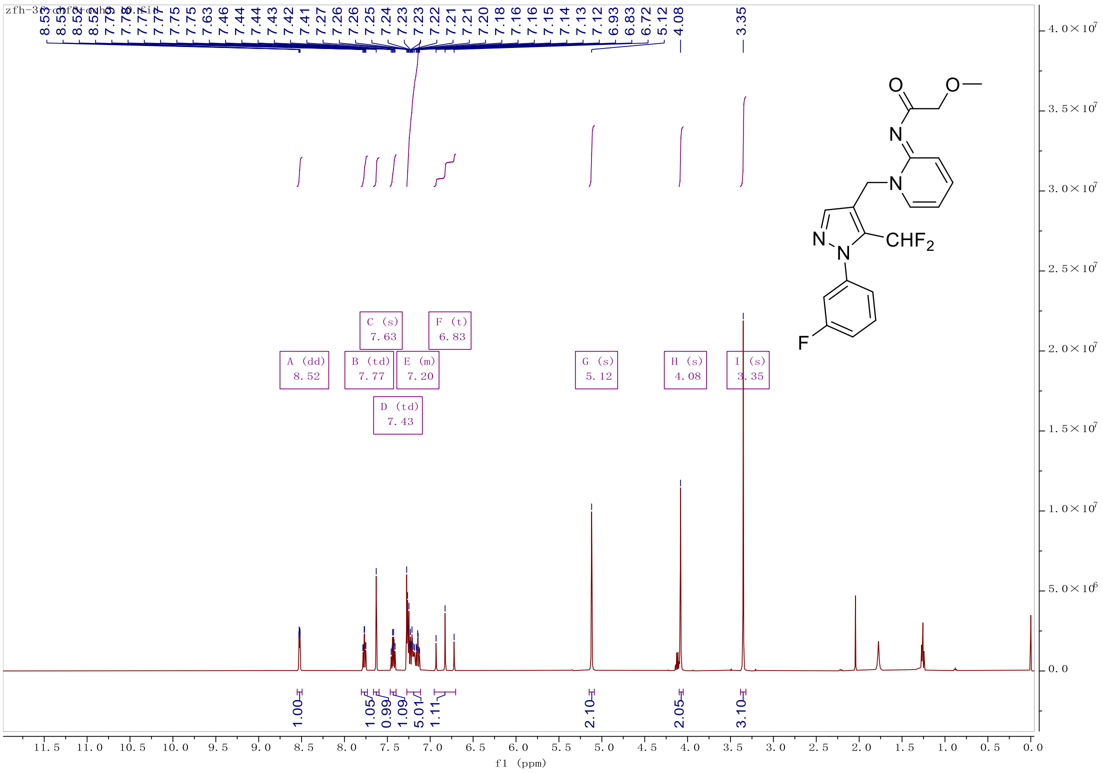

^{13}C-NMR

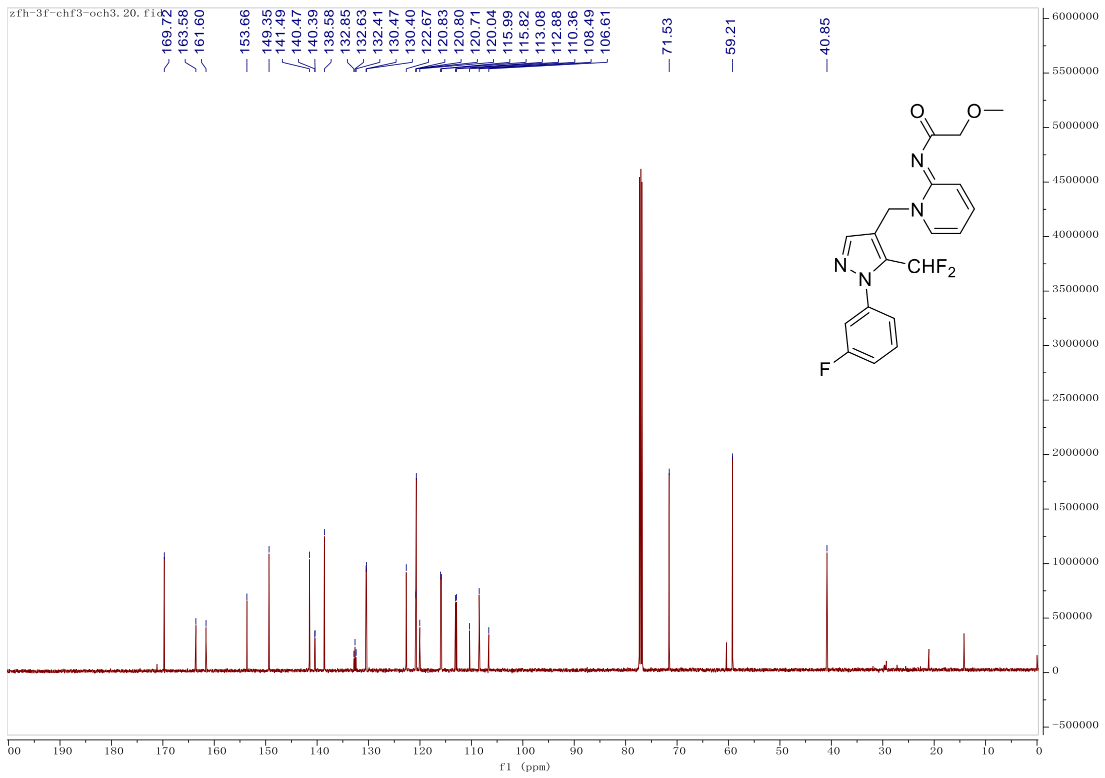

B8

H-NMR

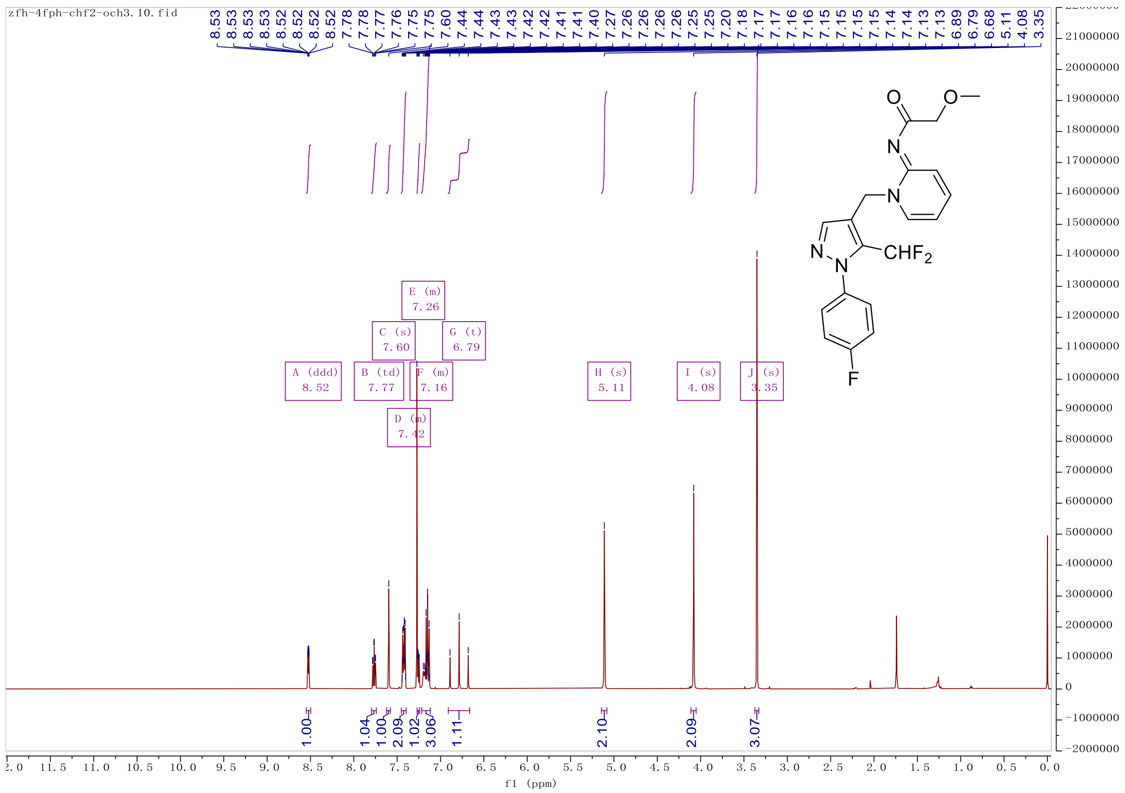

^{13}C-NMR

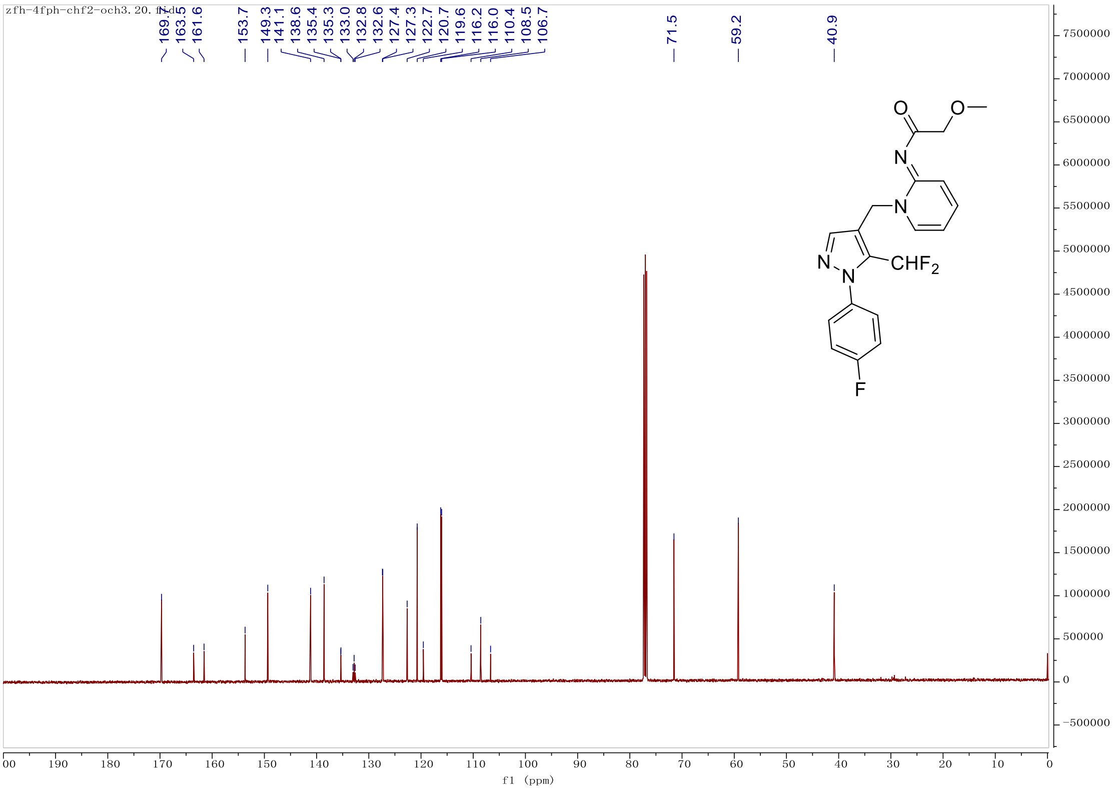

HIGH RESLUTION MASS SPECTROMETRY REPORT

Sample No.	Formula (M)	Ion Formula	Measured m/z	Calc m/z	Diff (ppm)
**B7**	$C_{19}H_{17}F_3N_4O_2$	$[M+H]^+$	391.1375	391.1376	-0.26

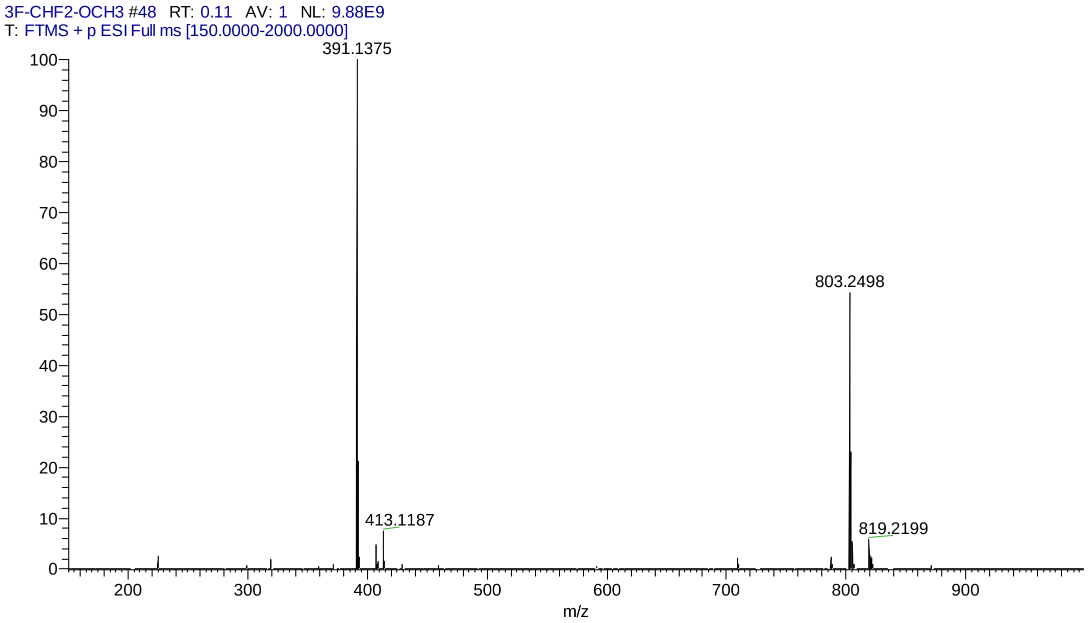

C1

H-NMR

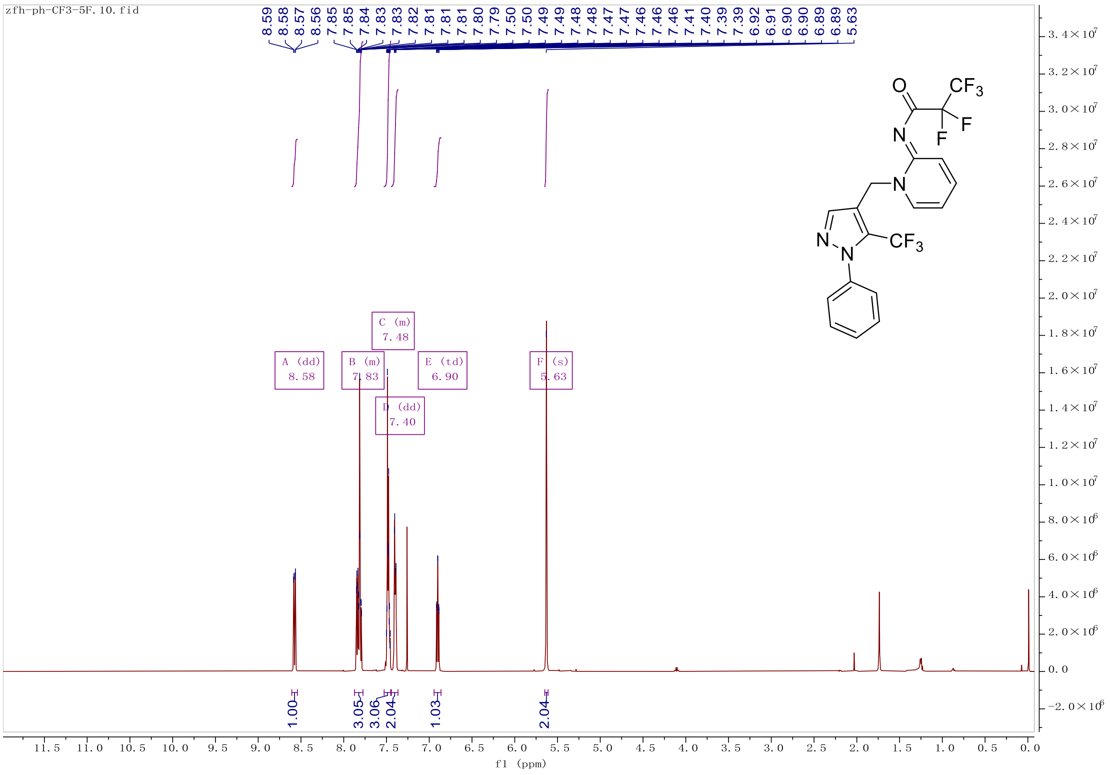

^{13}C-NMR

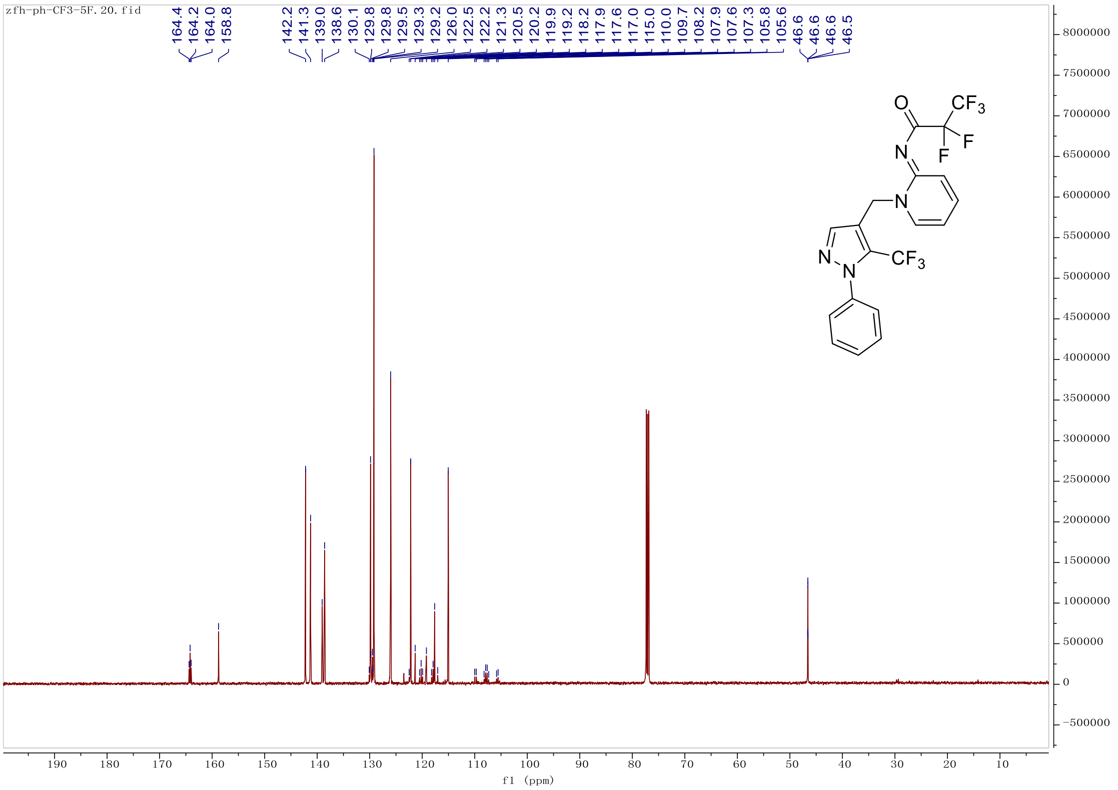

HIGH RESLUTION MASS SPECTROMETRY REPORT

Sample No.	Formula (M)	Ion Formula	Measured m/z	Calc m/z	Diff (ppm)
**C1**	$C_{19}H_{17}F_3N_4O_2$	$[M+H]^+$	391.1375	391.1376	-0.26

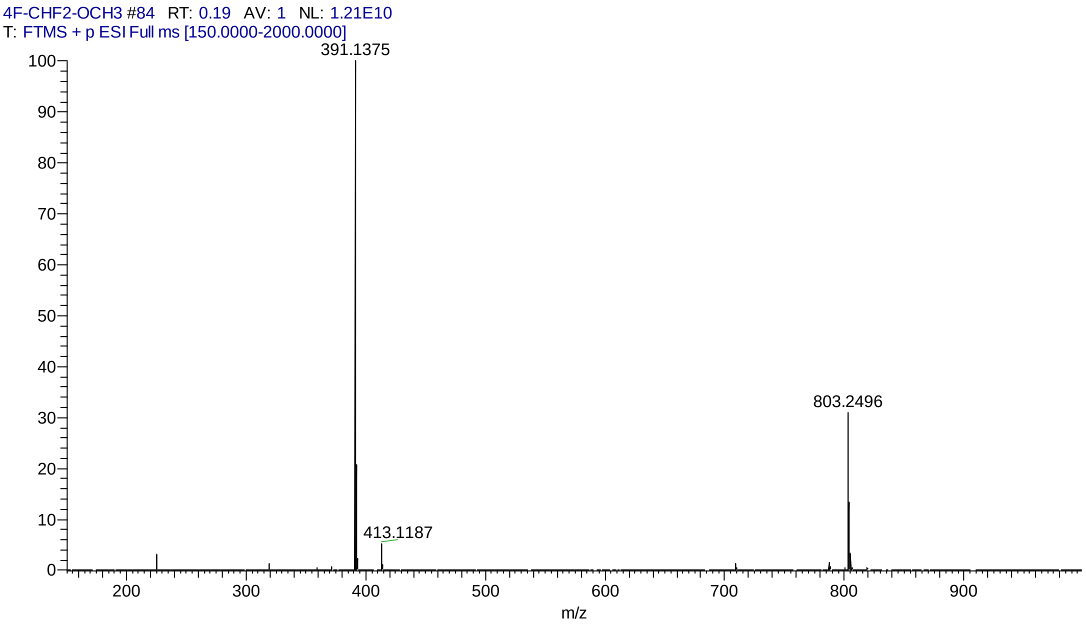

C2

H-NMR

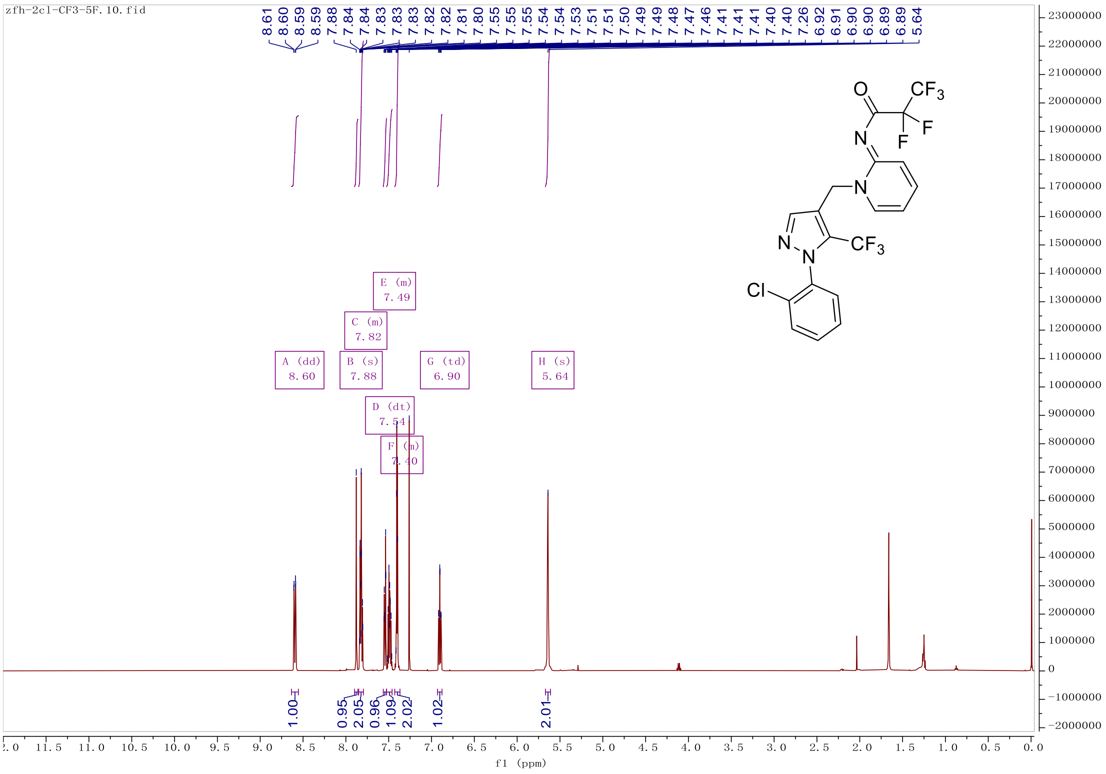

^{13}C-NMR

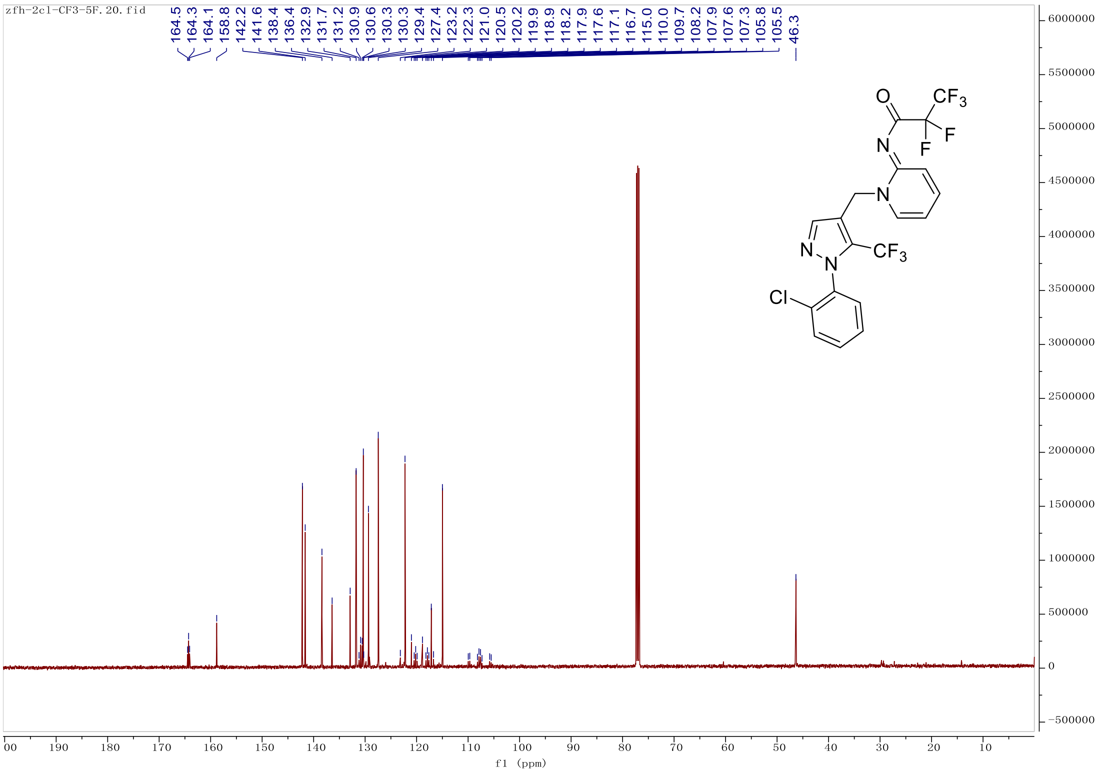

HIGH RESLUTION MASS SPECTROMETRY REPORT

Sample No.	Formula (M)	Ion Formula	Measured m/z	Calc m/z	Diff (ppm)
**C2**	$C_{19}H_{12}F_8N_4O$	$[M+H]^+$	465.0956	465.0956	0

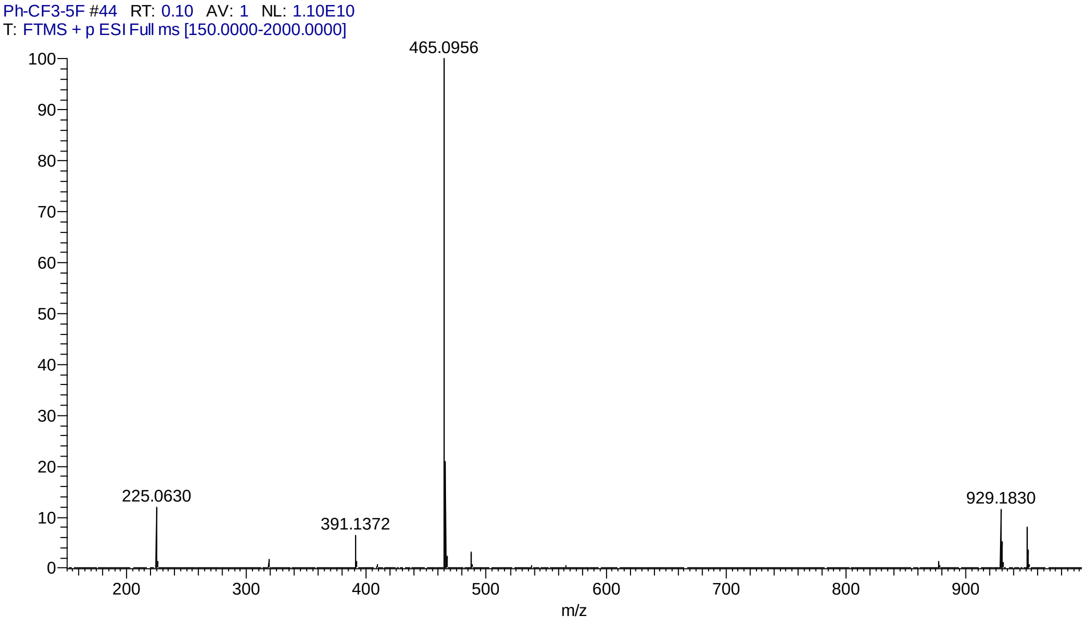

C3

H-NMR

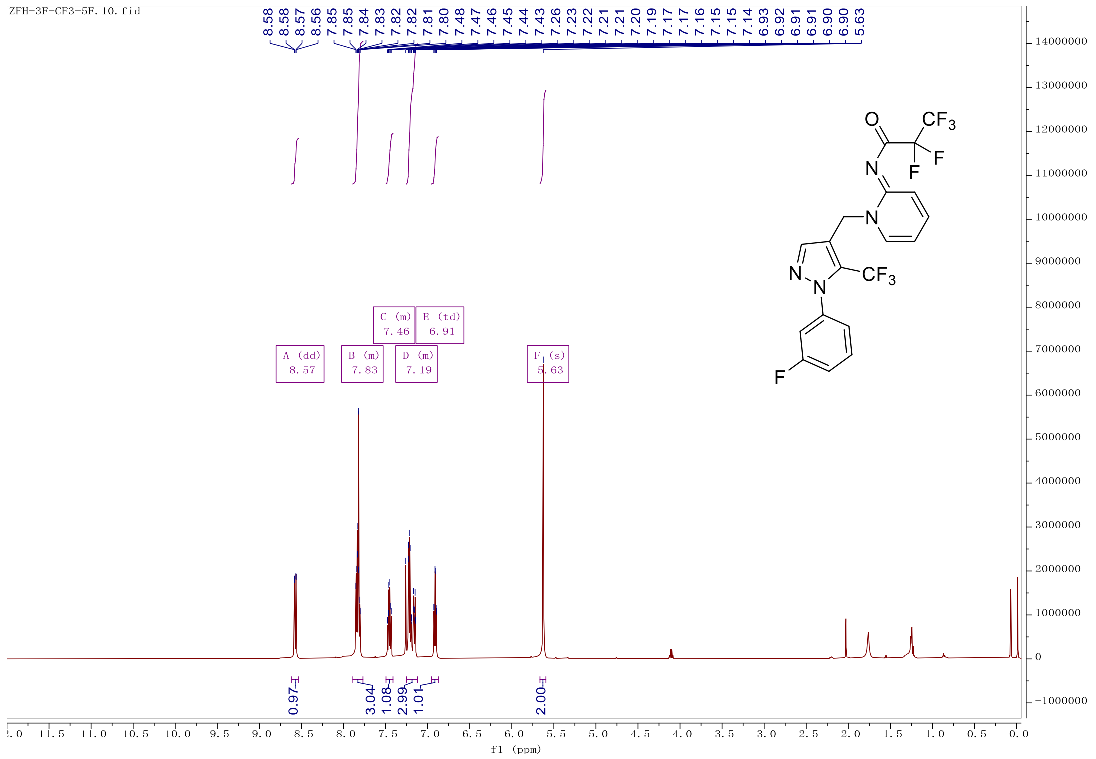

^{13}C-NMR

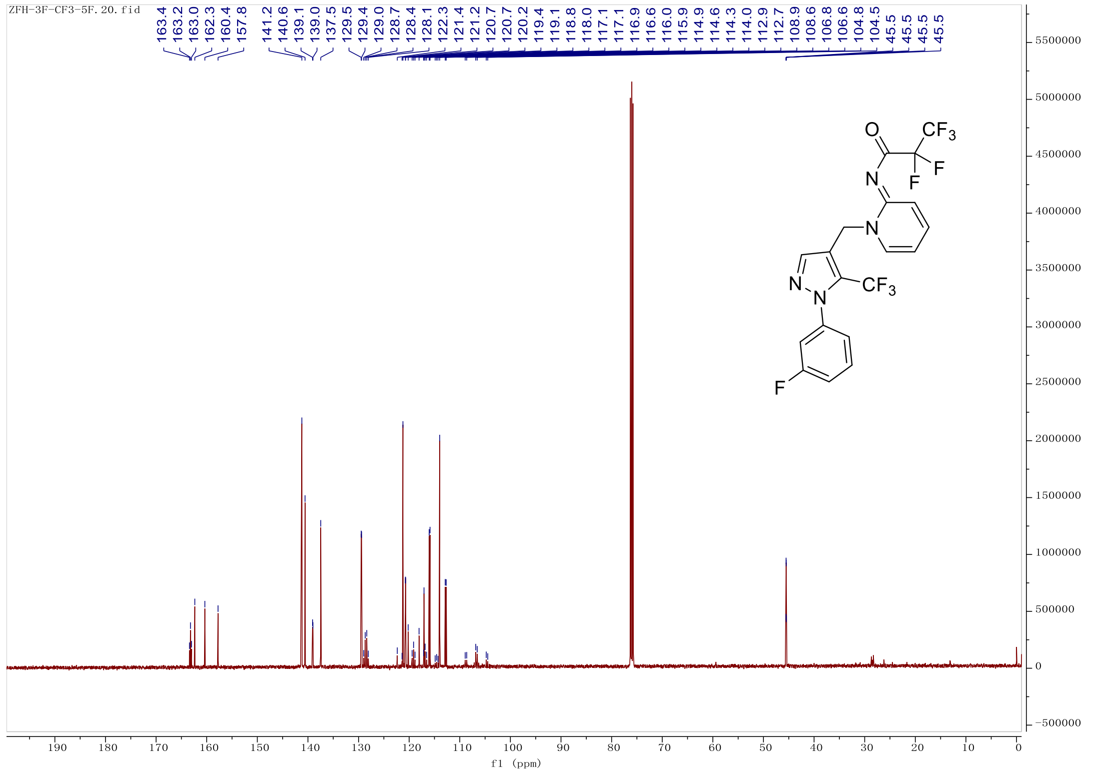

HIGH RESLUTION MASS SPECTROMETRY REPORT

Sample No.	Formula (M)	Ion Formula	Measured m/z	Calc m/z	Diff (ppm)
**C3**	$C_{19}H_{11}ClF_8N_4O$	$[M+H]^+$	499.0565	499.0566	-0.20

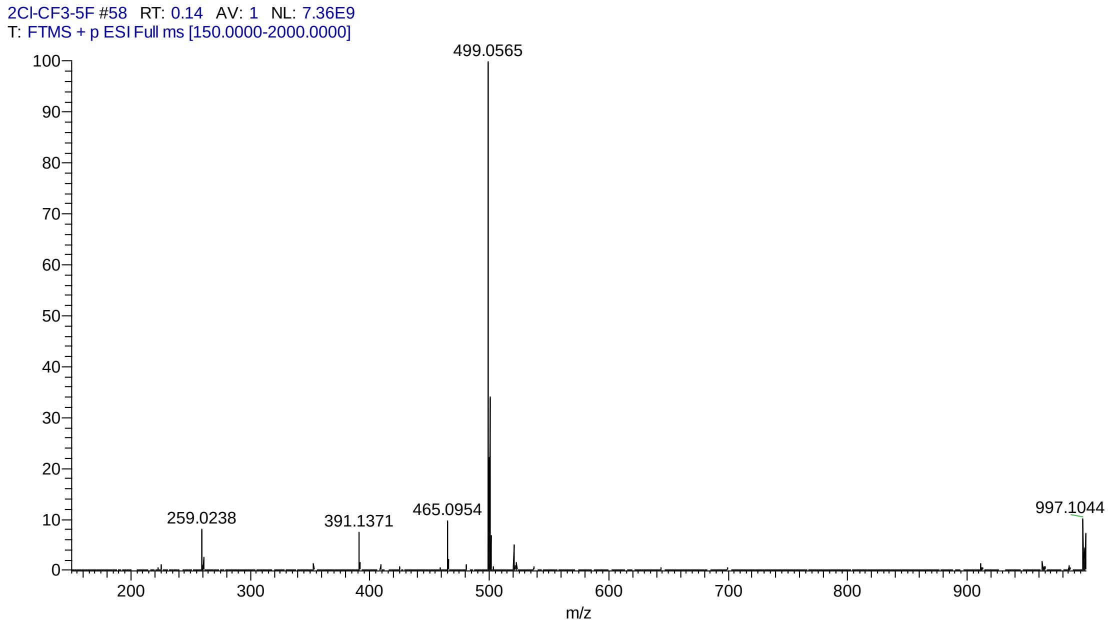

C4

H-NMR

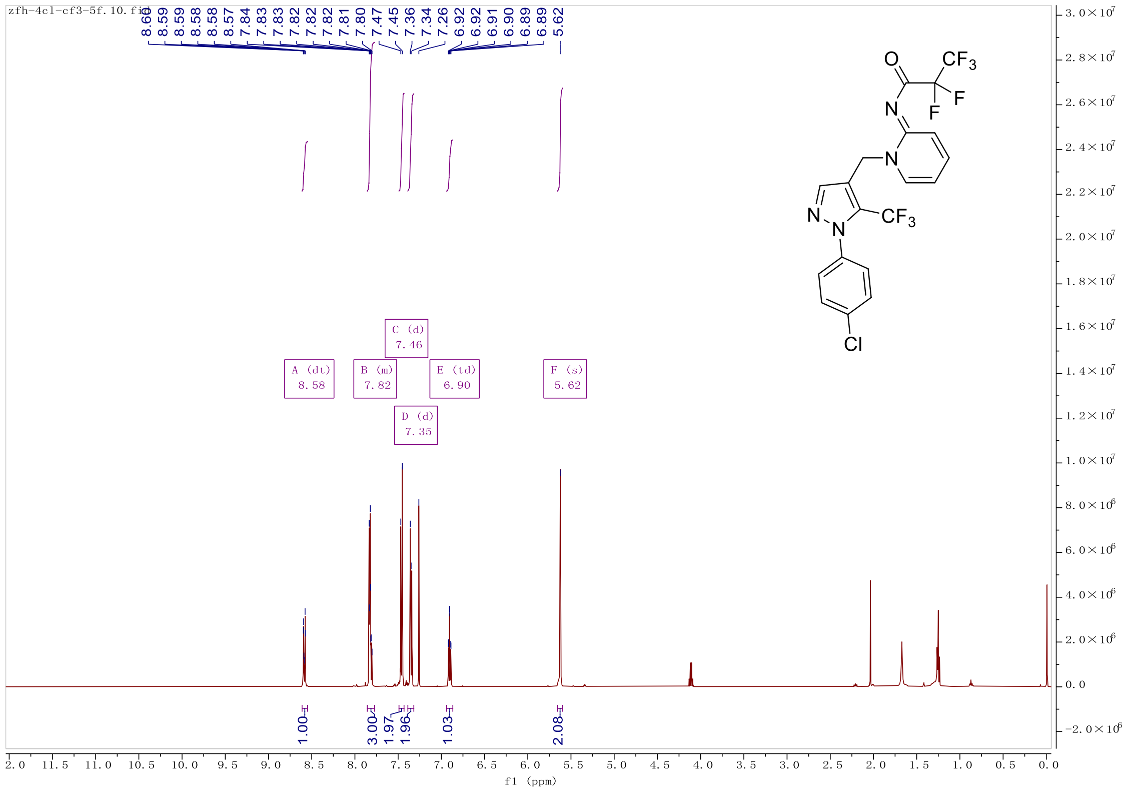

^{13}C-NMR

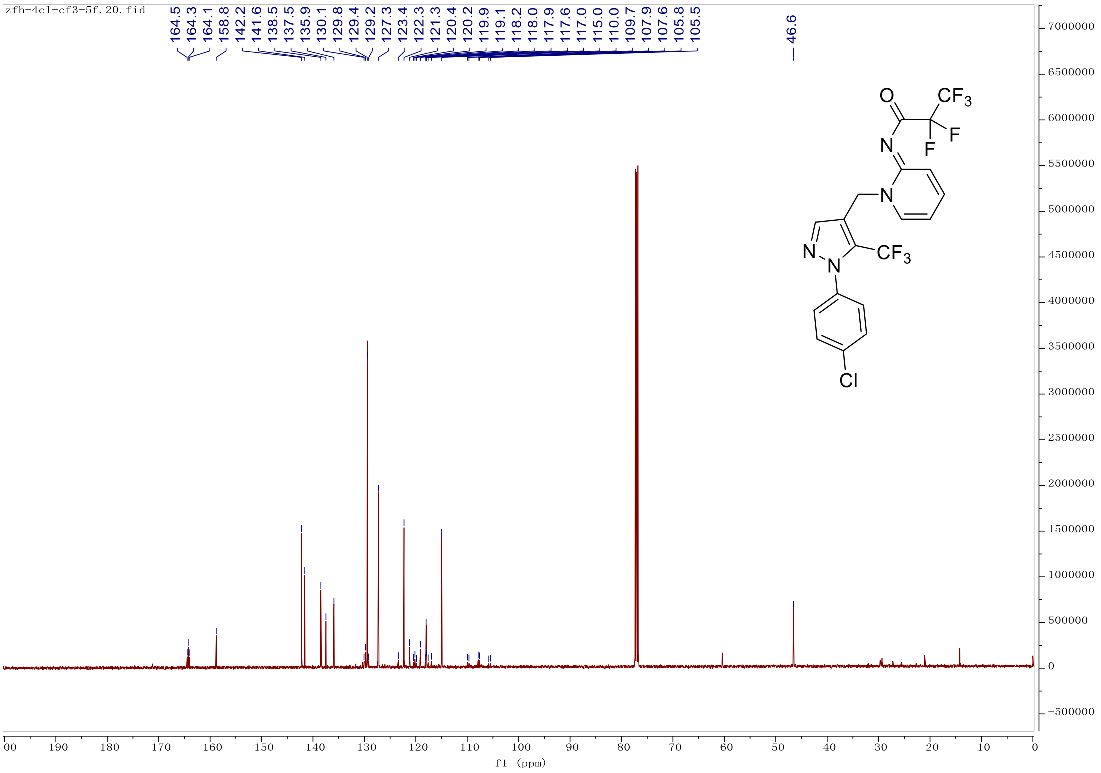

HIGH RESLUTION MASS SPECTROMETRY REPORT

Sample No.	Formula (M)	Ion Formula	Measured m/z	Calc m/z	Diff (ppm)
**C4**	$C_{19}H_{11}F_9N_4O$	$[M+H]^+$	483.0861	483.0862	-0.21

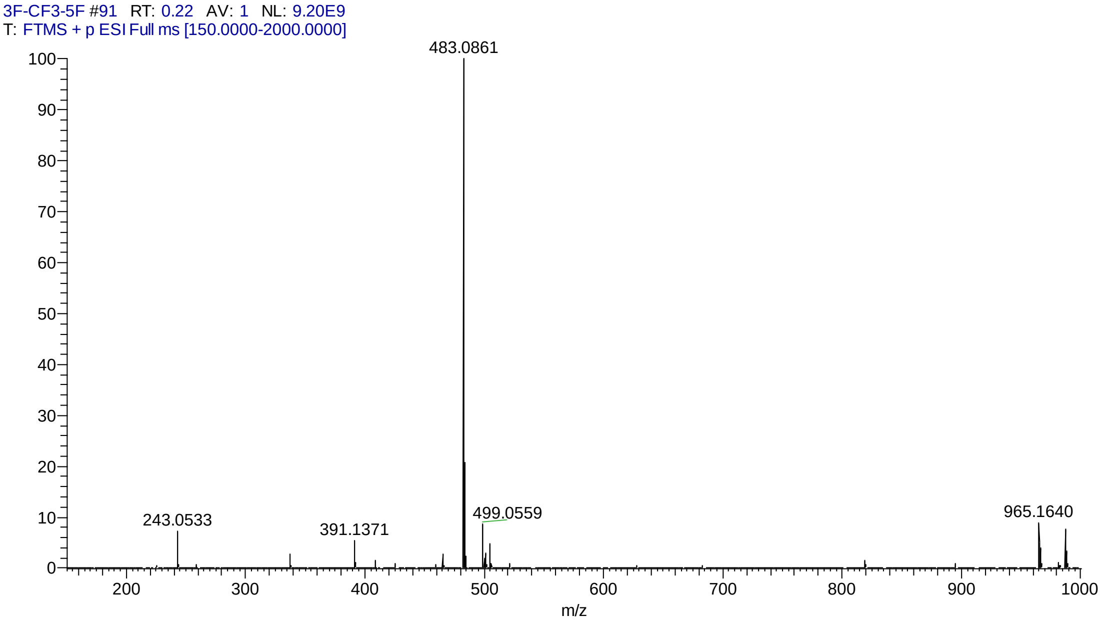

C5

H-NMR

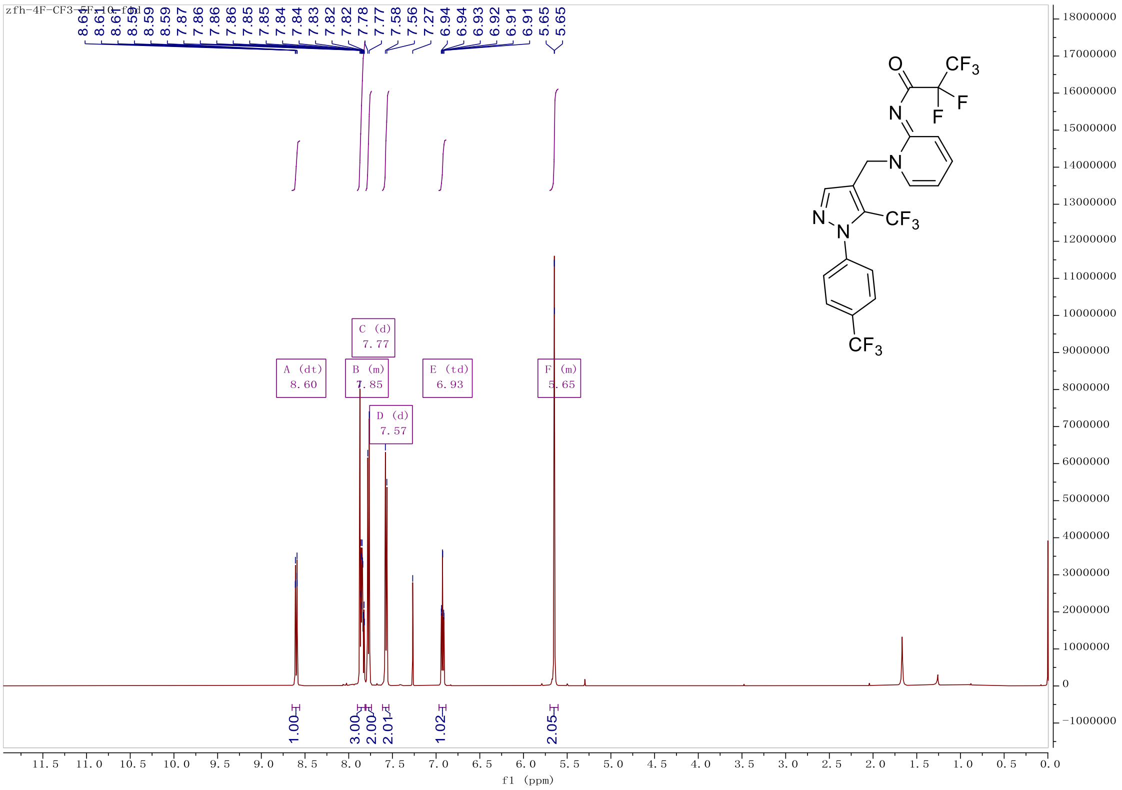

^{13}C-NMR

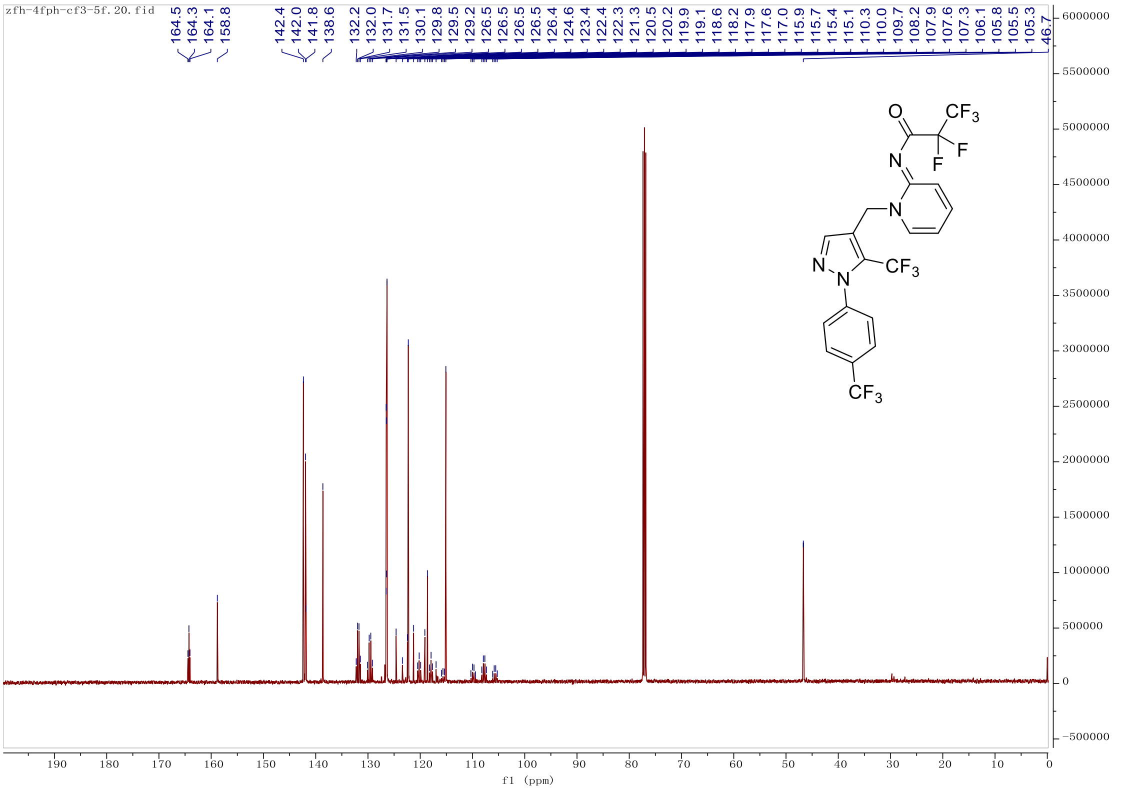

HIGH RESLUTION MASS SPECTROMETRY REPORT

Sample No.	Formula (M)	Ion Formula	Measured m/z	Calc m/z	Diff (ppm)
**C5**	$C_{19}H_{11}ClF_8N_4O$	$[M+H]^+$	499.0565	499.0566	-0.20

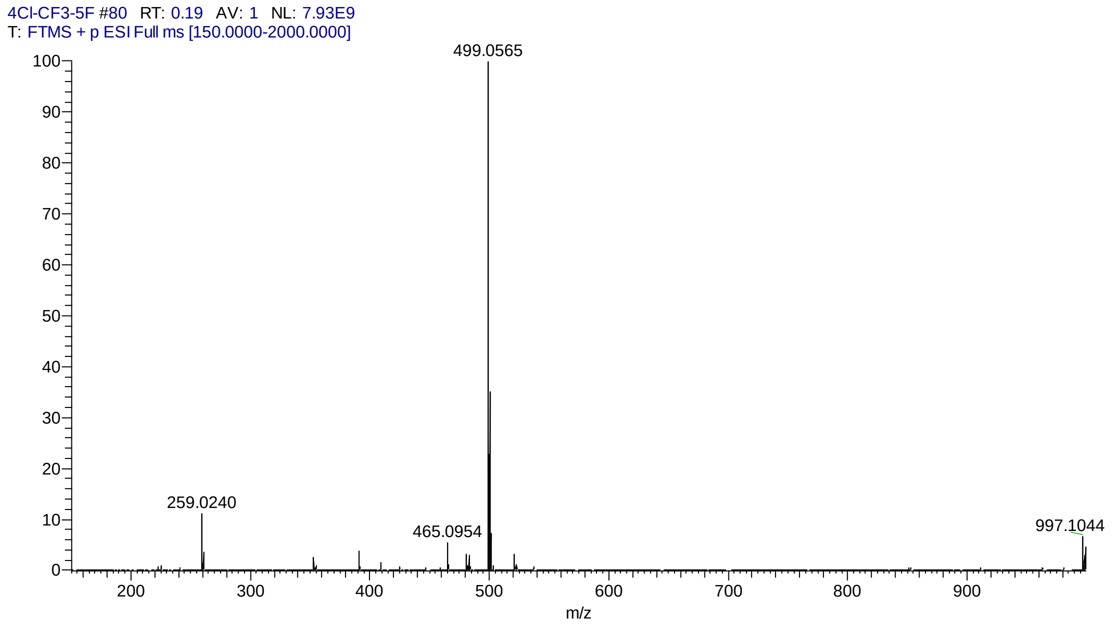

C6

H-NMR

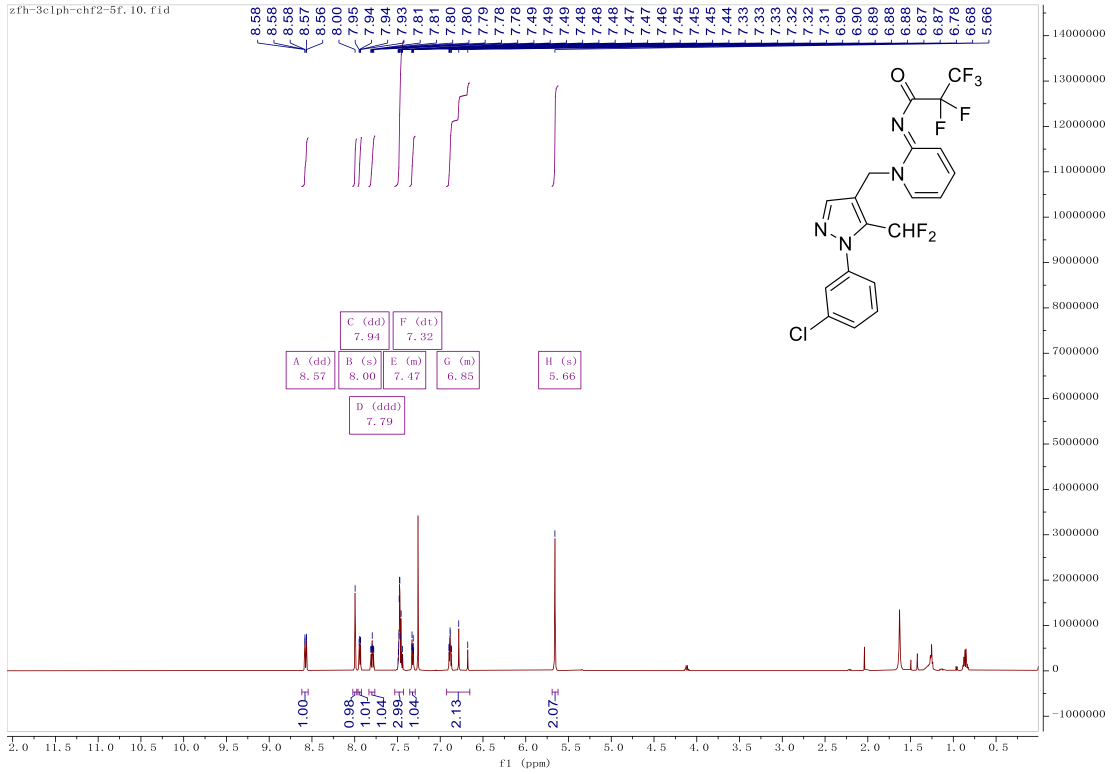

^{13}C-NMR

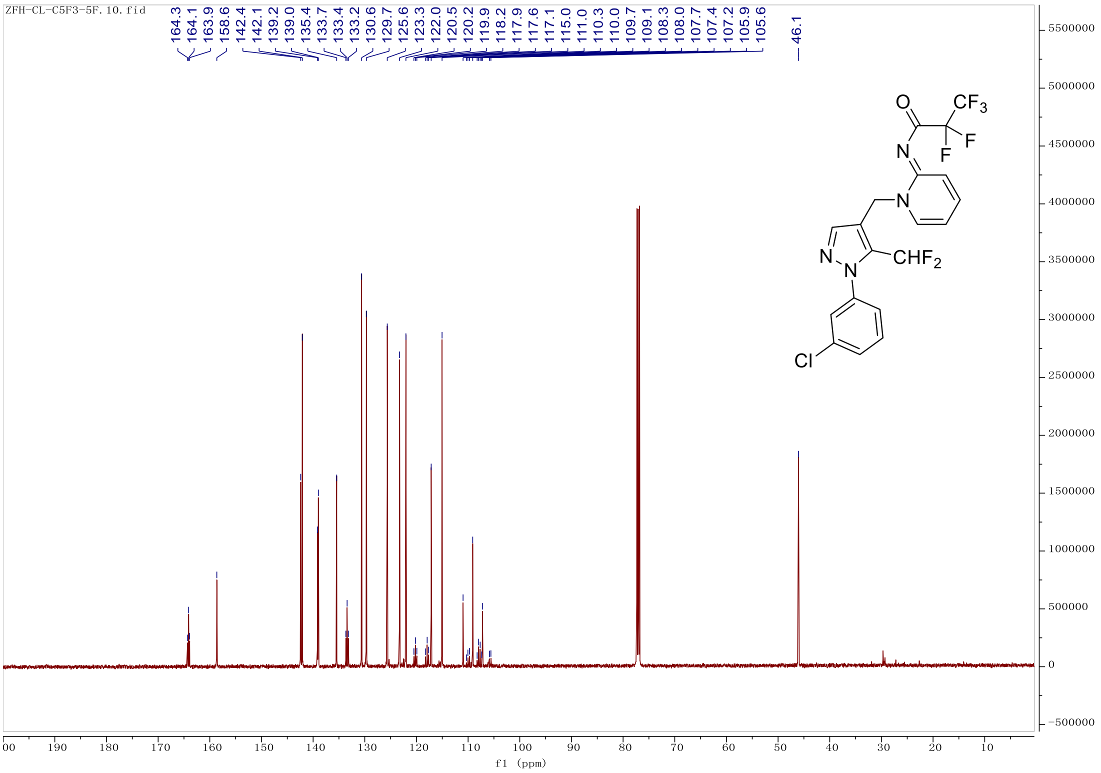

HIGH RESLUTION MASS SPECTROMETRY REPORT

Sample No.	Formula (M)	Ion Formula	Measured m/z	Calc m/z	Diff (ppm)
**C6**	$C_{19}H_{12}ClF_7N_4O$	$[M+H]^+$	481.0661	481.0661	0

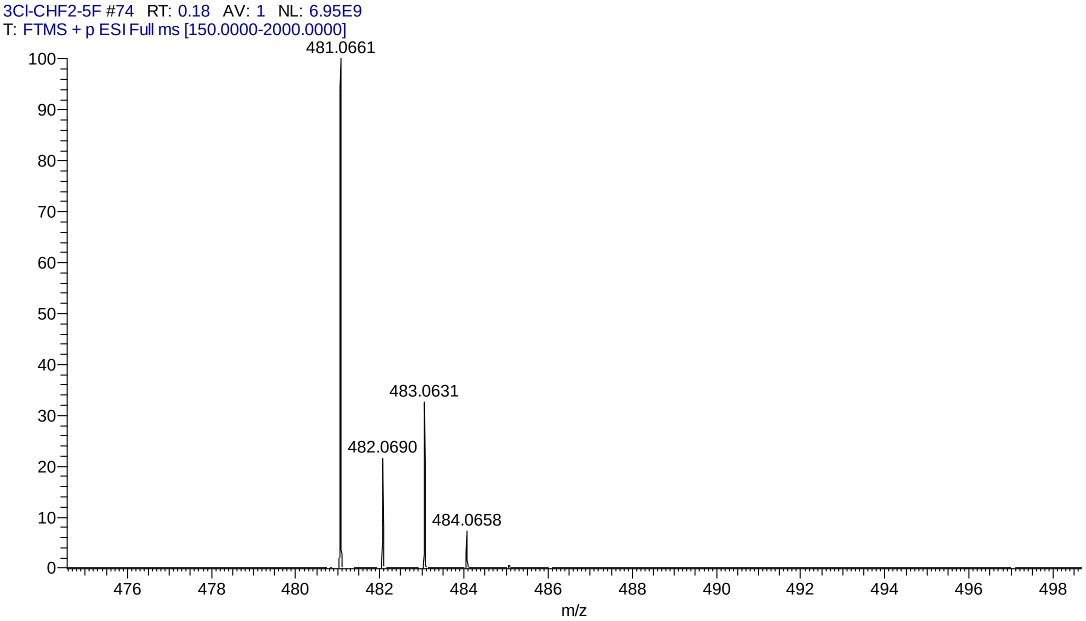

C7

H-NMR

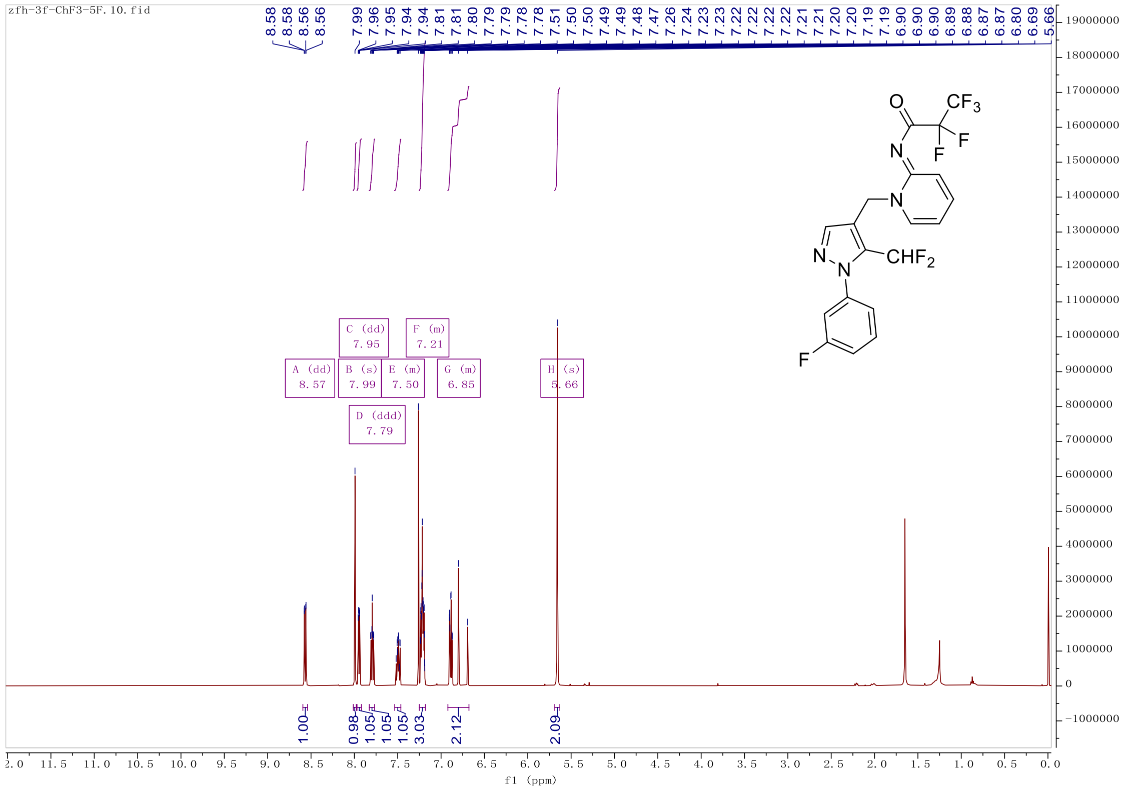

^{13}C-NMR

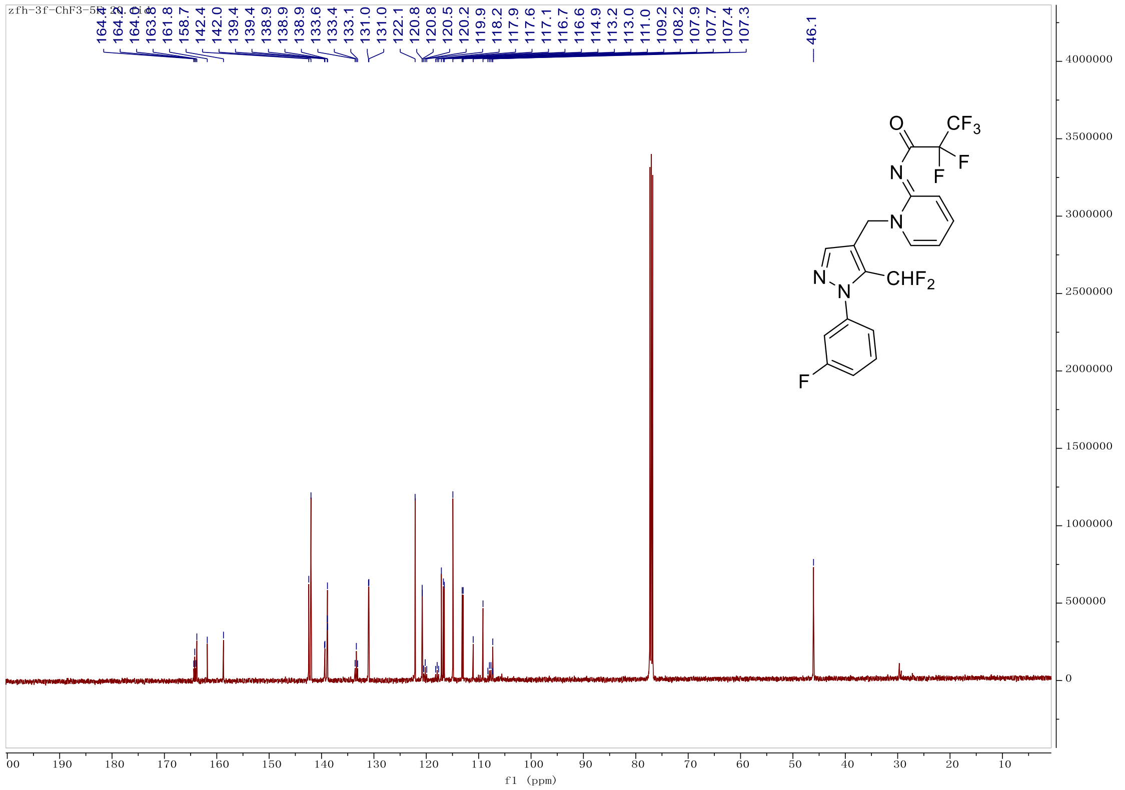

HIGH RESLUTION MASS SPECTROMETRY REPORT

Sample No.	Formula (M)	Ion Formula	Measured m/z	Calc m/z	Diff (ppm)
**C7**	$C_{19}H_{12}F_8N_4O$	$[M+H]^+$	465.0955	465.0956	-0.22

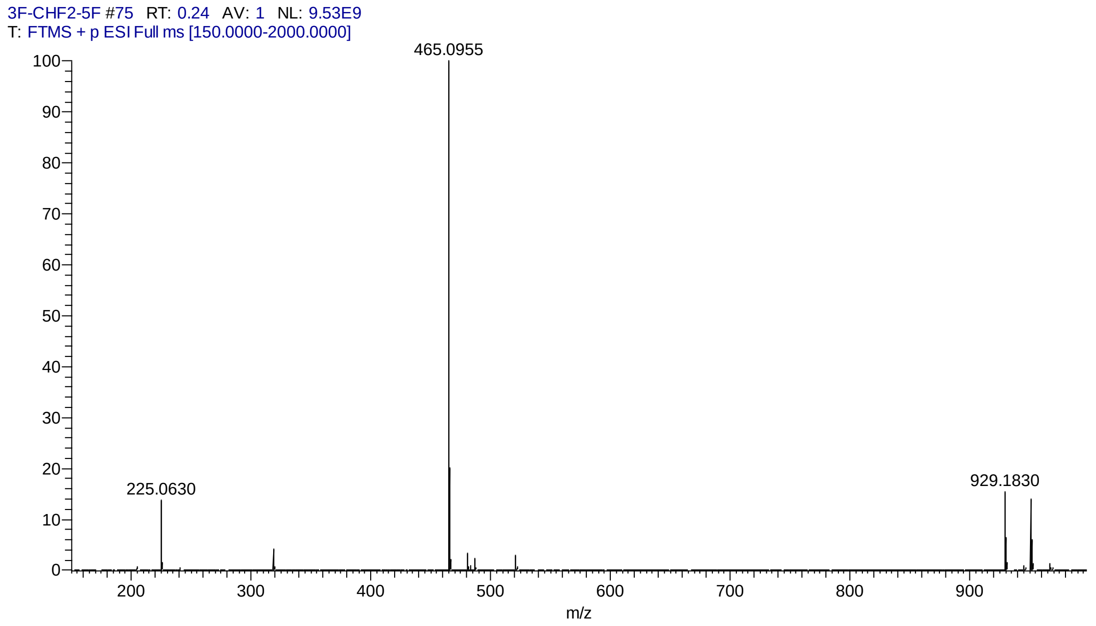

C8

H-NMR

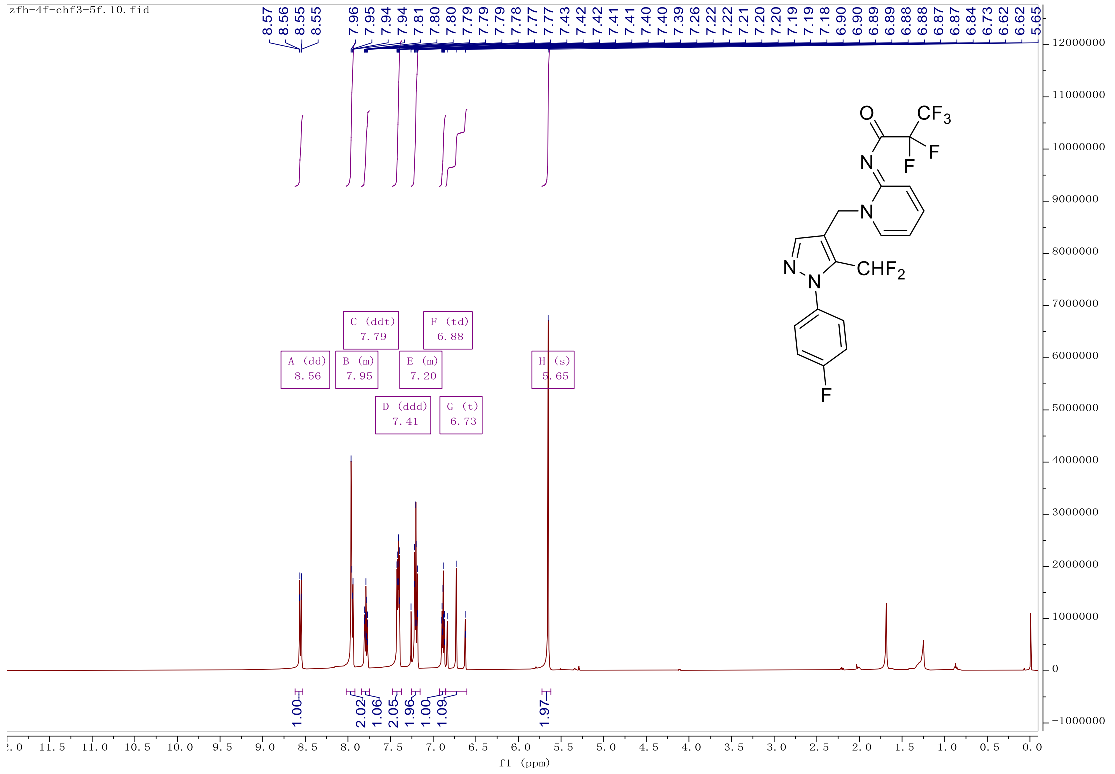

^{13}C-NMR

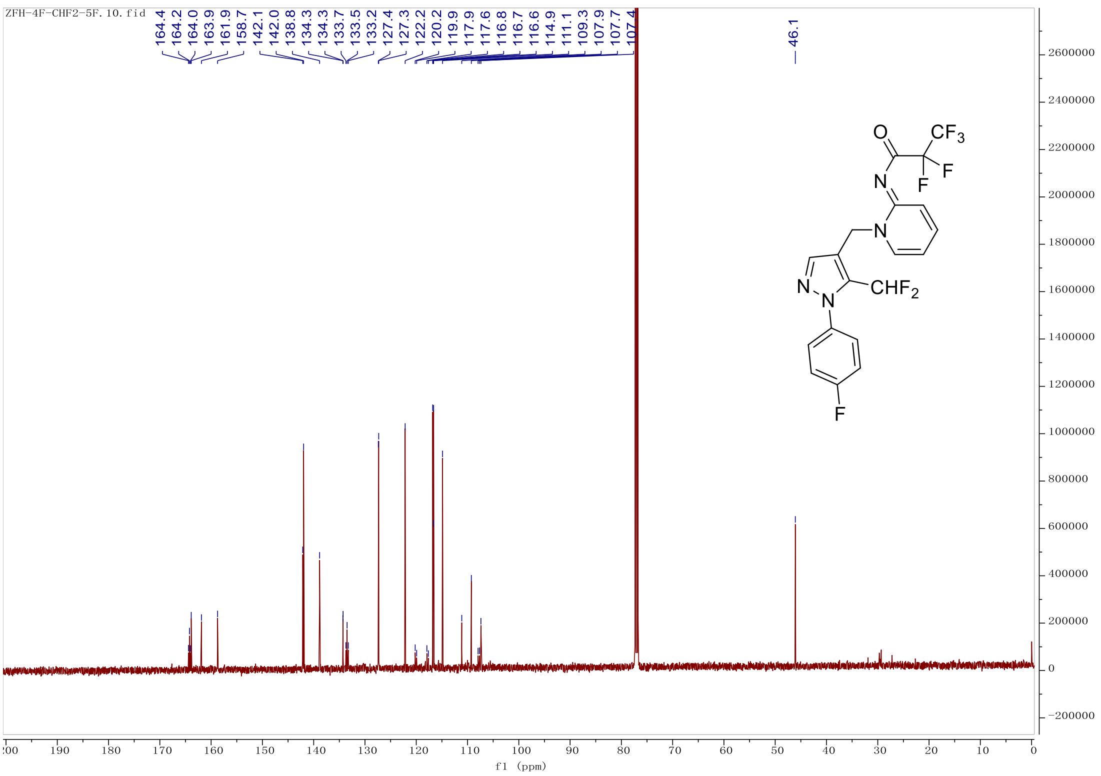

HIGH RESLUTION MASS SPECTROMETRY REPORT

Sample No.	Formula (M)	Ion Formula	Measured m/z	Calc m/z	Diff (ppm)
**C8**	$C_{19}H_{12}F_8N_4O$	$[M+H]^+$	465.0956	465.0956	0

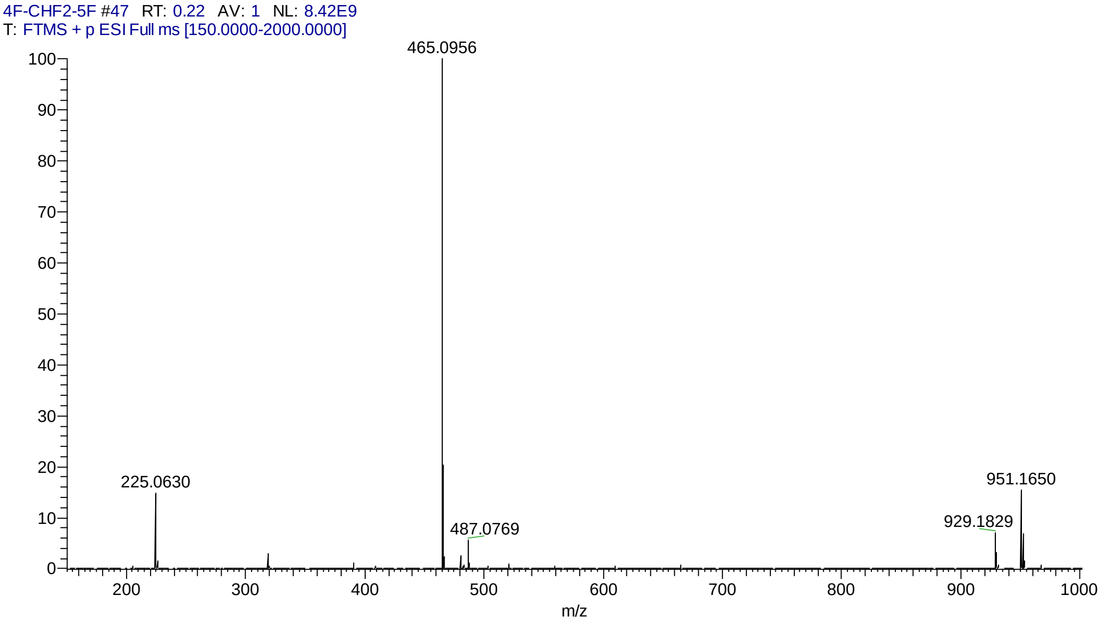

D1

H-NMR

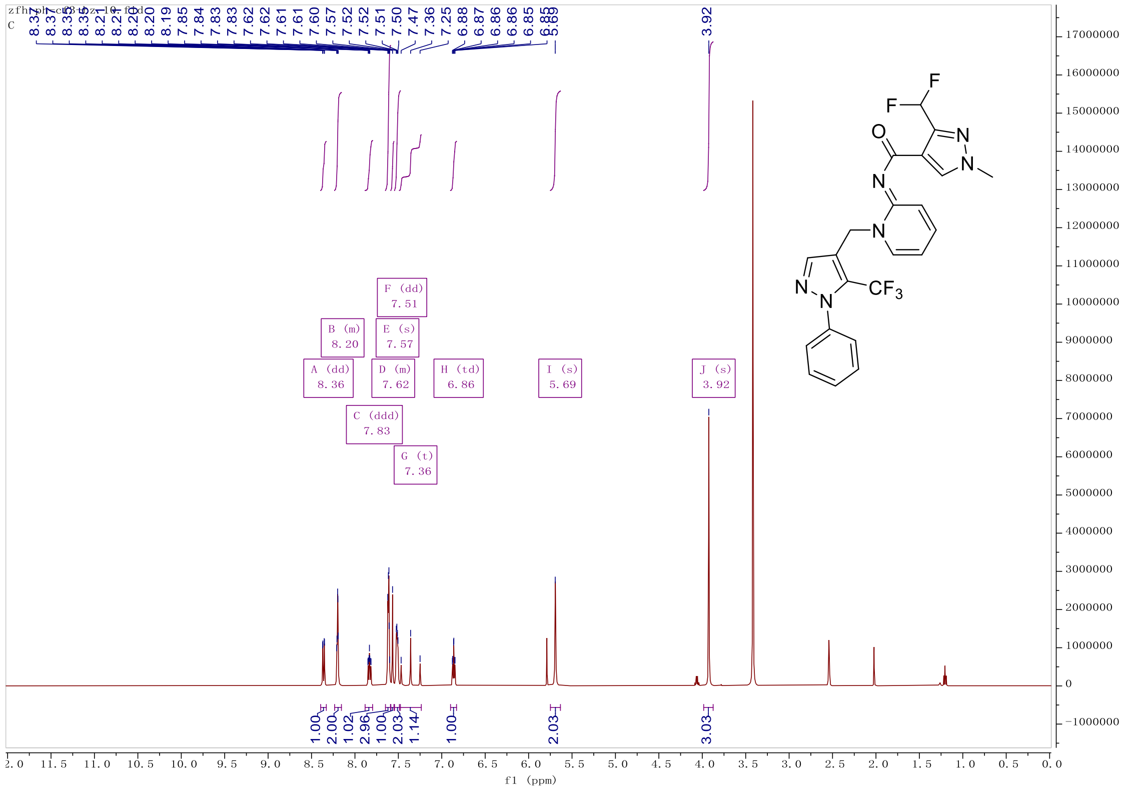

^{13}C-NMR

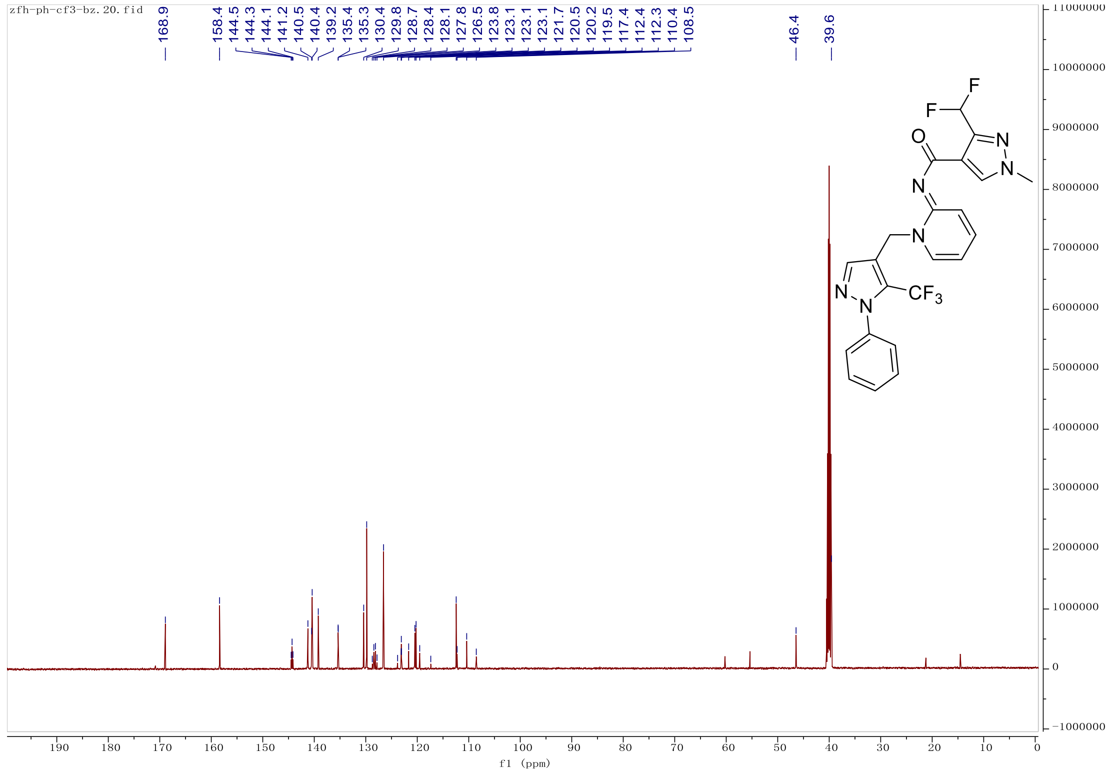

HIGH RESLUTION MASS SPECTROMETRY REPORT

Sample No.	Formula (M)	Ion Formula	Measured m/z	Calc m/z	Diff (ppm)
**D1**	$C_{22}H_{17}F_5N_6O$	$[M+H]^+$	477.1457	477.1457	0

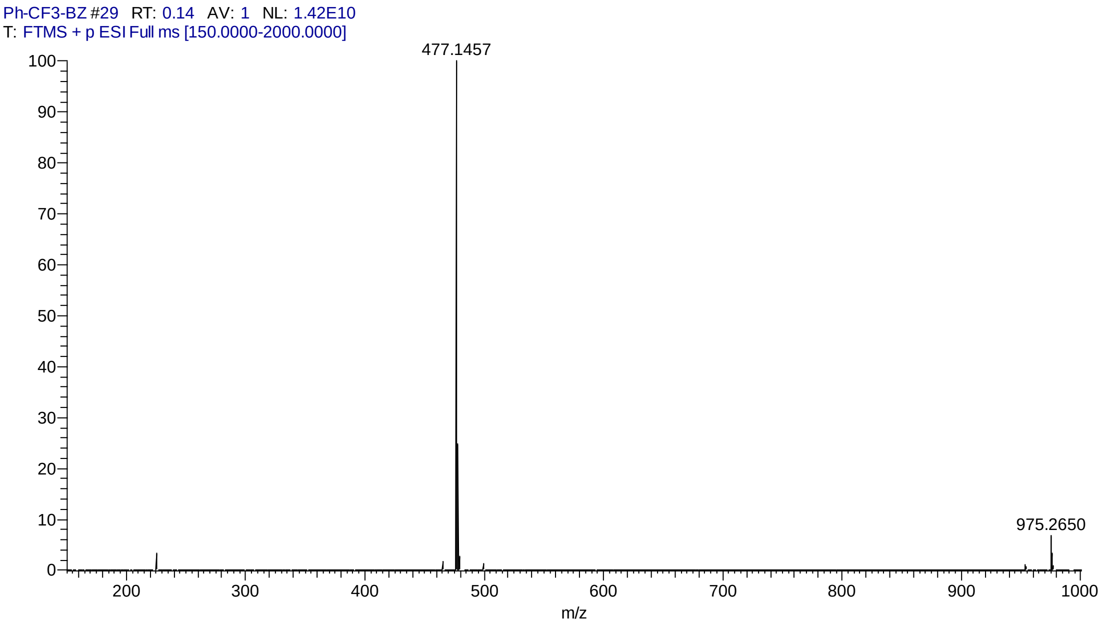

D2

H-NMR

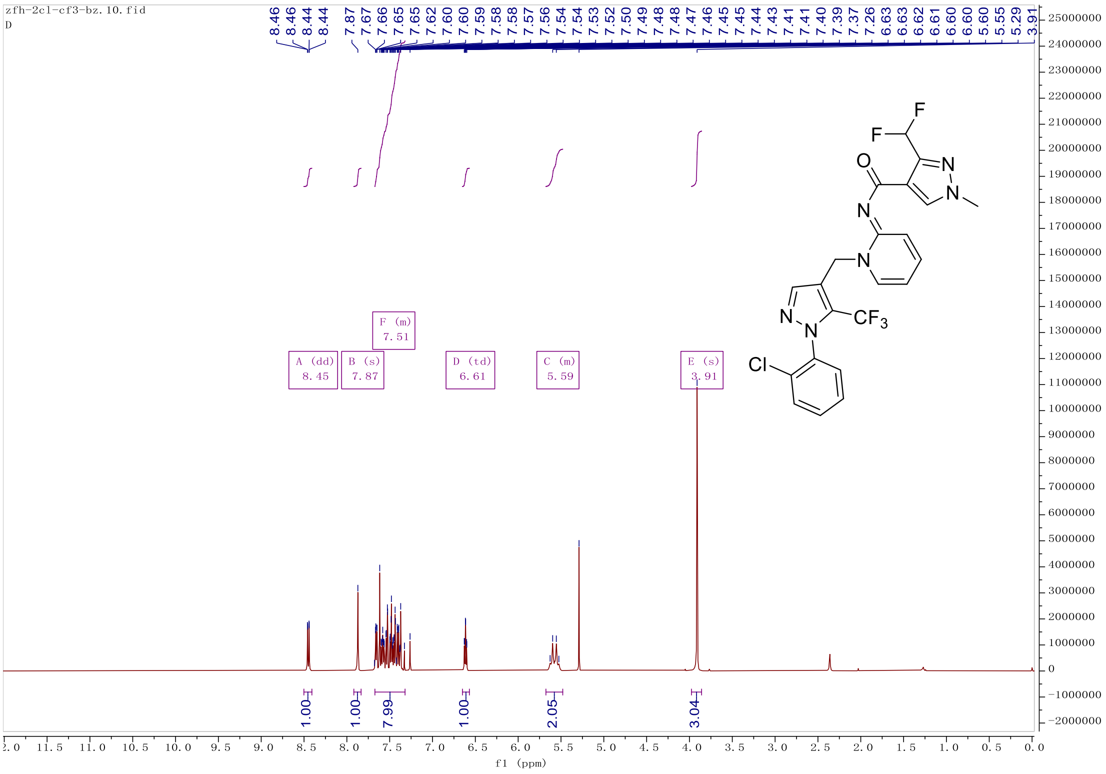

^{13}C-NMR

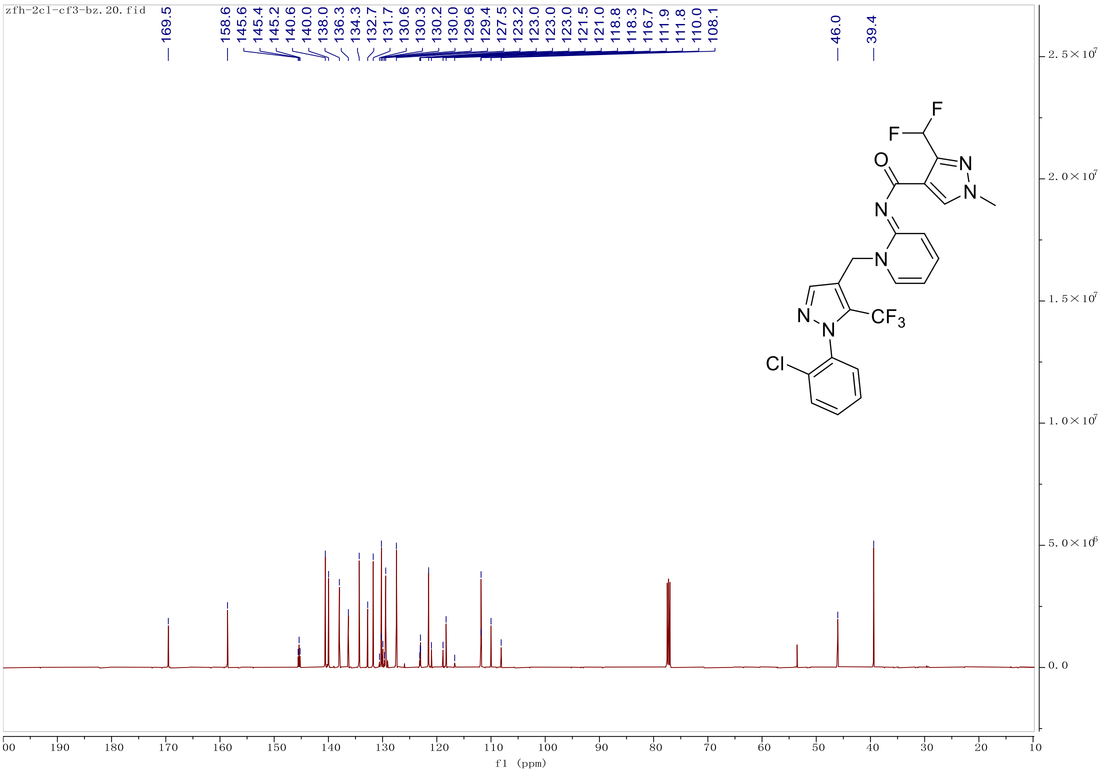

HIGH RESLUTION MASS SPECTROMETRY REPORT

Sample No.	Formula (M)	Ion Formula	Measured m/z	Calc m/z	Diff (ppm)
**D2**	$C_{22}H_{16}ClF_5N_6O$	$[M+H]^+$	511.1068	511.1067	0.20

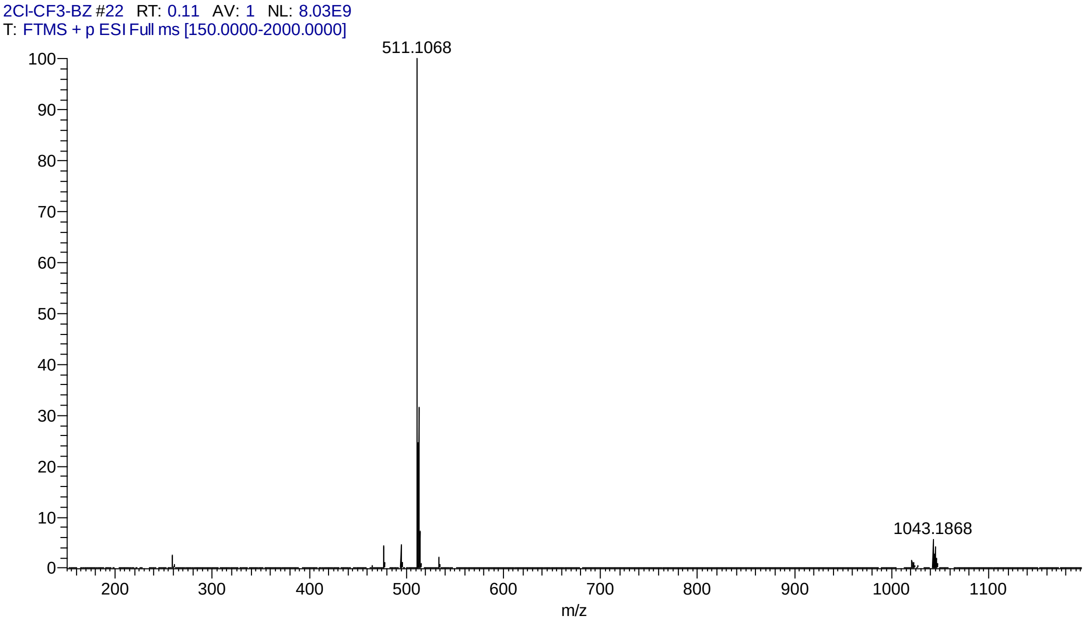

D3

H-NMR

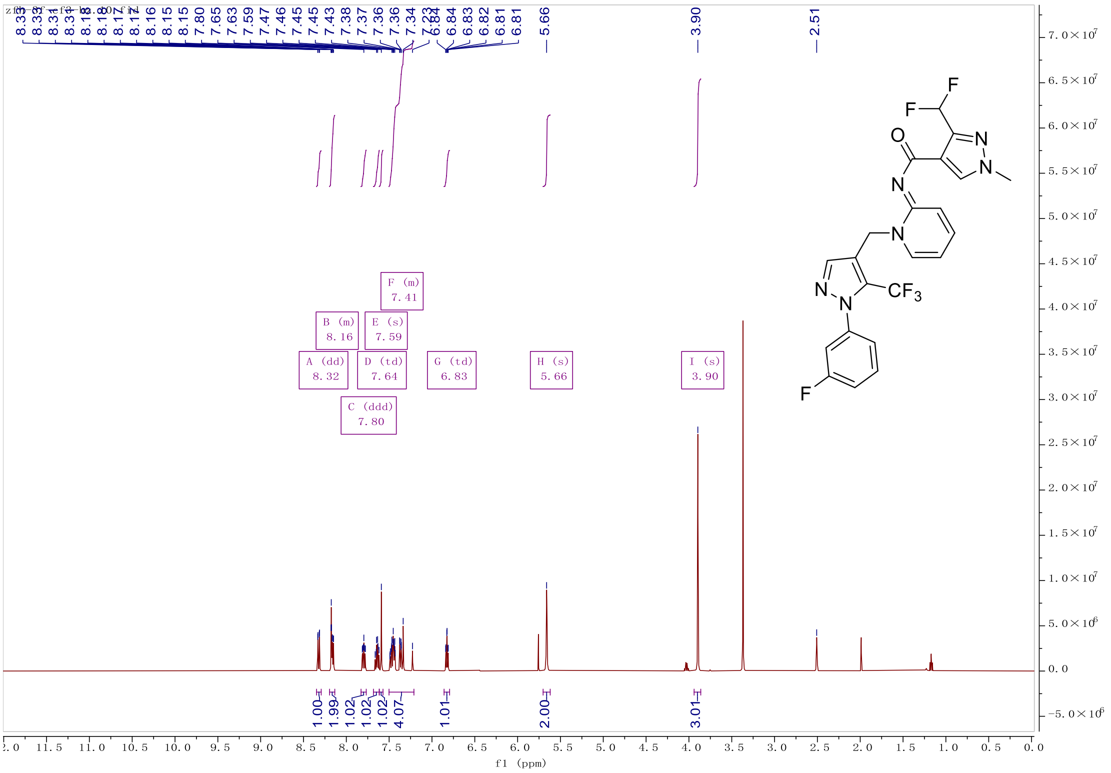

^{13}C-NMR

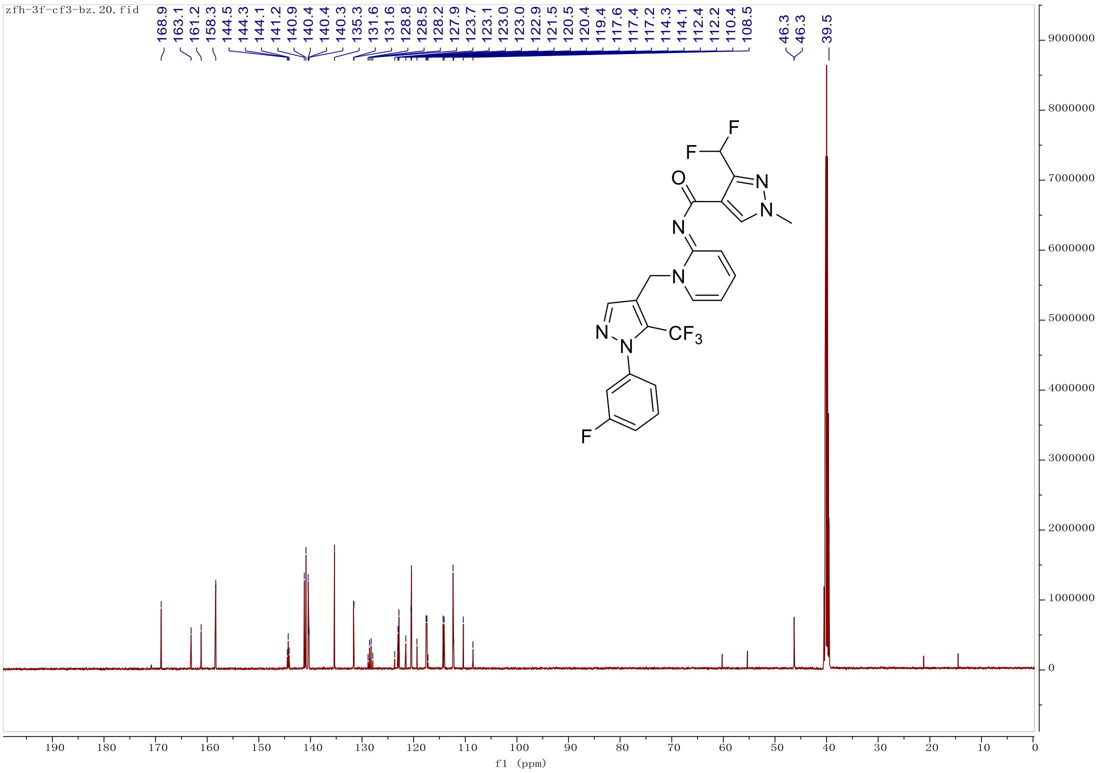

HIGH RESLUTION MASS SPECTROMETRY REPORT

Sample No.	Formula (M)	Ion Formula	Measured m/z	Calc m/z	Diff (ppm)
**D3**	$C_{22}H_{16}F_6N_6O$	$[M+H]^+$	495.1360	495.1363	-0.61

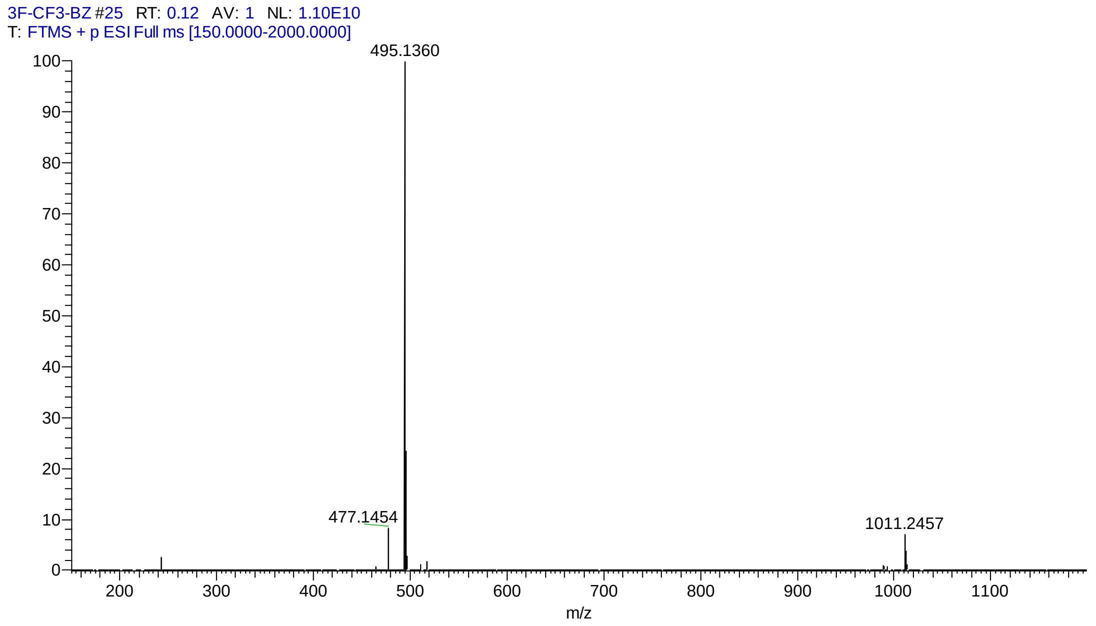

D4

H-NMR

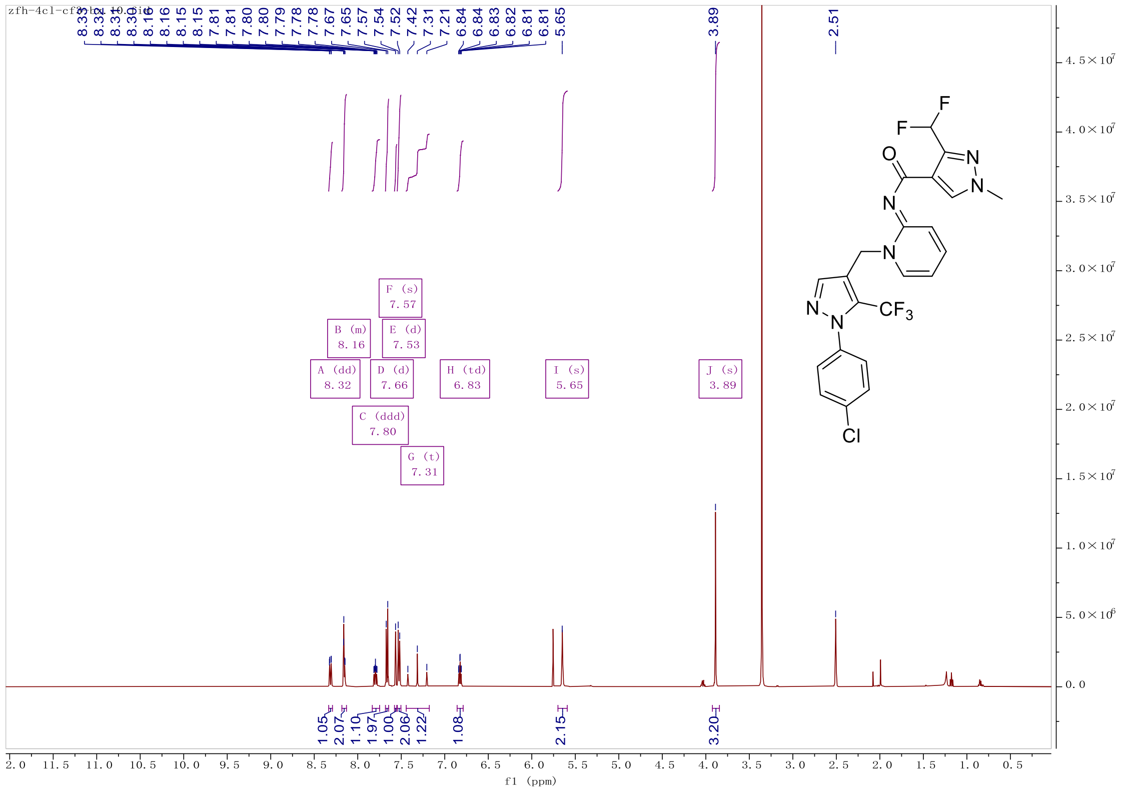

^{13}C-NMR

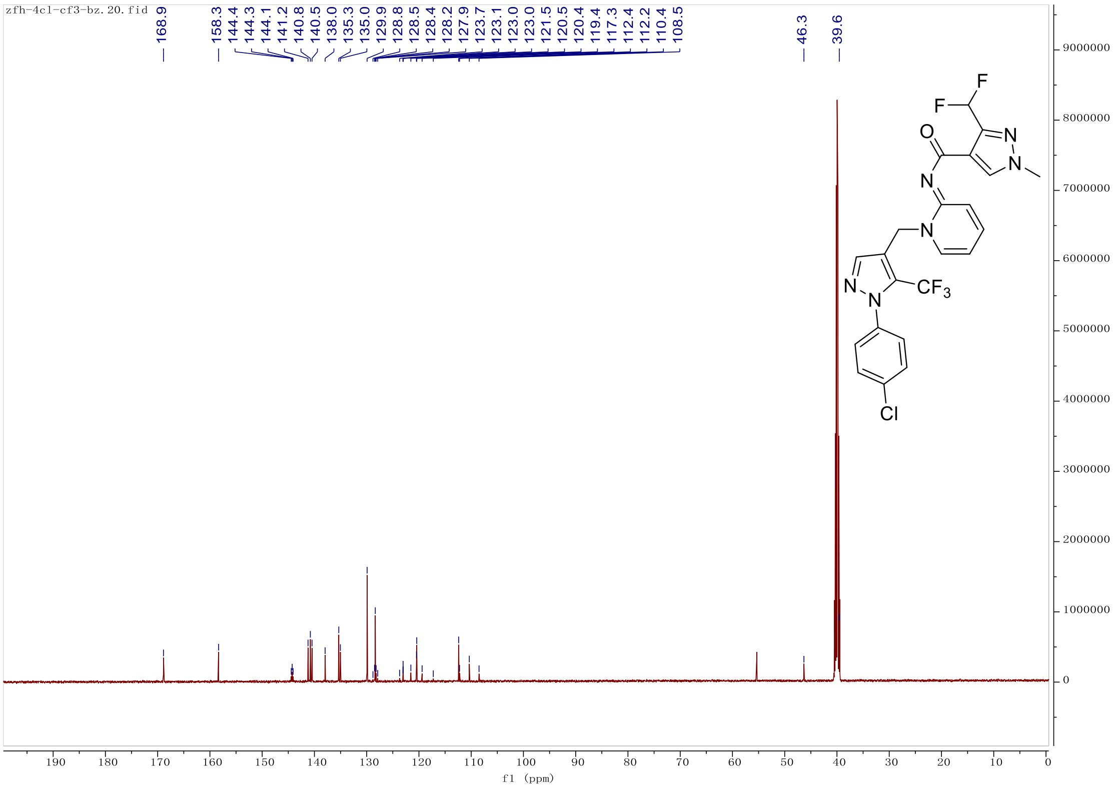

HIGH RESLUTION MASS SPECTROMETRY REPORT

Sample No.	Formula (M)	Ion Formula	Measured m/z	Calc m/z	Diff (ppm)
**D4**	$C_{22}H_{16}ClF_5N_6O$	$[M+H]^+$	511.1068	511.1067	0.20

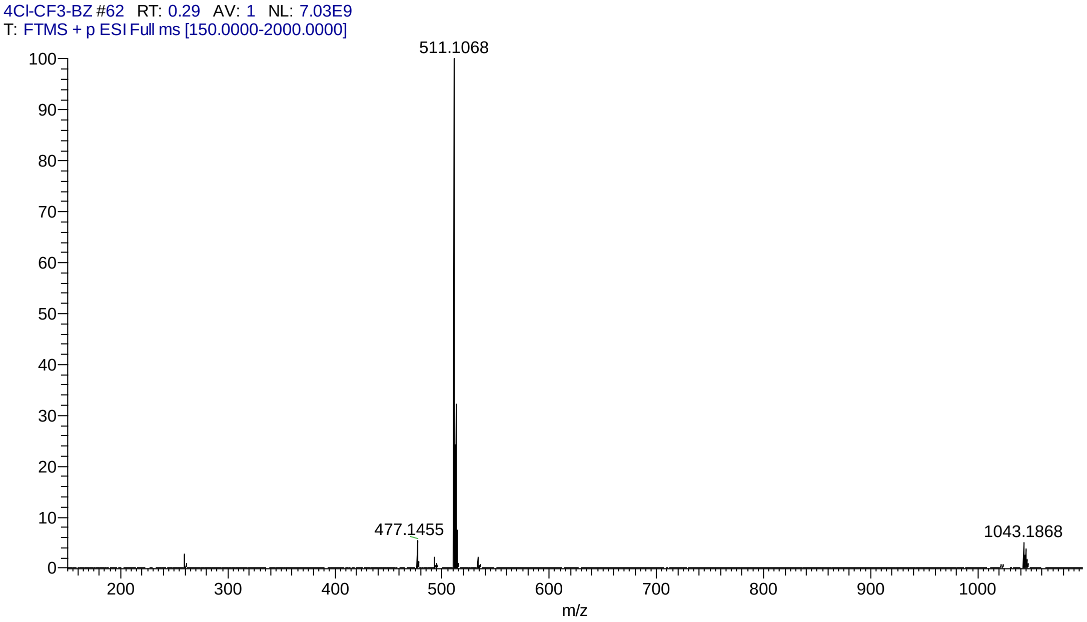

D5

H-NMR

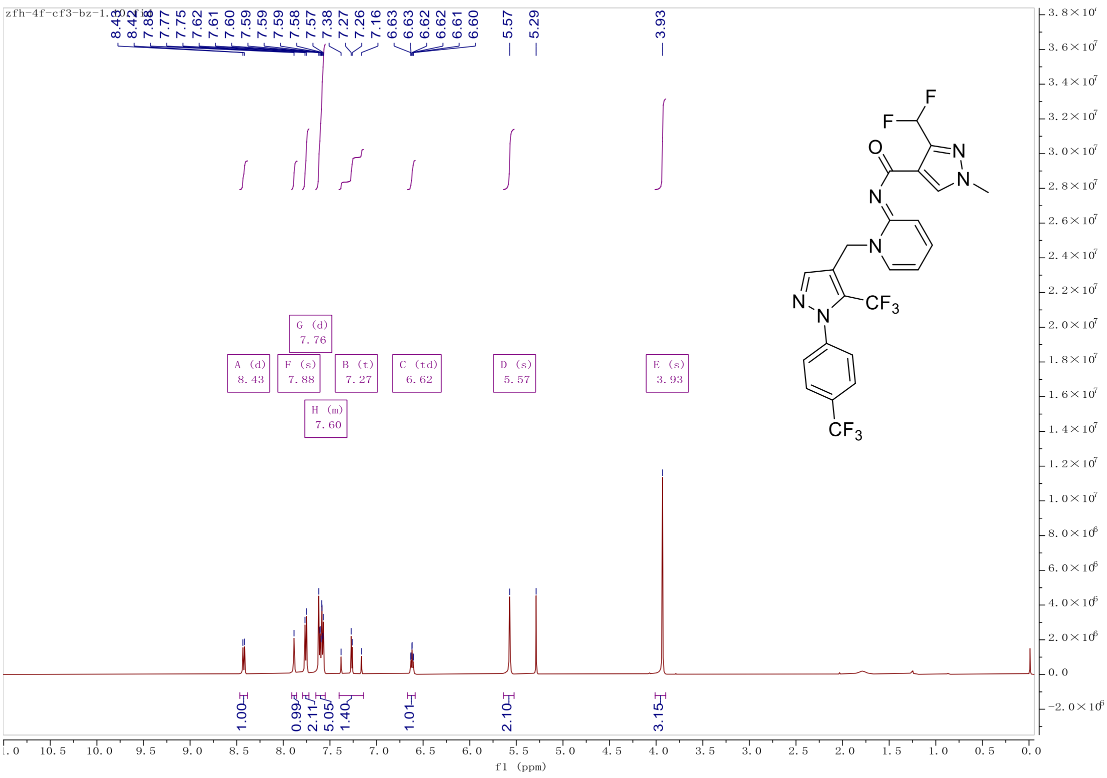

^{13}C-NMR

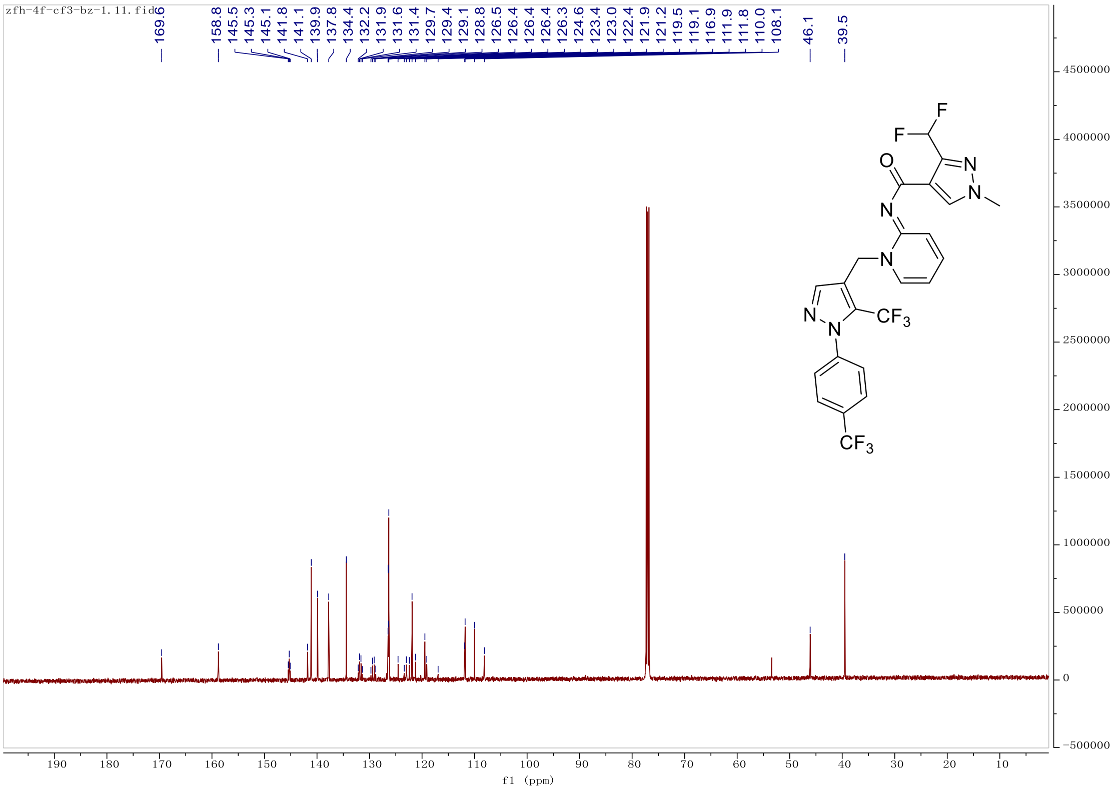

HIGH RESLUTION MASS SPECTROMETRY REPORT

Sample No.	Formula (M)	Ion Formula	Measured m/z	Calc m/z	Diff (ppm)
**D5**	$C_{23}H_{16}F_3N_6O$	$[M+H]^+$	545.1328	545.1331	0.20

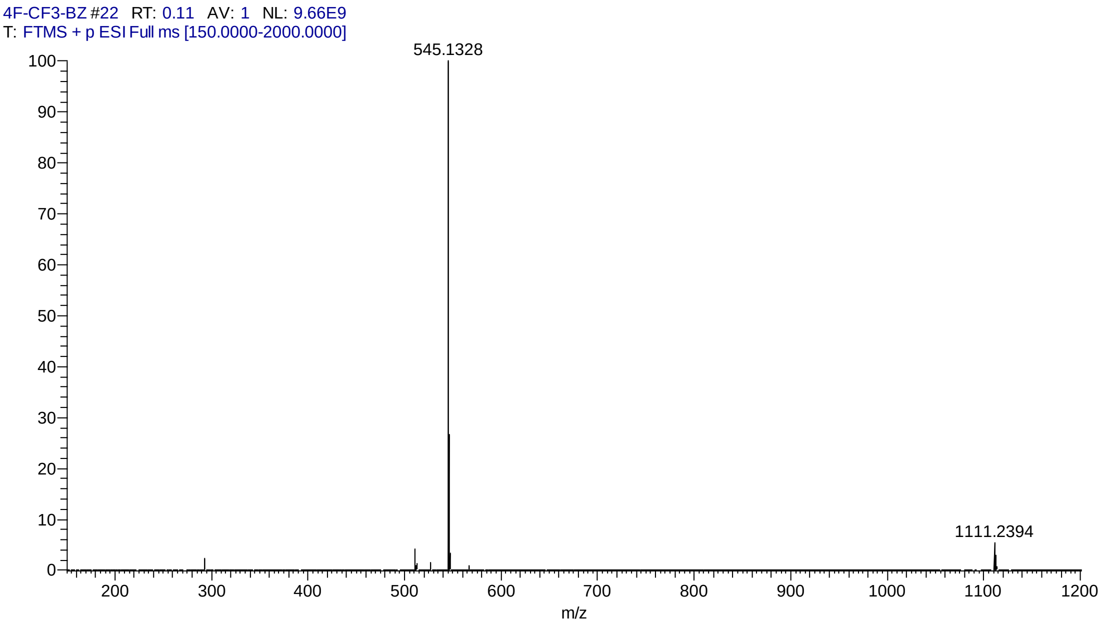

D6

H-NMR

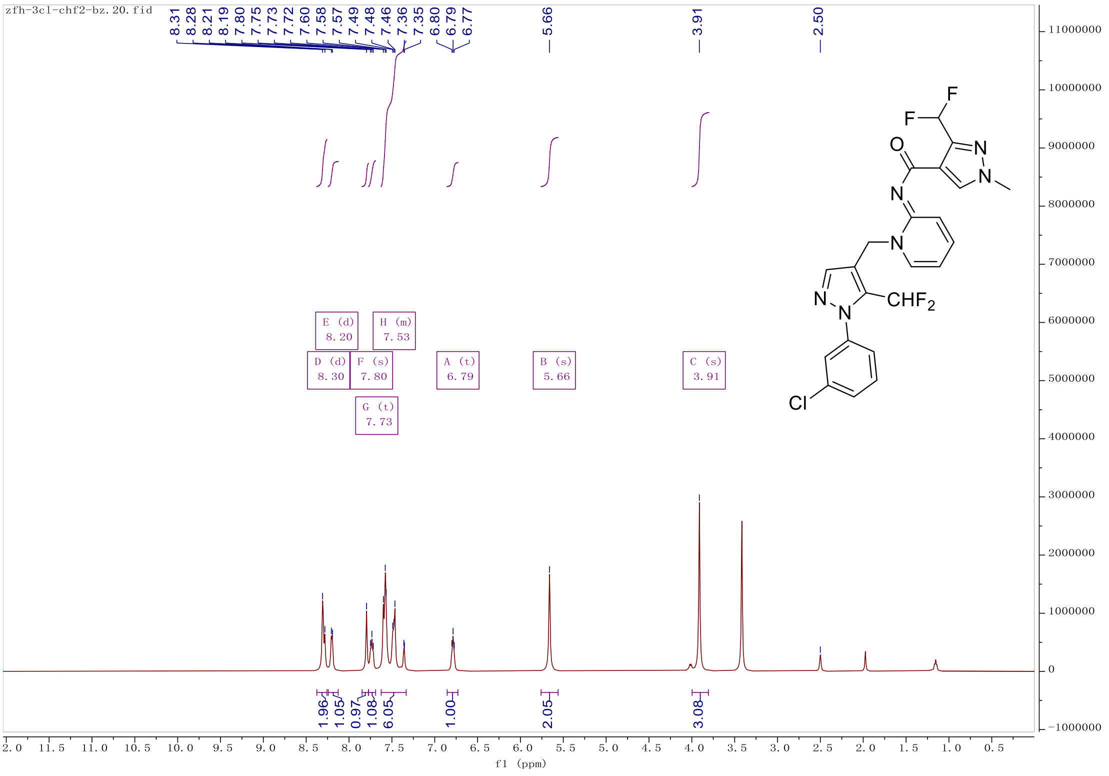

^{13}C-NMR

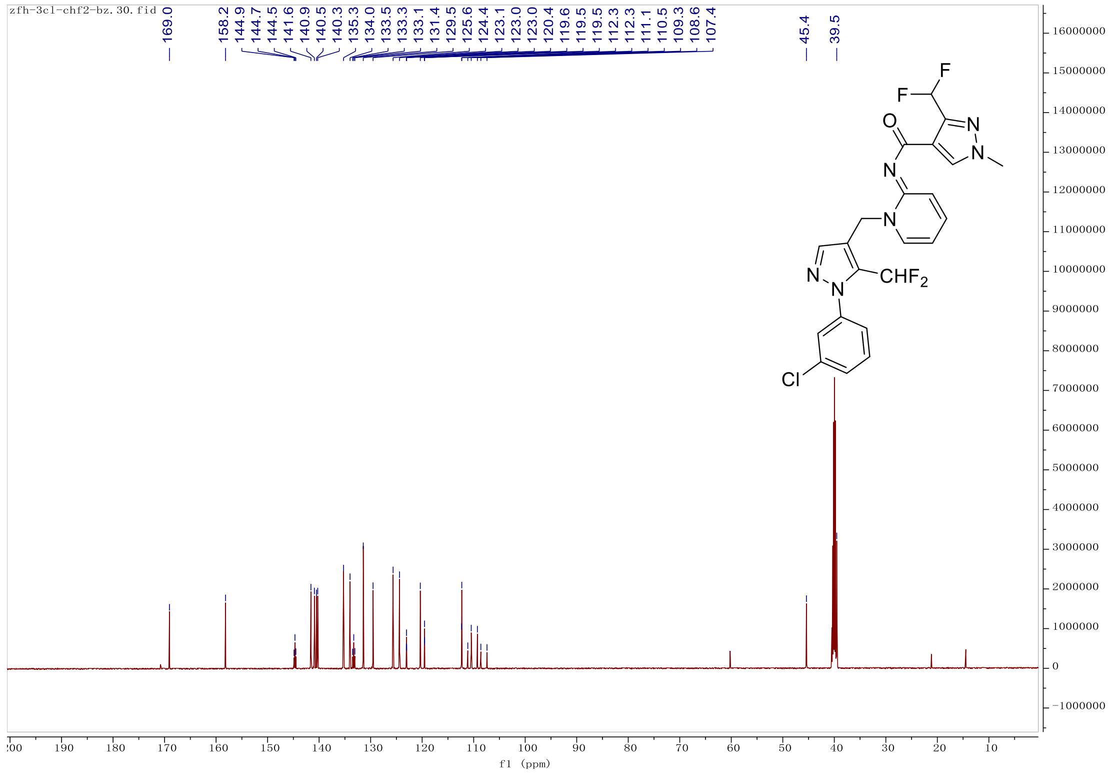

HIGH RESLUTION MASS SPECTROMETRY REPORT

Sample No.	Formula (M)	Ion Formula	Measured m/z	Calc m/z	Diff (ppm)
**D6**	$C_{22}H_{17}ClF_4N_6O$	$[M+H]^+$	493.1159	493.1161	-0.41

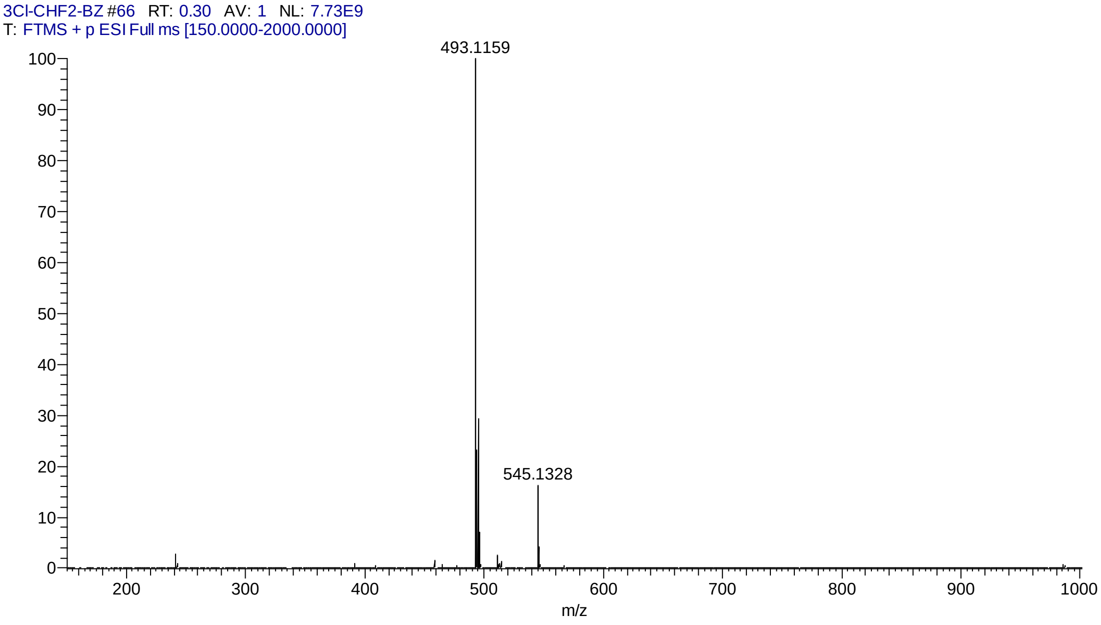

D7

H-NMR

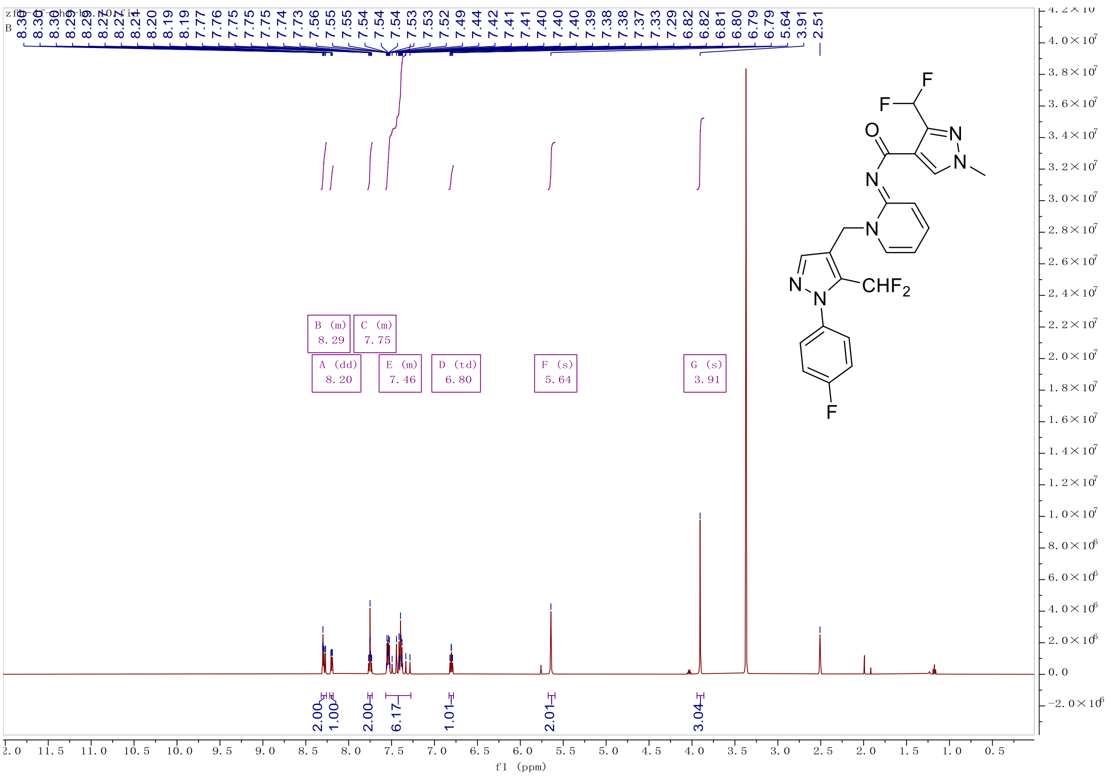

^{13}C-NMR

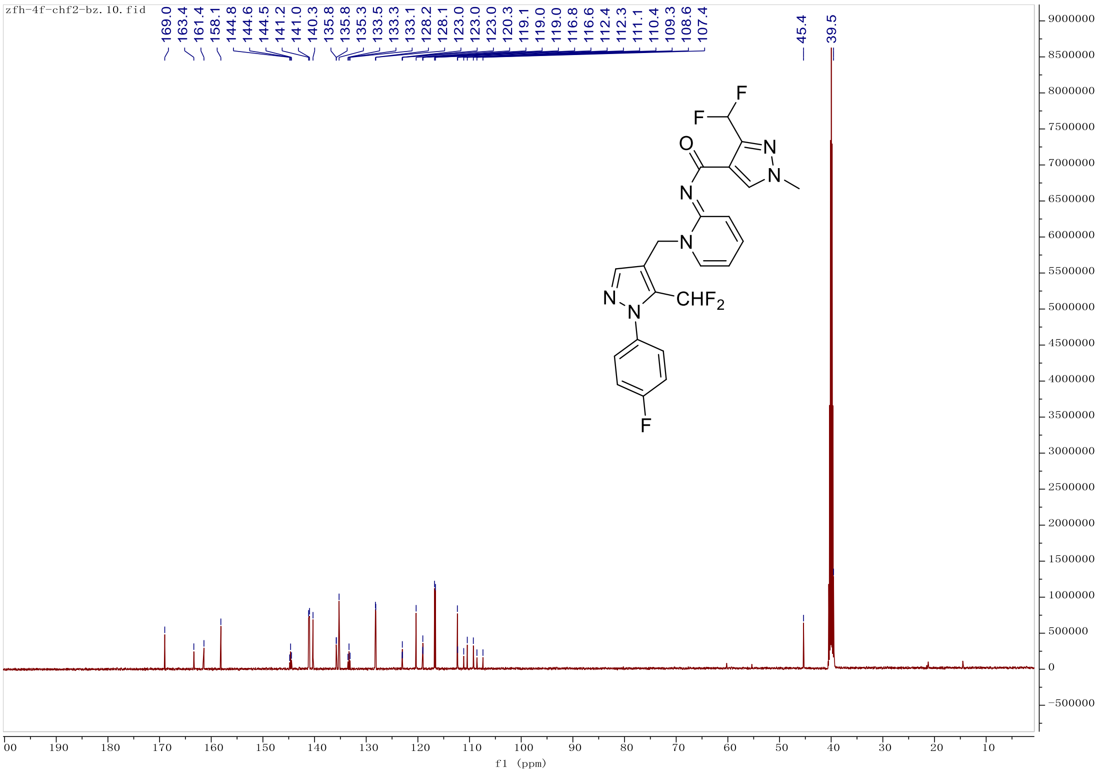

HIGH RESLUTION MASS SPECTROMETRY REPORT

Sample No.	Formula (M)	Ion Formula	Measured m/z	Calc m/z	Diff (ppm)
**D7**	$C_{22}H_{17}F_5N_6O$	$[M+H]^+$	477.1456	477.1457	-0.21

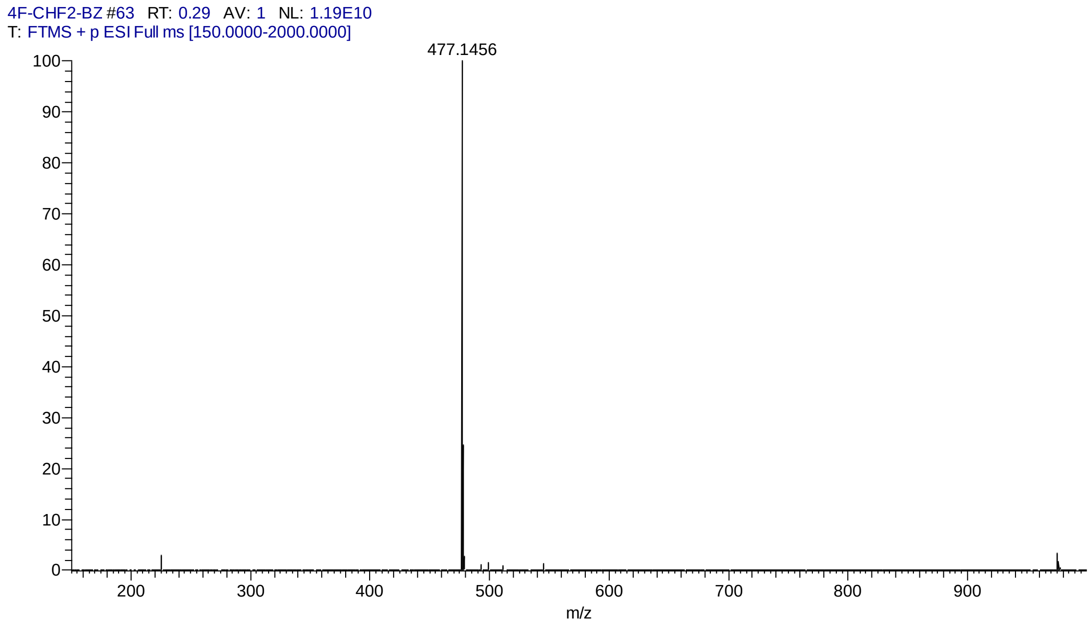

4F-CHF2-BZ #63 RT: 0.29 AV: 1 NL: 1.19E10
T: FTMS + p ESI Full ms [150.0000-2000.0000]
477.1456
100
90
80
70
60
50
40
30
20
10
0
200
300
400
500
600
700
800
900
m/z
